# Comparing Calculated Nutrient Intakes Using Different Food Composition Databases: Results from the European Prospective Investigation into Cancer and Nutrition (EPIC) Cohort

**DOI:** 10.3390/nu12102906

**Published:** 2020-09-23

**Authors:** Heleen Van Puyvelde, Aurora Perez-Cornago, Corinne Casagrande, Geneviève Nicolas, Vickà Versele, Guri Skeie, Matthias B. Schulze, Ingegerd Johansson, José María Huerta, Andreina Oliverio, Fulvio Ricceri, Jytte Halkjær, Pilar Amiano Etxezarreta, Koen Van Herck, Elisabete Weiderpass, Marc J. Gunter, Inge Huybrechts

**Affiliations:** 1 Department of Public Health and Primary Care, Faculty of Medicine and Health Sciences, Ghent University, C. Heymanslaan 10, 9000 Ghent, Belgium; heleen.vanpuyvelde@ugent.be (H.V.P.); koen.vanherck@ugent.be (K.V.H.); 2 Nutrition and Metabolism Section, International Agency for Research on Cancer, 150 cours Albert Thomas, CEDEX 08, 69372 Lyon, France; casagrandec@iarc.fr (C.C.); NicolasG@iarc.fr (G.N.); GunterM@iarc.fr (M.J.G.); 3 Research Foundation—Flanders (FWO), Egmontstraat 5, 1000 Brussels, Belgium; 4 Cancer Epidemiology Unit, Nuffield Department of Population Health, University of Oxford, Roosevelt dr, Oxford OX3 7LF, UK; aurora.perez-cornago@ndph.ox.ac.uk; 5 Department of Movement and Sport Sciences, Faculty of Physical Education and Physiotherapy, Vrije Universiteit Brussel, Pleinlaan 2, 1050 Brussels, Belgium; Vicka.Versele@vub.be; 6 Head of Nutrition Studies, Department of Community Medicine, UiT the Arctic University of Norway, Hansine Hansens veg 18, 9037 Tromsø, Norway; Guri.skeie@uit.no; 7 Department of Molecular Epidemiology, German Institute of Human Nutrition Potsdam-Rehbruecke, Arthur-Scheunert-Allee 114-116, 14558 Nuthetal, Germany; mschulze@dife.de; 8 Institute of Nutritional Science, University of Potsdam, Karl-Liebknecht-Str. 24-25, 14476 Potsdam OT Golm, Germany; 9 School of Dentistry, Cariology, Department of Odontology, Umeå University, 1D, Våning 5, Norrlands Universitetssjukhus, 901 85 Umeå, Sweden; ingegerd.johansson@umu.se; 10 Murcia Regional Health Council, Instituto Murciano de Investigación Biosanitaria (IMIB-Arrixaca), Ronda de Levante, 11., 30008 Murcia, Spain; jmhuerta.carm@gmail.com; 11 CIBER de Epidemiología y Salud Pública (CIBERESP), Av. Monforte de Lemos, 3-5. Pabellón 11. Planta 0, 28029 Madrid, Spain; epicss-san@euskadi.eus; 12 Epidemiology and Prevention Unit, Department of Research, Fondazione IRCCS Istituto Nazionale dei Tumori, Via Giacomo Venezian, 1, 20133 Milano, Italy; Andreina.Oliverio@istitutotumori.mi.it; 13 Department of Clinical and Biological Sciences, University of Turin, Regione Gonzole 10, 10043 Orbassano (TO), Italy; fulvio.ricceri@unito.it; 14 Unit of Epidemiology, Regional Health Service ASL TO3, 10095 Turin, Italy; 15 Diet, Genes and Environment, Danish Cancer Society Research Center, Strandboulevarden 49, DK-2100 Copenhagen Ø, Denmark; jytteh@CANCER.DK; 16 Ministry of Health of the Basque Government, Public Health Division of Gipuzkoa, Avda de Navarra nº 4, 20013 Donostia-San Sebastian, Spain; 17 Biodonostia Health Research Institute, Paseo Doctor Begiristain, s/n, 20014 Donostia-San Sebastián, Spain; 18 Office of the Director, International Agency for Research on Cancer, 150 Cours Albert Thomas, CEDEX 08, 69372 Lyon, France; WeiderpassE@iarc.fr

**Keywords:** food composition database, nutrient database, EPIC, ENDB, USDA

## Abstract

This study aimed to compare calculated nutrient intakes from two different food composition databases using data from the European prospective investigation into cancer and nutrition (EPIC) cohort. Dietary intake data of the EPIC cohort was recently matched to 150 food components from the U.S. nutrient database (USNDB). Twenty-eight of these nutrients were already included in the EPIC nutrient database (ENDB—based upon country specific food composition tables), and used for comparison. Paired sample t-tests, Pearson’s correlations (r), weighted kappa’s (κ) and Bland-Altman plots were used to compare the dietary intake of 28 nutrients estimated by the USNDB and the ENDB for 476,768 participants. Small but significant differences were shown between the USNDB and the ENDB for energy and macronutrient intakes. Moderate to very strong correlations (r = 0.60–1.00) were found for all macro- and micronutrients. A strong agreement (κ > 0.80) was found for energy, water, total fat, carbohydrates, sugar, alcohol, potassium and vitamin C, whereas a weak agreement (κ < 0.60) was found for starch, vitamin D and vitamin E. Dietary intakes estimated via the USNDB compare adequately with those obtained via the ENDB for most macro- and micronutrients, although the agreement was weak for starch, vitamin D and vitamin E. The USNDB will allow exposure assessments for 150 nutrients to investigate associations with disease outcomes within the EPIC cohort.

## 1. Introduction

Detailed information on the nutritional composition of foods can be found in food composition databases (FCDBs) [1]. FCDBs are usually country—or region specific, and represent a fundamental information resource for nutrition science by, for example, estimating exposure to various food components with both positive and negative health outcomes [1,2,3]. However, when estimating dietary intake across multiple countries with different eating cultures and traditional diets, the lack of a single standardised dietary database that provides internationally comparable nutritional data poses the methodological challenge of how to determine the nutrient content of consumed foods [3,4].

In Europe, considerable efforts have been made to harmonise national FCDBs [3,5,6]. Within the frame of the European prospective investigation into cancer and nutrition (EPIC) study, the EPIC nutrient database (ENDB) project was conducted between 2002 and 2005. The ENDB was a pioneer project for the harmonisation of food composition data across 10 European countries [7], resulting in an end-user nutrient database including information on 28 food components. 

Despite the effort made through the European projects to harmonise European FCDBs, many national FCDB still have a limited food list, lacking information for many food items, especially on micro-nutrients, and are still not comparable for important food components, due to methodological variations [3,4]. These variations may include different analytical approaches and methods for nutrient calculations, definitions of nutrients and units of measurement [4,8]. As a result of the limitations found in national FCDB, extending the ENDB with extra food components using the EPIC country-specific FCDBs is not feasible, and would introduce substantial measurement error in dietary intakes. This measurement error may lead to non-differential misclassification of exposure and reduced power to detect associations with disease-outcomes.

As part of the EPIC study, a new dietary intake database has now been compiled to investigate the intake of a broader range of nutrients than initially covered by the ENDB project (e.g., individual fatty acids, amino acids, individual sugars). For this, the EPIC dietary intake data was matched to the U.S. nutrient database (USNDB, National Nutrient Database for Standard Reference of the U.S. Department of Agriculture—USDA), which includes more than 8000 foods and 150 food components. [9]. 

As a matter of relative validation, the aim of this study was to compare the already available dietary intakes of 28 in the ENDB with the same nutrients from the USNDB. The selection of the USNDB as a more extensive source of food components reflects a pragmatic approach when more comprehensive standardisation of national FCDBS is not feasible, and will allow exposure assessments for 150 nutrients to investigate associations with disease outcomes within the EPIC cohort.

## 2. Materials and Methods 

### 2.1. EPIC Study Design

EPIC is a large on-going multicentre prospective cohort study consisting of 521,324 adults (366,521 women and 153,437 men) mostly aged 35–70 years at recruitment [10]. The objective of this cohort was to investigate the role of diet, lifestyle, metabolic factors and genetics in cancer development, as well as other non-communicable diseases [10,11]. Study participants were enrolled between 1992 and 2000 from 23 centres across 10 European countries: Denmark, France, Germany, Greece, Italy, The Netherlands, Norway, Spain, Sweden and the United Kingdom. EPIC’s study rationale, study population and data collection have been described elsewhere [10,11]. All participants provided written informed consent and the ethical review boards from the International Agency for Research on Cancer (IARC—Lyon, France) and from all local centres approved the study. 

### 2.2. Dietary Intake Assessment Methods

The collection of long-term dietary intake data was conducted at baseline through country or centre-specific and validated dietary questionnaires (DQ), spanning the previous 12 months, and designed to capture geographical specificity of the diet [10]. In most centres, DQs were self-administered food-frequency questionnaires, with the exception of the centres in Ragusa (Italy), Naples (Italy) and Spain, where food-frequency questionnaires were administered by face-to-face interviews [10]. Using different dietary assessment methods across study countries and centres, may induce systematic and random errors in dietary intake measurement when the dietary data from the different countries is combined. To address this issue, a calibration approach was developed to adjust for possible systematic over- or underestimation in dietary intake measurements [12]. This approach included a single 24-h dietary recall (24-HDR), conducted by trained interviewers using a computer-assisted, interactive dietary interview program (EPIC-soft) [13]. This procedure was standardised within and between all EPIC centres. The 24-HDR was collected at baseline for a representative sample (N = 36,994) of the entire EPIC cohort [12].

### 2.3. Initial Compilation of a Harmonised Nutrient Database for the EPIC Project

The ENDB aimed to harmonise the nutrient values of the national FCDBs across the 10 participating EPIC countries and originally focussed on energy and 26 nutrients [7]. An inventory of nutrient definitions, methods of analyses and modes of expression among the FCDBs in nine EPIC countries formed the basis of the ENDB [4]. Since 2010, a folate database has been compiled as an extension of the ENDB [14], based on a new inventory and critical evaluation of folate data in 15 European and three non-European FCDBs [15]. The ENDB counts 28 fully documented food components today (Table 1).

### 2.4. Matching of the EPIC Food List with the USNDB

To match the EPIC food list with the USNDB, we used the USNDB release 26 (October 2013) with 8463 food items and 150 food components, with further completion using the 28th release (September 2015) containing 8789 food items. The USNDB has great transparency on the source of nutritional data, ensures documentation about the methods, definitions and calculation—and imputation methods used, and gives information on the data quality assessment for all analytical nutrient profiles [9].

The procedure to match the EPIC food list with the USNDB builds upon the standardised procedure used to compile the ENDB [7,14]. In brief, consumed foods derived from the dietary assessments in EPIC were matched as closely as possible to foods available in the USNDB. Nutrient values of foods unavailable in the USNDB were either estimated by recipe calculation, or by weighted averaging, i.e., the weighted average of the consumption frequencies of related foods was calculated (e.g., vegetable oil.: weighted average of vegetable oils including olive oil, rapeseed oil, corn oil). 

### 2.5. Quality Assessment of the Matching Procedure

Three quality controls were performed which guaranteed the accuracy of the matching procedure, linking EPIC food data to the USNDB. First, a random sample of food items was matched in duplicate to the USNDB by two researchers independently. Secondly, the fully matched food list and the assigned nutrient values were checked for errors once by an accredited nutritionist and once by an expert of the ENDB project. Finally, systematic quality controls were performed based upon the distributions of intakes. Extreme intake values were inspected and identified errors were corrected.

### 2.6. Statistical Analyses

The reported food intakes of 476,768 participants for the DQ data (whole EPIC sample) and 34,064 participants for the 24-HDR data (EPIC sample with 24-HDR data) were analysed. For the DQ data, participants with missing information on more than 80% of the relevant dietary questions (N = 6837) and participants with implausible energy intakes (i.e., the top and bottom 1% of the distribution of the ratio of reported total energy intake to energy requirement; N = 10,242) were excluded from the analyses. No participants were excluded for analyses concerning the 24-HDR data, because of its detailed and standardised nature and built-in quality controls. Data from Greece were not included in this study. Missing nutrient values were replaced by zeros to allow the calculation of nutrient intakes for all subjects.Mean dietary intakes of energy and the other 27 components, their standard deviations (sd) and median were calculated using the USNDB and the ENDB. Differences in dietary intakes were reported as absolute mean differences and paired samples t-tests were conducted. Pearson correlation coefficients were performed to investigate the associations of dietary intakes estimated using the USNDB with the ENDB. As a measurement of agreement between both methods, rather than a measurement of differences, Bland-Altman plots were presented for each of the nutrients and their corresponding limits of agreement [16], using the Statistical Package for the Social Sciences (SPSS Inc., Chicago, IL, USA; version 26). Weighted kappa coefficients (κ) were calculated to assess the agreement on the classification of individual dietary intakes into quintiles. Cut-offs for quintiles of equal size were assigned separately for the USNDB and ENDB, based on the distribution of dietary intake of each food component. In this study, it was decided to be stricter with the interpretation of the weighted kappas than suggested by Cohen [17], considering the following interpretation: 0.01–0.39 as none to slight, 0.40–0.59 as weak, 0.60–0.79 as moderate, 0.80–1.00 as strong to very strong agreement.

All statistical tests were two-sided, with a statistical significance level of α = 0.05 and carried out with the Statistical Analysis Software (SAS Institute Inc., SAS Campus Drive, Cary, NC, USA) version 9.4, unless otherwise specified.

## 3. Results

Table 2 shows the mean dietary intakes, their standard deviation and the median dietary intakes of energy and 27 nutrients, as estimated by the USNDB and the ENDB, and the absolute difference in mean nutrient intakes. Differences in mean nutrient intake between the USNDB and ENDB were statistically significant (paired samples *t*-test: *p* < 0.001) for all nutrients. Results per country can be found in Table S1. Concerning the DQ data, dietary intakes for energy, total fat and carbohydrates estimated by the USNDB were higher compared to dietary intakes calculated by the ENDB. For proteins, dietary intake measurements by the USNDB were lower. Absolute mean differences for energy and the three principal classes of macronutrients between the USNDB and ENDB were 61.2 kcal/day (3% relative to the ENDB) for energy intake, 1.2 g/day (1.5% of ENDB) for total fat, −4.3 g/day (−4.9% of ENDB) for proteins and 24.0 g/day (10.4% of ENDB) for carbohydrates. Similar results were found for the 24-HDR, the mean difference between the USNDB and ENDB for energy intake was 20.2 kcal/day (1% relative to the ENDB), −0.2 g/day (−0.2% of ENDB) for fat, −6.1 g/day (7.2% of ENDB) for proteins and 17.4 g/day (7.6% of ENDB) for carbohydrates. The strongest mean difference in dietary intake between the USNDB and the ENDB was found for starch: −72.3 g/day (−59.3% relative to the ENDB) and −76.8% (−64.7% relative to the ENDB) for the DQ data and 24-HDR data, respectively. A higher/lower dietary intake measure by the USNDB compared to the ENDB for the DQ data was systematically presented as a respective higher/lower dietary intake by the USNDB for the 24-HDR, except for the nutrients with a mean difference between −5% and 5%.

Pearson correlation and weighted kappa for dietary intakes of energy and 27 priority nutrients between the USNDB and the ENDB can be found in Table 3. Results per country can be found in Table S2. Regarding the DQ data, Pearson correlation coefficients for the associations of nutrient intakes estimated using the USNDB and the ENDB ranged from r = 0.62 for vitamin E (alpha-tocopherol) to r = 1.00 for water. For most of the nutrients, strong to very strong correlations were found (r ≥ 0.80). Moderate correlations (r = 0.60–0.79) were only found for thiamine (r = 0.78), magnesium (r = 0.78), starch (r = 0.72) and vitamin E (r = 0.62). Similarly, for the 24-HDR data, strong to very strong correlations were found for most of the nutrients, followed by moderate correlations for vitamin B6, food folate, magnesium, retinol, iron, and vitamin E. Only for vitamin B1 (r = 0.56), starch (r = 0.54) and vitamin D (r = 0.48) weak correlations were found.

Results of the weighted kappa analysis for the DQ data ranged from κ = 0.43 for starch to κ = 0.98 for water. Energy intake, total fat, protein and carbohydrate intakes showed strong to very strong agreement (κ = 0.80–1.00). Moderate agreement (κ = 0.60–0.79) was found for the majority of the nutrients and only starch, vitamin D and vitamin E showed weak agreement (κ = 0.40–0.59). Regarding the 24-HDR, weighted kappas were lower compared to those of the DQ data and ranged from κ = 0.30 for starch to κ = 0.96 for water. Strong to very strong agreement was only shown for water, energy, fat and alcohol and a much larger share of nutrients showed a weak agreement (iron, magnesium, vitamin D, vitamin E, vitamin B1, vitamin B2 and vitamin B12).

**Figure 1:**
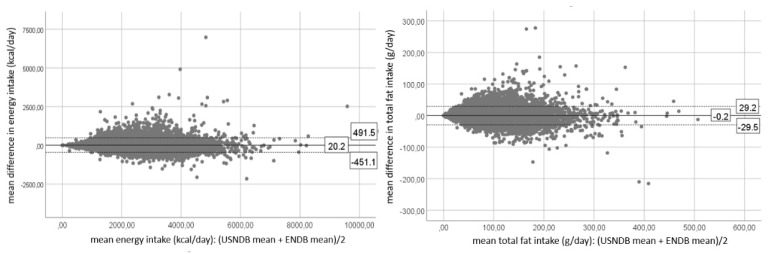

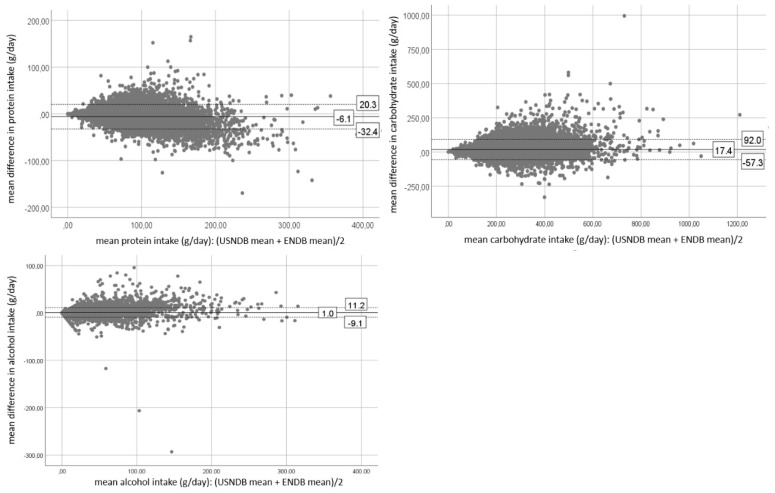
Bland-Altman plots for the 24-h dietary recall (24-HDR) data representing the mean differences and their limits of agreement for energy, total fat, proteins, carbohydrates and alcohol intakes between the U.S. nutrient database (USNDB) and the EPIC nutrient database (ENDB). The mean difference is represented by the full line, the upper and lower limit of agreement are presented by dotted lines.

**Figure 2:**
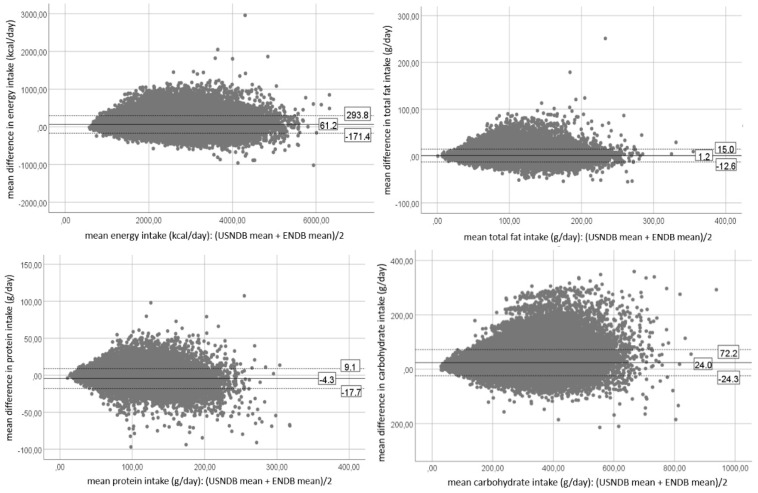

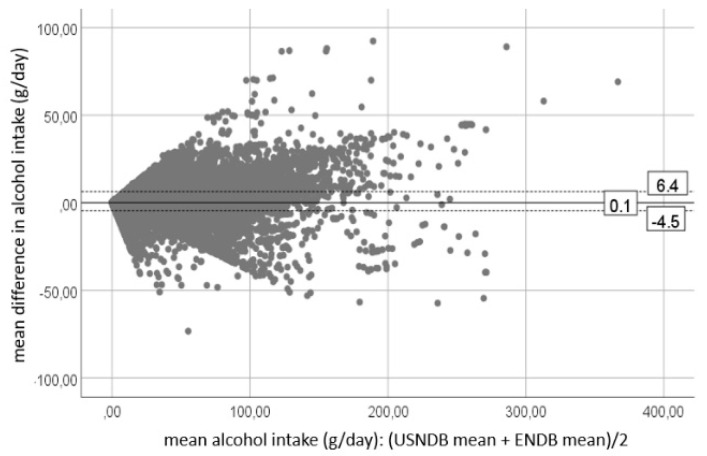
Bland-Altman plots for the dietary questionnaire (DQ) data representing the mean differences and their limits of agreement for energy, total fat, proteins, carbohydrates and alcohol intakes between the U.S. nutrient database (USNDB) and the EPIC nutrient database (ENDB). The mean difference is represented by the full line, the upper and lower limit of agreement are presented by dotted lines.

Bland-Altman plots for energy, total fat, proteins, carbohydrates and alcohol intakes for the 24-HDR data and DQ data are presented in Figure 1 and Figure 2, respectively. Bland-Altman plots for all other nutrients can be found in Figure S1 for the 24-HDR data and in Figure S2 for DQ data. The mean difference, or bias, is the same as the mean difference presented in Table 2. Visual inspection of these Bland-Altman plots shows a divergent pattern for the majority of the nutrients (e.g., cholesterol intake, iron intake, magnesium intake, vitamin E intake, retinol intake), which reflects an increasing mean difference with increasing intakes.

## 4. Discussion

### 4.1. Main Results and Interpretation

Matching EPIC dietary intake data to the USNDB will allow exposure assessments for 150 food components to investigate in relation to disease outcomes within the EPIC cohort. Comparative analyses showed significant, but rather small, absolute differences between dietary intakes of the 28 selected nutrients estimated by the USNDB and the ENDB for participants in the EPIC study. Among the three classes of macronutrients, the greatest mean difference was found for carbohydrates (10.4% difference for DQ data and 7.6% difference for 24-HDR relative to the ENDB), representing higher carbohydrate intake estimates by the USNDB than by the ENDB. Within the USNDB, data on total carbohydrates was calculated ‘by difference’ (i.e., the difference between 100 and the sum of the percentages of water, protein, total fat, ash and, when present, alcohol), and includes total dietary fibre [18]. Within the ENDB, the sum of analysed fractions was the reference method, excluding dietary fibre (i.e., glycaemic carbohydrates), whereas values obtained by difference were graded as ‘non comparable’ [7]. Note that the carbohydrate values were considered as possibly presenting the most heterogeneity in terminology, definition, mode of expression and methods used (analytical or calculations) across EPIC countries [4]. These differences in definition and calculation methods likely explain the absolute difference in carbohydrate intakes between the USNDB and the ENDB. The greatest mean difference was shown for starch, a fraction of carbohydrates, with much higher estimates reported by the ENDB. In addition to the heterogeneity described in carbohydrate values across European FCDB, the level of detail with regard to the coverage of the different carbohydrate fractions varies significantly across European countries [4]. Therefore, the starch values reported in both the USNDB and ENDB should be handled with caution. A short overview of the USNDB and ENDB reference component-specific definition and standard analytical methods and approaches for all 28 nutrients is shown in Table S3.

Relative differences in dietary intake estimates between the USNDB and the ENDB were examined using Pearson correlations. Moderate to very strong correlations for the majority of the food components under study demonstrate a good ranking of the subjects according to their nutrient intake. However, Pearson correlation coefficients can be misleading when assessing agreement, because the significant correlations describe a linear relationship between two sets of data, but do not necessarily imply good agreement between the USNDB and ENDB. Therefore, Bland-Altman plots were used to describe the agreement between the two methods. The divergent pattern shown in the majority of the Bland-Altman plots indicates an increase in mean difference with increasing intakes.

In addition to Bland-Altman plots, the results of the weighted kappa analysis indicated strong agreement for the three principal classes of macronutrients and moderate agreement for the majority of the other nutrients for the DQ data. However, weighted kappas of the 24-HDR data were lower compared to those of the DQ data. Overall, this project shows a good level of agreement for energy intake and the majority of the nutrients, although not for starch, vitamin D, vitamin E and thiamine.

In addition to the specific arguments for carbohydrate related compounds explained above, some more generic issues in the matching of dietary intake data with food items available in the FCDBs may further explain the absolute and relative differences found in this comparison between dietary intakes estimated by the USNDB and ENDB. Three major issues in the matching with FCDB can be addressed. 

First, there are significant differences in the food lists (e.g., number of foods, kinds of food stuffs, level of detail) available in the different FCDBs. Matching European food data, which have country- and local-specific unique foods and dishes, to the USNDB is not unequivocal. The lack of exact food matches between the food items available in the EPIC food list and the USNDB has led to difficult and arbitrary decisions that had to be made during the matching procedure. This issue also arose during the compilation of the ENDB. The food lists available in the national FCDBs, originating from the late 1990s and early 2000s, were, for some countries, limited in the number of food items available, requiring them to borrow food composition data from neighbouring countries [7]. 

Second, differences could be caused by advancements and variation in definitions and laboratory technologies to measure the different nutrients. Indeed, more advanced laboratory methods available in the past few years may also contribute to differences in values between the different FCDBs. These differences in methodologies already complicated the harmonisation of the different national FCDBs used in the ENDB, as various methods had been used across Europe. Considering the further advancements in technologies over the past decades, these innovations may contribute to the differences found between the more recent USNDB and the ENDB.

Third, changes over time in product composition, processing and potential changes in national food regulations may result in a variation of nutrient content of (processed) foods over time. Furthermore, geographical and environmental variations are likely to exist between the same foods in the different national FCDBs, especially in the vitamin and mineral content. These differences in food composition are likely to be found between continents, between European countries, and even between foods originating from the same grower or manufacturer and/or over different harvests (e.g., due to differences in species, exposure to sunlight or pesticides, storage conditions and period). Considering the current global food system, with import and export of foods between regions, matching with a non-European database that delivers standardised and high-quality food composition data was appraised as a pragmatic and scientifically justifiable solution.

Since agreement was higher for energy and macronutrient intakes than for certain micronutrient intakes, it is likely that the vitamin and mineral content of food is the most vulnerable to environmental and climatic conditions, food processing and regulations and/or the analytical method used. This is particularly the case for unstable components (i.e., labile to temperature, pH and oxidation), leading to potential problems in the accurate measurement of these nutrients.

### 4.2. Comparison with Similar Studies

Only few studies have examined the dietary intake of an adult population by different FCDBs [19,20,21,22,23], and most of them focused on dietary intakes measured by different European FCDBs [19,20,21]. Good correlations for macronutrients (r > 0.70) were reported by most of these studies comparing different European FCDBs [19,20,22,23]. Although one comparative study suggested a discrepancy between FCDBs for energy, fat and carbohydrate intakes [21]. Only one study examined the level of agreement between macro- and micronutrients of the USNDB (modified by Chilean food items) and the British FCDB [23]. This study concluded that results for dietary intakes are similar for the USNDB and the British FCDB for the majority of the nutrients under study. However, the USNDB tends to give relative overestimates of macronutrients in comparison to the British FCDB, but such a trend was inconsistent for micronutrients [23]. Within the EPIC cohort, similar results were found when comparing dietary intakes by the USNDB and ENDB. However, the magnitude of the overestimation of macronutrients was rather low, ranging between 1.5% to 10% higher intakes relative to the ENDB, and no such trend was observed for micronutrients. Micronutrients are less likely to be expressed in comparable ways by means of their methodological nature (analytical methods used, definition, and measurement units) across different FCDBs [4,7,19,20,21,22,23].

### 4.3. Recommendations for Future Studies and Food Composition Data Compilers

The EuroFIR (European food information resource) project and INFOODS (International Network of Food Data Systems) are appreciated for their efforts in promoting international cooperation and harmonisation of standards to improve data quality, availability and reliability [24,25]. Unfortunately, national FCDBs are not necessarily conceived to provide internationally comparable data or internationally interchangeable data, which is a major constraint for multi-centric studies that require standardisation across continents and regions [26]. Therefore, international (cross-continental) efforts are needed to study, inventory and eventually put into practice reference analytical methods for assessing the nutrient contents of foods and the use of universal definitions, measurement units and classification into food groups. Once settled, European FCDB could benefit from this, and expand their number of food components. However, high quality local food composition data remains important, especially when it comes to typical local food products and dishes or for countries that are mainly self-sufficient for their food supplies.

### 4.4. Strengths and Limitations of the Comparison Study

A major limitation of this comparison study is the lack of a gold standard, since both approaches, i.e., estimating dietary intake using the USNDB or the ENDB, are prone to error. Furthermore, the results of the relative validation study for the 28 food compounds might not be generalisable to the remaining 122 food components of the USNDB. The lack of nutritional composition data for several food items for less common nutrients should be taken into account. It may affect exposure estimations (underestimation of true intakes) and lead to the attenuation of associations found between nutrient intakes and health outcomes. Despite these limitations, this study has several strengths. To match the EPIC food list to the USNDB, a standard procedure was maintained, building on the previous experiences of the ENDB project [7,14]. To assure the continuation of the standard approach, quality controls were built in during the matching procedure. The strong methodology of the EPIC study allowed assessment of the relative validity in duplicate, using the 24-HDR and the DQ and taking advantage of its large sample size. Furthermore, matching the EPIC food list to the USNDB is a strong added value to the EPIC study, and is of crucial importance: providing up to 150 food components to the EPIC cohort dataset will give the opportunity to investigate additional risk factors for specific cancers and other chronic diseases.

### 4.5. Recommendations for Users of the EPIC Nutrients Database

If the dietary nutrients of interest are available in the ENDB, it is recommended to make use of the ENDB data, as this enables interpretation of the results in the context of the previously published work in EPIC. In case one or more nutrients are not available in the ENDB, it is recommended to use the USNDB exclusively for all nutrients as mixing both approaches is discouraged in order to avoid discrepancies between nutrients (e.g., total energy intake should remain the sum of the energy of the different macronutrients). For nutrients with rather weak agreement (κ < 0.60) between the USNDB and ENDB, researchers should be prudent in using the data from both the USNDB and the ENDB, because both databases have limitations, and sensitivity analyses using the nutrients from both databases could be suggested.

## 5. Conclusions

In this study, the EPIC dietary assessment data was matched to the USNDB. Good agreement was shown between the USNDB and the original ENDB for energy intake, total fats, proteins, carbohydrates, sugar, alcohol, potassium and Vitamin C, although not for starch, vitamin D, vitamin E and thiamine. The USNDB will allow the analysis of the dietary exposure of up to 150 food components in relation to health and disease risk within the EPIC study.

## Figures and Tables

**Table 1:** List of the 28 nutrients included the European prospective investigation into cancer and nutrition (EPIC) nutrient database (ENDB).

energy water total fat total saturated fatty acids total monounsaturated fatty acids total polyunsaturated fatty acids cholesterol	proteins carbohydrates starch sugar dietary fibre alcohol calcium	iron potassium magnesium Phosphorus vitamin D vitamin E retinol	beta-carotene vitamin B1 vitamin B2 vitamin B6 vitamin B12 vitamin C folate (2010)

**Table 2:** Mean, standard deviation and median of dietary intakes of 28 nutrients estimated by the U.S. nutrient database (USNDB) and the EPIC nutrient database (ENDB) and their absolute mean difference in nutrient intake, reported for the 24-hour dietary recall data (24-HDR) and the dietary questionnaire data (DQ).

		24-HDR (*N* = 34,064)				DQ (*N* = 476,768)			
Food Component	Database	Mean	Standard Deviation	Median	Mean Difference *	Mean	Standard Deviation	Median	Mean Difference *
Energy (kcal/day)	ENDB	2611.4	±905.2	2004		2071.4	±617.3	1993.8	
	USNDB	2596.4	±900.3	2023	20.2	2132.6	±623.5	2057.4	61.2
Water (g/day)	ENDB	2115.9	±789.2	2485		2259.7	±899.3	2171.7	
	USNDB	2136.0	±790.5	2471	−15.0	2248.4	±890.9	2157.3	−11.3
Total fats (g/day)	ENDB	85.1	±42.7	77.7		80.1	±29.3	76.1	
	USNDB	84.9	±41.9	77.6	−0.2	81.3	±29.6	77.5	1.2
Fatty acids, total	ENDB	33.7	±18.8	30.3		31.3	±13.0	29.3	
saturated (g/day)	USNDB	30.0	±16.8	26.8	−3.7	28.6	±12.2	26.7	−2.7
Fatty acids, total monounsaturated	ENDB	31.0	±17.8	27.4		28.7	±12.2	26.6	
(g/day)	USNDB	32.5	±18.0	29.0	1.5	30.4	±12.2	28.5	1.7
Fatty acids, total polyunsaturated	ENDB	13.3	±9.3	10.9		13.4	±6.0	12.2	
(g/day)	USNDB	15.6	±10.2	13.1	2.3	15.4	±6.5	14.2	2.0
Cholesterol	ENDB	326.2	±229.8	269.1		321.0	±150.5	298.1	
(mg/day)	USNDB	283.0	±200.8	234.4	−43.2	283.6	±133.4	264.7	−37.5
Total proteins	ENDB	84.4	±34.6	79.0		86.9	±27.4	83.9	
(g/day)	USNDB	78.3	±30.8	73.8	−6.1	82.6	±25.5	79.7	−4.3
Carbohydrates	ENDB	227.2	±89.4	214.5		229.7	±74.5	220.0	
(g/day)	USNDB	244.6	±97.2	230.4	17.4	253.7	±80.6	243.4	24.0
Sugar, total	ENDB	104.6	±54.1	95.3		104.2	±44.1	97.3	
(g/day)	USNDB	98.7	±54.0	89.2	−5.9	102.1	±46.1	94.3	−2.0
Starch (g/day)	ENDB	118.7	±57.9	109.2		122.0	±49.0	114.4	
	USNDB	41.9	±40.0	32.3	−76.8	49.6	±32.4	42.7	−72.3
Dietary fiber,	ENDB	21.2	±9.9	19.6		22.8	±7.8	21.8	
total (g/day)	USNDB	21.0	±10.4	19.2	−0.2	23.8	±8.6	22.6	1.0
Alcohol (g/day)	ENDB	14.8	±24.4	1.8		12.0	±16.9	5.8	
	USNDB	15.9	±26.1	1.3	1.0	12.9	±18.1	6.3	0.9
Calcium, Ca	ENDB	943.3	±455.7	870.9		995.2	±411.1	935.9	
(mg/day)	USNDB	995.0	±489.8	910.8	51.7	1079.4	±447.9	1012.6	84.1
Iron, Fe (mg/day)	ENDB	12.4	±6.0	11.4		12.9	±4.2	12.4	
	USNDB	12.2	±5.8	11.1	−0.3	13.0	±4.7	12.3	0.2
Potassium, K	ENDB	3516.3	±1256.6	3361.4		3659.7	±1030.9	3554.9	
(mg/day)	USNDB	3071.6	±1101.2	2952.8	−444.7	3282.5	±949.4	3182.4	−377.2
Magnesium, Mg	ENDB	355.4	±128.8	336.8		360.4	±111.4	345.6	
(mg/day)	USNDB	325.7	±127.9	304.9	−29.7	347.2	±103.6	334.3	−13.2
Phosphorus, P	ENDB	1405.9	±530.4	1333.6		1492.1	±456.3	1439.0	
(mg/day)	USNDB	1356.7	±525.5	1282.8	−49.2	1447.2	±446.1	1395.1	−44.8
Vitamin D	ENDB	4.3	±6.4	2.4		4.3	±3.5	3.4	
(µg/day)	USNDB	3.1	±3.9	1.9	−1.3	3.4	±3.3	2.6	−0.9
Vitamin E (alpha-tocopherol)	ENDB	11.3	±8.1	9.3		11.7	±5.3	10.6	
(mg/day)	USNDB	9.3	±5.9	8.0	−2.1	9.8	±4.5	9.0	−1.9
Retinol (µg/day)	ENDB	859.5	±1819.0	444.5		845.7	±750.0	640.9	
	USNDB	743.8	±1302.5	466.4	−115.7	746.7	±587.6	603.9	−99.0
Beta-carotene	ENDB	2852.2	±3845.7	1555.0		3506.5	±2773.6	2802.4	
(µg/day)	USNDB	2964.8	±4219.4	1452.5	112.6	3961.3	±3006.2	3250.1	454.8
Thiamin, B1	ENDB	1.2	±0.6	1.1		1.3	±0.5	1.3	
(mg/day)	USNDB	1.7	±1.0	1.5	0.5	1.8	±0.7	1.7	0.5
Riboflavin, B2	ENDB	1.7	±0.8	1.6		1.9	±0.8	1.7	
(mg/day)	USNDB	2.2	±0.9	2.1	0.6	2.3	±0.8	2.2	0.4
Cobalamin, B12	ENDB	6.4	±9.8	4.3		6.6	±4.1	5.8	
(µg/day)	USNDB	6.6	±8.8	4.7	0.2	7.0	±4.0	6.2	0.3
Vitamin B6	ENDB	1.7	±0.8	1.6		1.9	±0.6	1.8	
(µg/day)	USNDB	1.9	±0.9	1.7	0.1	2.0	±0.7	1.9	0.2
Vitamin C	ENDB	112.0	±89.6	90.0		122.4	±63.8	110.6	
(mg/day)	USNDB	103.7	±94.6	76.6	−8.2	116.3	±66.9	102.8	−6.0
Folate, food	ENDB	264.6	±137.0	137.0		305.0	±120.0	284.7	
(µg/day)	USNDB	328.4	±156.9	141.7	11.8	304.9	±110.7	290.1	−0.2

* Absolute differences in mean nutrient intake between the USNDB and ENDB were statistically significant (paired samples *t*-test: *p* < 0.001) for all nutrients.

**Table 3:** Pearson correlation coefficients and weighted kappas (κ) for dietary intakes of 28 nutrients estimated by the U.S. nutrient database (USNDB) and the EPIC nutrient database (ENDB), reported for the 24-h dietary recall data (24-HDR) and the dietary questionnaire data (DQ).

	24-HDR (*N* = 34,064)		DQ (*N* = 476,768)	
	Pearson Correlation Coefficient *	Weighted κ	Pearson Correlation Coefficient *	Weighted κ
Energy (kcal/day)	0.96	0.83	0.98	0.89
Water (g/day)	1.00	0.96	1.00	0.98
Total fats (g/day)	0.94	0.80	0.97	0.86
Fatty acids, total saturated (g/day)	0.89	0.73	0.93	0.78
Fatty acids, total monounsaturated (g/day)	0.91	0.73	0.95	0.80
Fatty acids, total polyunsaturated (g/day)	0.83	0.65	0.88	0.69
Cholesterol (mg/day)	0.87	0.72	0.91	0.75
Total proteins (g/day)	0.93	0.76	0.97	0.84
Carbohydrates (g/day)	0.92	0.78	0.95	0.83
Sugar, total (g/day)	0.91	0.77	0.93	0.82
Starch (g/day)	0.54	0.30	0.72	0.43
Dietary fiber, total (g/day)	0.85	0.69	0.93	0.77
Alcohol (g/day)	0.98	0.82	0.99	0.96
Calcium, Ca (mg/day)	0.87	0.70	0.90	0.72
Iron, Fe (mg/day)	0.71	0.56	0.84	0.66
Potassium, K (mg/day)	0.91	0.75	0.97	0.84
Magnesium, Mg (mg/day)	0.76	0.59	0.78	0.63
Phosphorus, P (mg/day)	0.89	0.73	0.92	0.77
Vitamin D (µg/day)	0.48	0.41	0.83	0.49
Vitamin E (alpha-tocopherol) (mg/day)	0.70	0.53	0.62	0.50
Retinol (µg/day)	0.74	0.74	0.81	0.74
Beta-carotene (µg/day)	0.90	0.69	0.90	0.72
Thiamin, B1 (mg/day)	0.56	0.54	0.78	0.60
Riboflavin, B2 (mg/day)	0.81	0.57	0.88	0.66
Cobalamin, B12 (µg/day)	0.87	0.61	0.90	0.69
Vitamin B6 (mg/day)	0.79	0.64	0.86	0.72
Vitamin C (mg/day)	0.93	0.78	0.97	0.85
Folate, food (µg/day)	0.78	0.63	0.88	0.72

* Pearson correlation coefficients for the 28 nutrient intakes measured by the USNDB and ENDB were significant at the level of *p* < 0.001 for all nutrients.

## Data Availability

EPIC data and biospecimens are available for investigators who seek to answer important questions on health and disease in the context of research projects that are consistent with the legal and ethical standard practices of IARC/WHO and the EPIC Centres. The primary responsibility for accessing the data, including the nutrient databases (ENDB and USNDB) obtained in the frame of the present publication, belongs to the EPIC centres that provided them. The use of a random sample of anonymised data from the EPIC study can be requested by contacting epic@iarc.fr. The request will then be passed to members of the EPIC Steering Committee for deliberation.

## Supplementary Materials

The following are available online at https://www.mdpi.com/2072-6643/12/10/2906/s1: Table S1: Mean, standard deviation and median of dietary intakes of 28 nutrients of the U.S. nutrient database (USNDB) and the EPIC nutrient database (ENDB) and their absolute mean difference in nutrient intake, reported for the 24-h dietary recall data (24-HDR) and the dietary questionnaire data (DQ) by country. Table S2: Pearson correlation coefficients and weighted kappas (κ) for dietary intakes of 28 nutrients of the U.S. nutrient database (USNDB) and the EPIC nutrient database (ENDB), reported for the 24-h dietary recall data (24-HDR) and the dietary questionnaire data (DQ) by country. Table S3: Short overview of the U.S. nutrient database (USNDB) and the EPIC nutrient database (ENDB) reference component-specific definition and standard analytical methods and approaches used. Figure S1: Bland-Altman plots for 24-h dietary recall (24-HDR) data for 23 nutrients intakes between the U.S. nutrient database (USNDB) and the EPIC nutrient database (ENDB). The mean difference is represented by the full line, the upper and lower limit of agreement are presented by dotted lines. Figure S2: Bland-Altman plots for dietary questionnaire (DQ) data for 23 nutrients intakes between the U.S. nutrient database (USNDB) and the EPIC nutrient database (ENDB). The mean difference is represented by the full line, the upper and lower limit of agreement are presented by dotted lines.
