# Supplementary material for: Comparing Calculated Nutrient Intakes Using Different Food Composition Databases: Results from the European Prospective Investigation into Cancer and Nutrition (EPIC) Cohort
Source: Nutrients. 2020 Sep 23;12(10):2906. doi: 10.3390/nu12102906 (PMC7650652; doi:10.3390/nu12102906)
Supplement: Supplementary file 1 [file nutrients-12-02906-s001.zip › Revision2_Nutrients_FigureS1.pptx]

## Slide 1
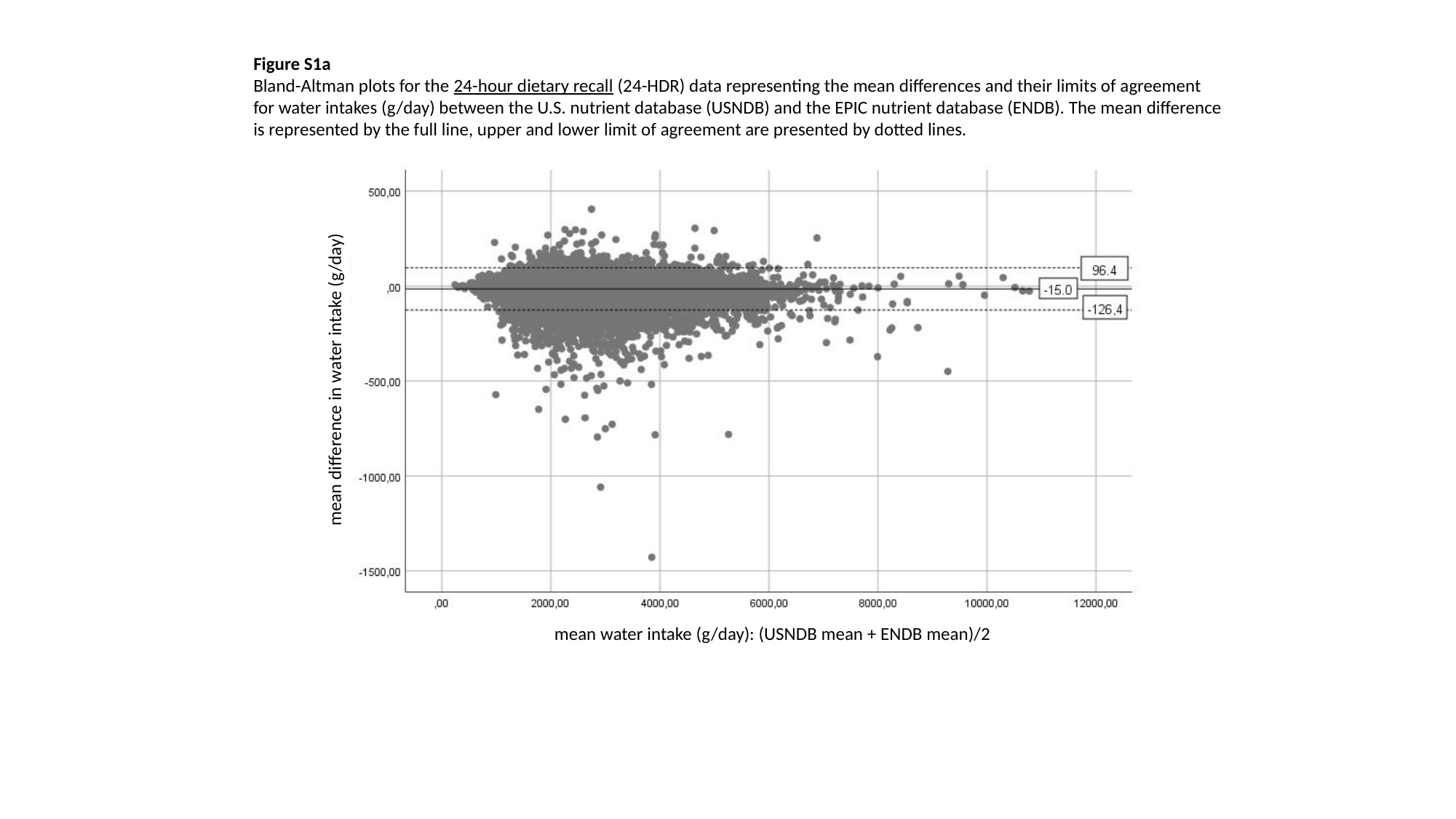

Figure S1a
Bland-Altman plots for the 24-hour dietary recall (24-HDR) data representing the mean differences and their limits of agreement for water intakes (g/day) between the U.S. nutrient database (USNDB) and the EPIC nutrient database (ENDB). The mean difference is represented by the full line, upper and lower limit of agreement are presented by dotted lines.
mean difference in water intake (g/day)
mean water intake (g/day): (USNDB mean + ENDB mean)/2

## Slide 2
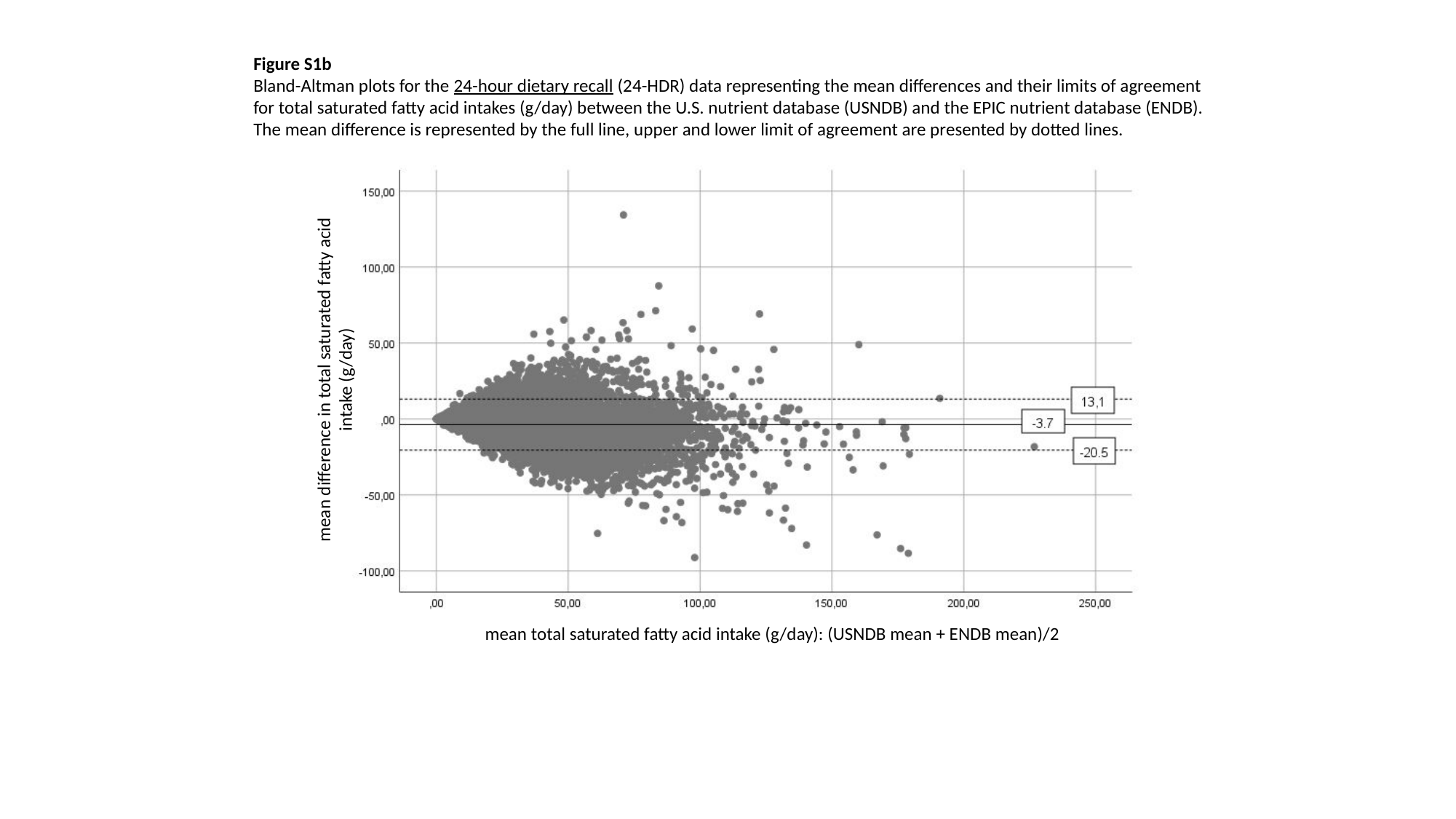

Figure S1b
Bland-Altman plots for the 24-hour dietary recall (24-HDR) data representing the mean differences and their limits of agreement for total saturated fatty acid intakes (g/day) between the U.S. nutrient database (USNDB) and the EPIC nutrient database (ENDB). The mean difference is represented by the full line, upper and lower limit of agreement are presented by dotted lines.
mean difference in total saturated fatty acid intake (g/day)
mean total saturated fatty acid intake (g/day): (USNDB mean + ENDB mean)/2

## Slide 3
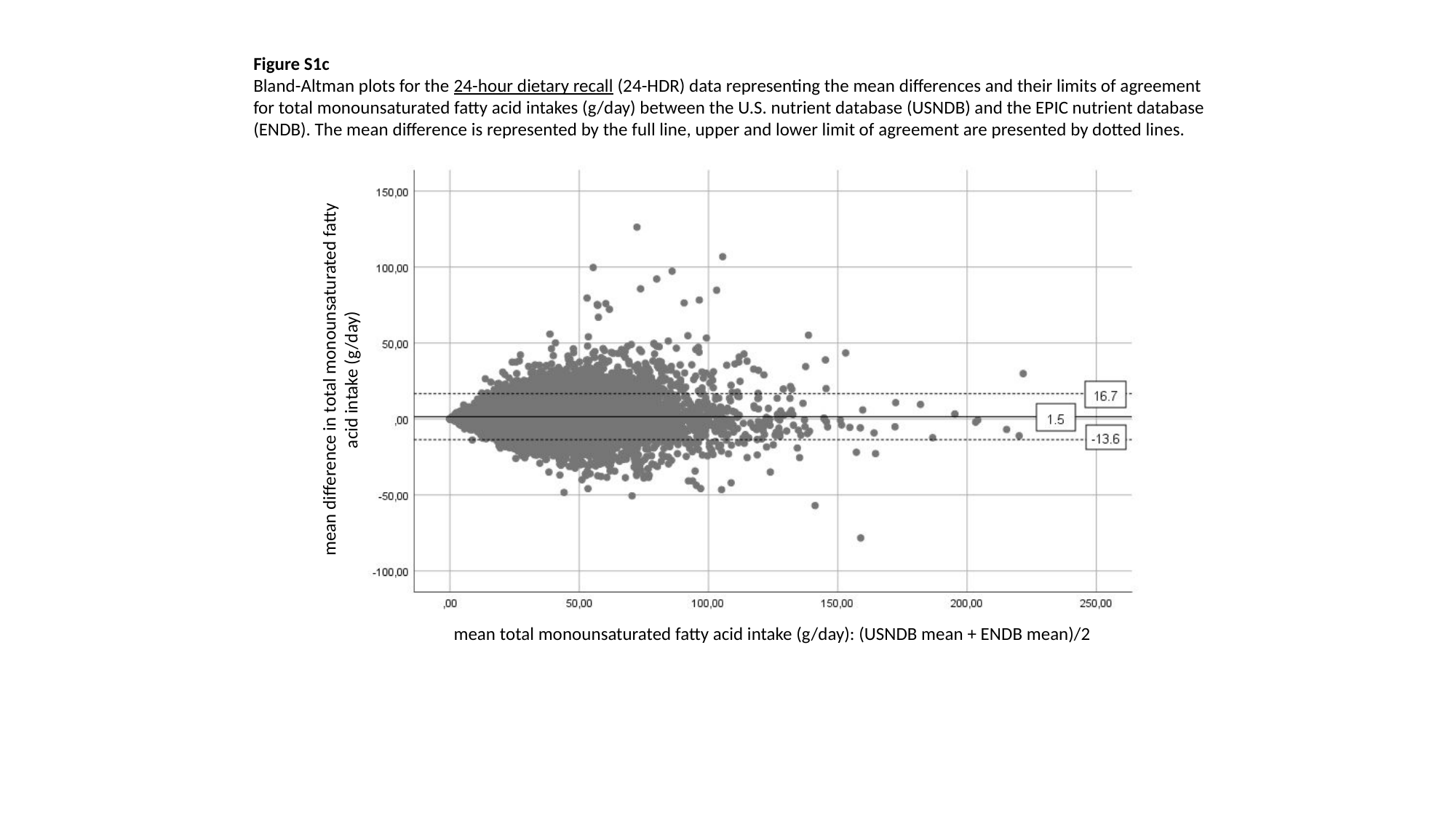

Figure S1c
Bland-Altman plots for the 24-hour dietary recall (24-HDR) data representing the mean differences and their limits of agreement for total monounsaturated fatty acid intakes (g/day) between the U.S. nutrient database (USNDB) and the EPIC nutrient database (ENDB). The mean difference is represented by the full line, upper and lower limit of agreement are presented by dotted lines.
mean difference in total monounsaturated fatty acid intake (g/day)
mean total monounsaturated fatty acid intake (g/day): (USNDB mean + ENDB mean)/2

## Slide 4
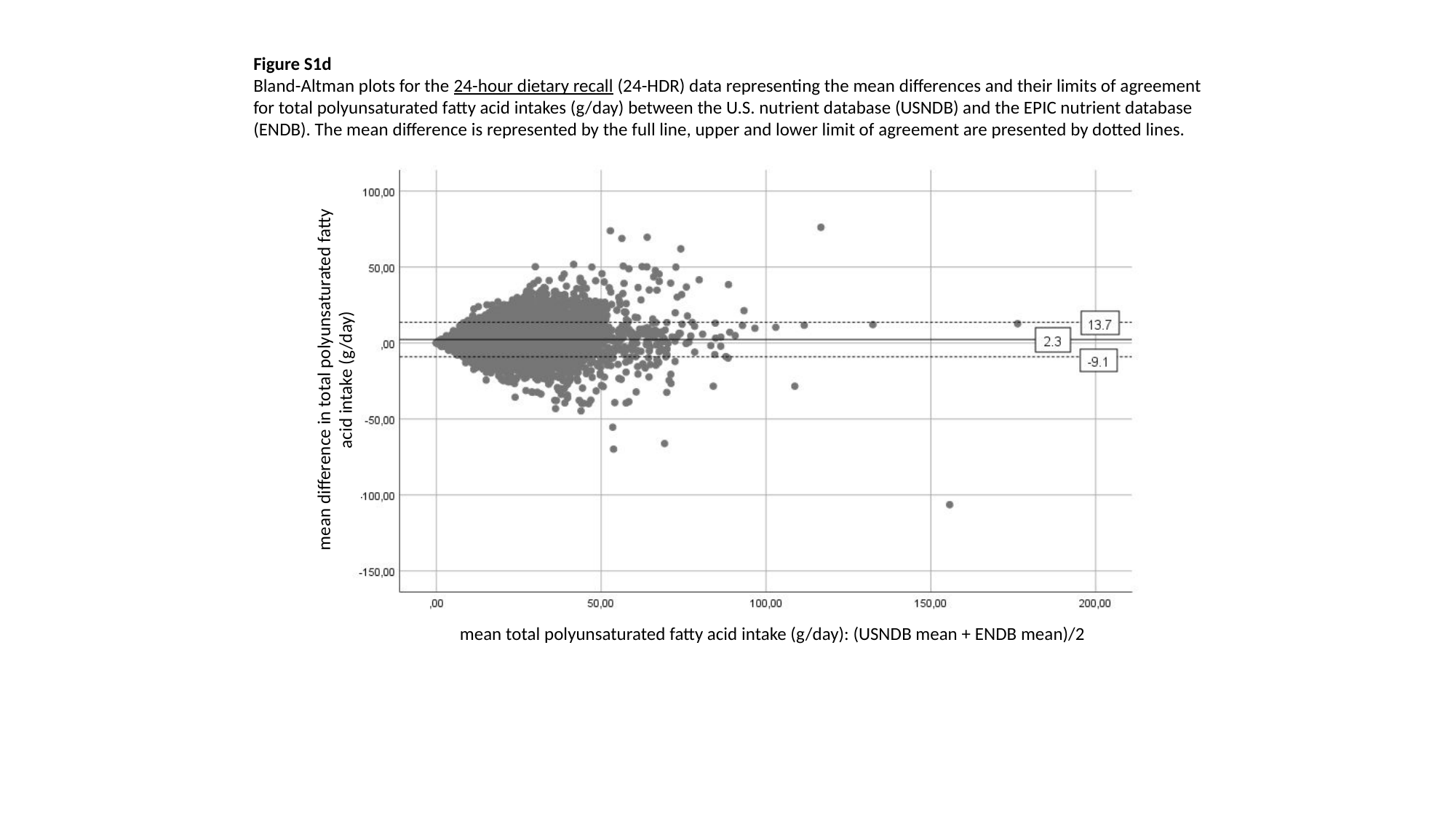

Figure S1d
Bland-Altman plots for the 24-hour dietary recall (24-HDR) data representing the mean differences and their limits of agreement for total polyunsaturated fatty acid intakes (g/day) between the U.S. nutrient database (USNDB) and the EPIC nutrient database (ENDB). The mean difference is represented by the full line, upper and lower limit of agreement are presented by dotted lines.
mean difference in total polyunsaturated fatty acid intake (g/day)
mean total polyunsaturated fatty acid intake (g/day): (USNDB mean + ENDB mean)/2

## Slide 5
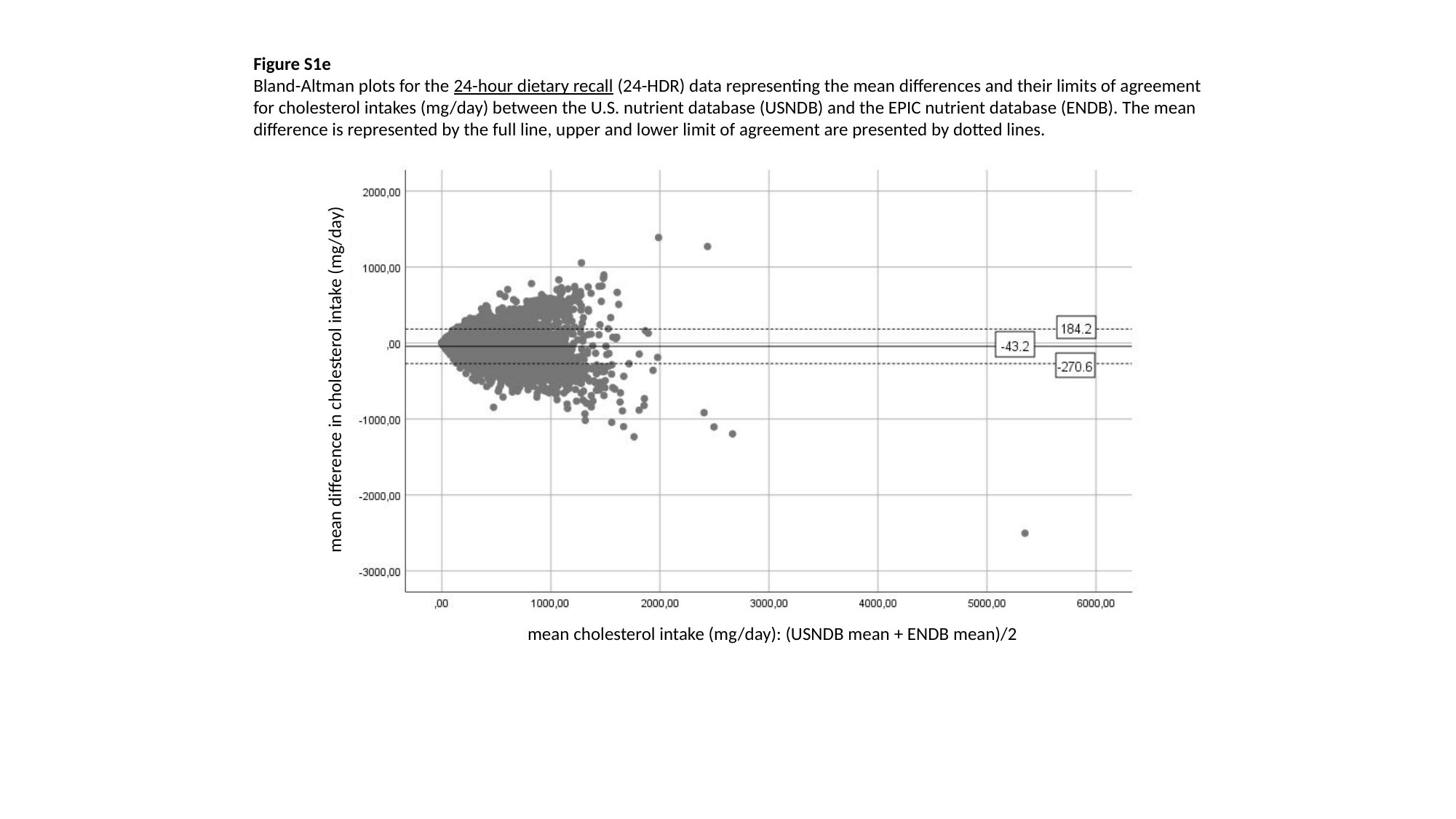

Figure S1e
Bland-Altman plots for the 24-hour dietary recall (24-HDR) data representing the mean differences and their limits of agreement for cholesterol intakes (mg/day) between the U.S. nutrient database (USNDB) and the EPIC nutrient database (ENDB). The mean difference is represented by the full line, upper and lower limit of agreement are presented by dotted lines.
mean difference in cholesterol intake (mg/day)
mean cholesterol intake (mg/day): (USNDB mean + ENDB mean)/2

## Slide 6
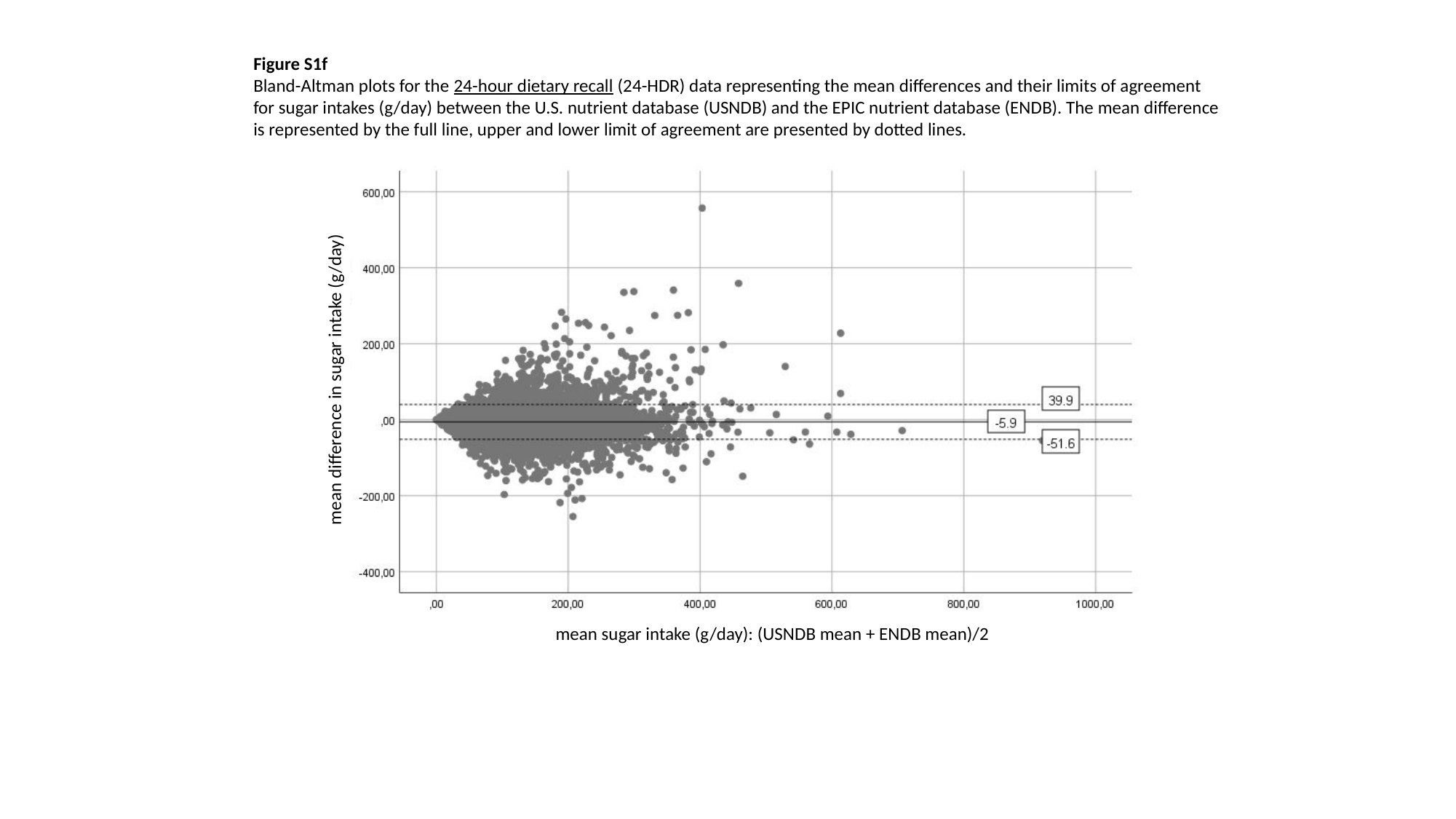

Figure S1f
Bland-Altman plots for the 24-hour dietary recall (24-HDR) data representing the mean differences and their limits of agreement for sugar intakes (g/day) between the U.S. nutrient database (USNDB) and the EPIC nutrient database (ENDB). The mean difference is represented by the full line, upper and lower limit of agreement are presented by dotted lines.
mean difference in sugar intake (g/day)
mean sugar intake (g/day): (USNDB mean + ENDB mean)/2

## Slide 7
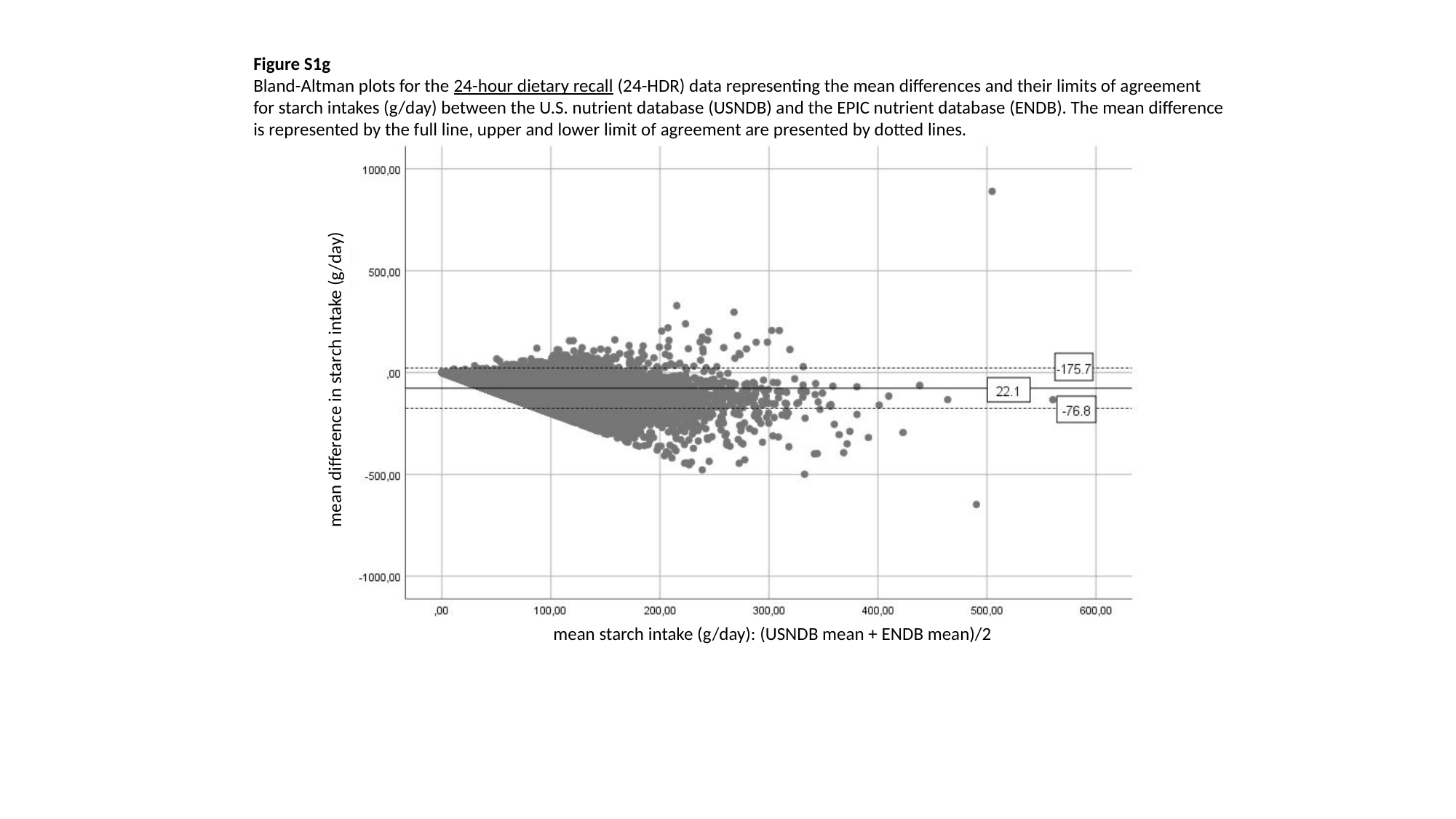

Figure S1g
Bland-Altman plots for the 24-hour dietary recall (24-HDR) data representing the mean differences and their limits of agreement for starch intakes (g/day) between the U.S. nutrient database (USNDB) and the EPIC nutrient database (ENDB). The mean difference is represented by the full line, upper and lower limit of agreement are presented by dotted lines.
mean difference in starch intake (g/day)
mean starch intake (g/day): (USNDB mean + ENDB mean)/2

## Slide 8
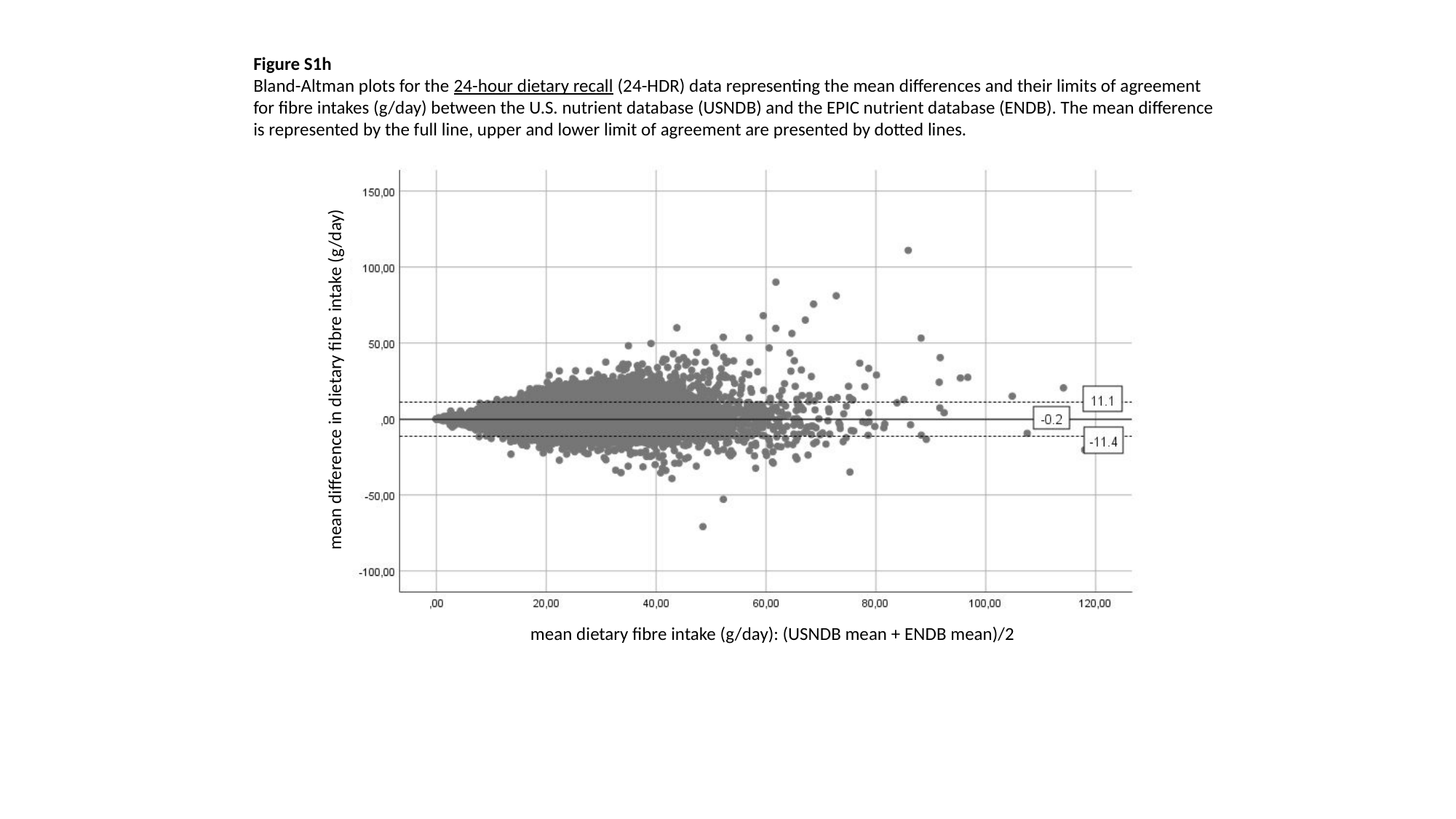

Figure S1h
Bland-Altman plots for the 24-hour dietary recall (24-HDR) data representing the mean differences and their limits of agreement for fibre intakes (g/day) between the U.S. nutrient database (USNDB) and the EPIC nutrient database (ENDB). The mean difference is represented by the full line, upper and lower limit of agreement are presented by dotted lines.
mean difference in dietary fibre intake (g/day)
mean dietary fibre intake (g/day): (USNDB mean + ENDB mean)/2

## Slide 9
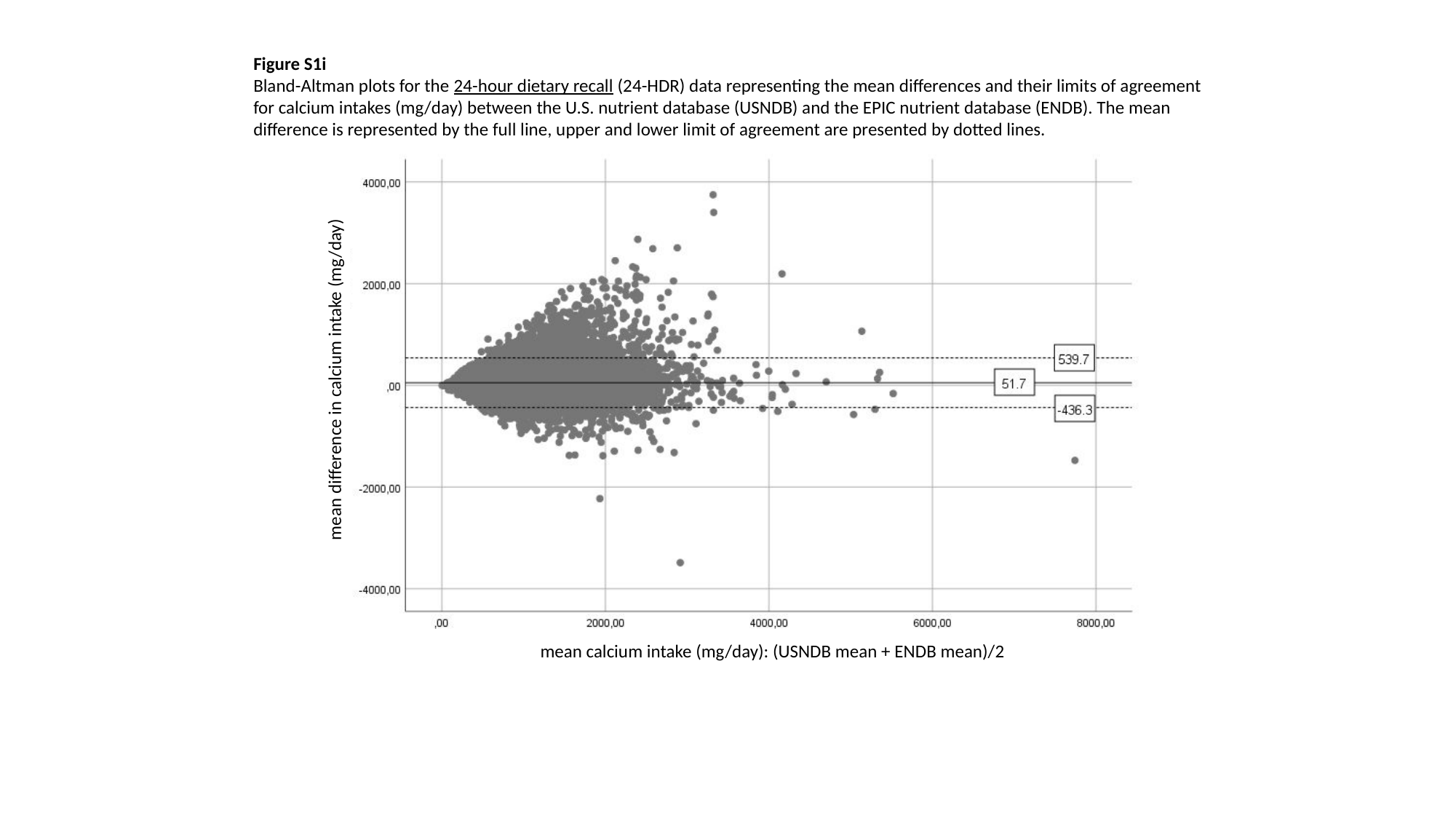

Figure S1i
Bland-Altman plots for the 24-hour dietary recall (24-HDR) data representing the mean differences and their limits of agreement for calcium intakes (mg/day) between the U.S. nutrient database (USNDB) and the EPIC nutrient database (ENDB). The mean difference is represented by the full line, upper and lower limit of agreement are presented by dotted lines.
mean difference in calcium intake (mg/day)
mean calcium intake (mg/day): (USNDB mean + ENDB mean)/2

## Slide 10
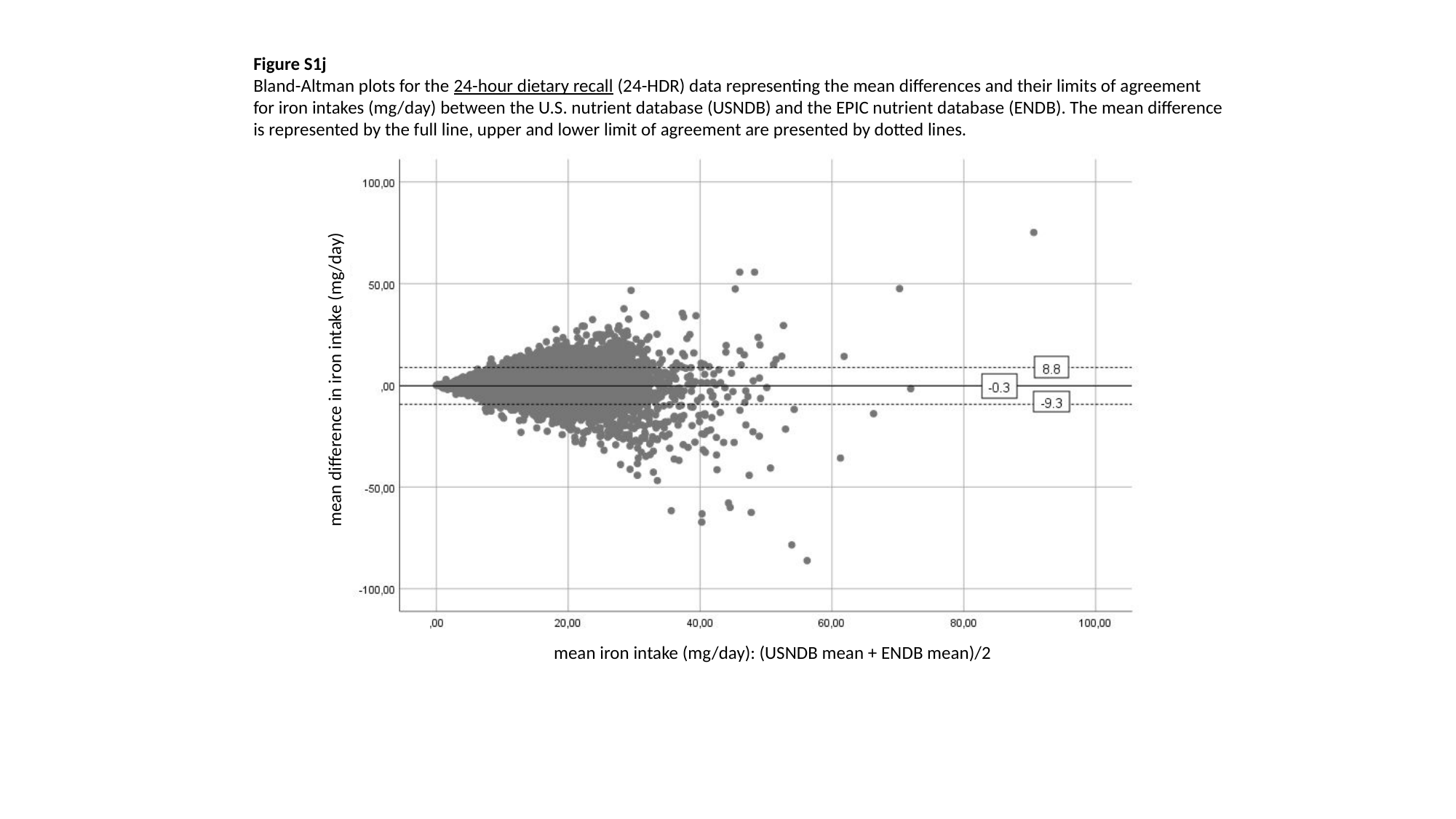

Figure S1j
Bland-Altman plots for the 24-hour dietary recall (24-HDR) data representing the mean differences and their limits of agreement for iron intakes (mg/day) between the U.S. nutrient database (USNDB) and the EPIC nutrient database (ENDB). The mean difference is represented by the full line, upper and lower limit of agreement are presented by dotted lines.
mean difference in iron intake (mg/day)
mean iron intake (mg/day): (USNDB mean + ENDB mean)/2

## Slide 11
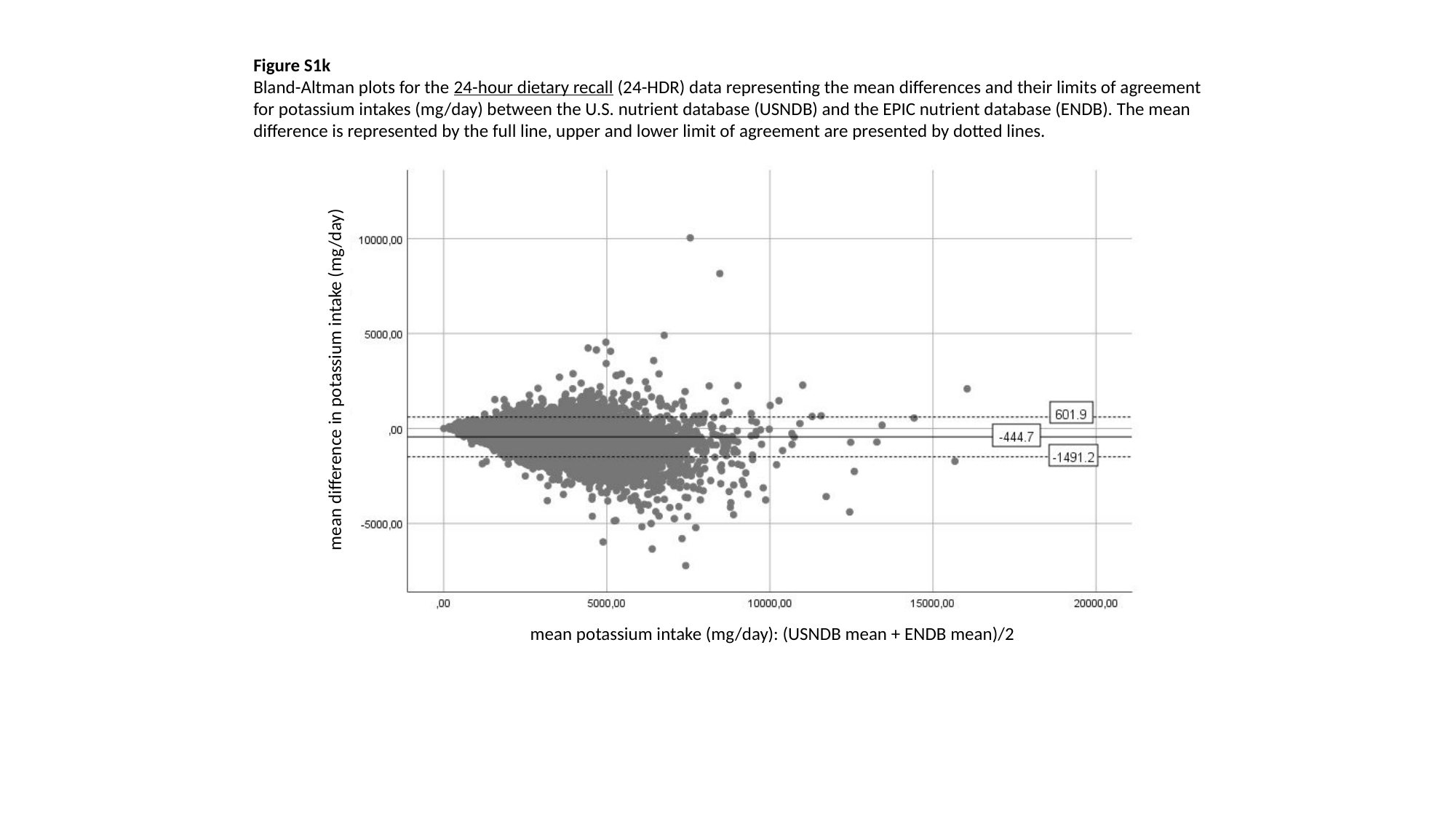

Figure S1k
Bland-Altman plots for the 24-hour dietary recall (24-HDR) data representing the mean differences and their limits of agreement for potassium intakes (mg/day) between the U.S. nutrient database (USNDB) and the EPIC nutrient database (ENDB). The mean difference is represented by the full line, upper and lower limit of agreement are presented by dotted lines.
mean difference in potassium intake (mg/day)
mean potassium intake (mg/day): (USNDB mean + ENDB mean)/2

## Slide 12
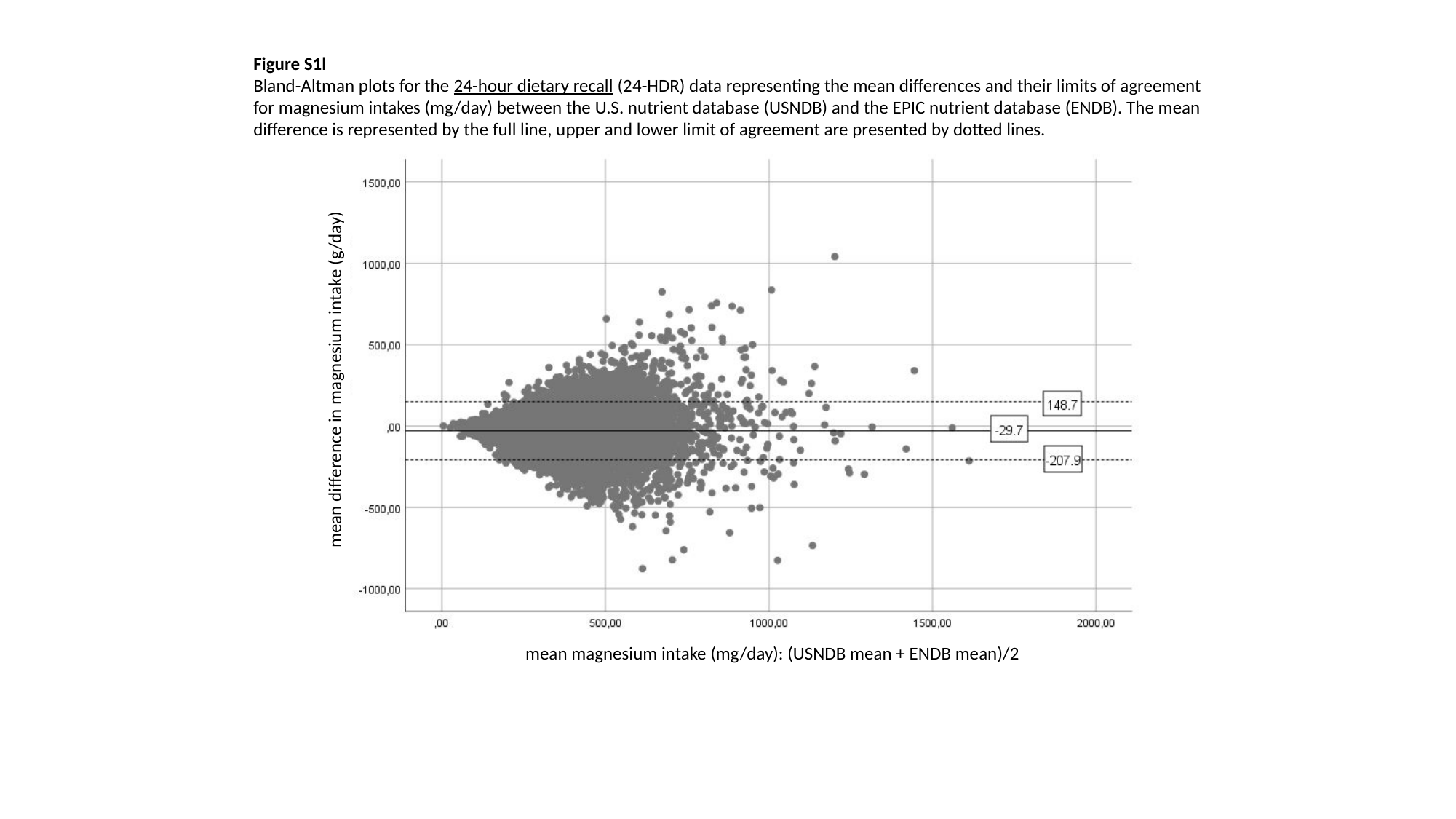

Figure S1l
Bland-Altman plots for the 24-hour dietary recall (24-HDR) data representing the mean differences and their limits of agreement for magnesium intakes (mg/day) between the U.S. nutrient database (USNDB) and the EPIC nutrient database (ENDB). The mean difference is represented by the full line, upper and lower limit of agreement are presented by dotted lines.
mean difference in magnesium intake (g/day)
mean magnesium intake (mg/day): (USNDB mean + ENDB mean)/2

## Slide 13
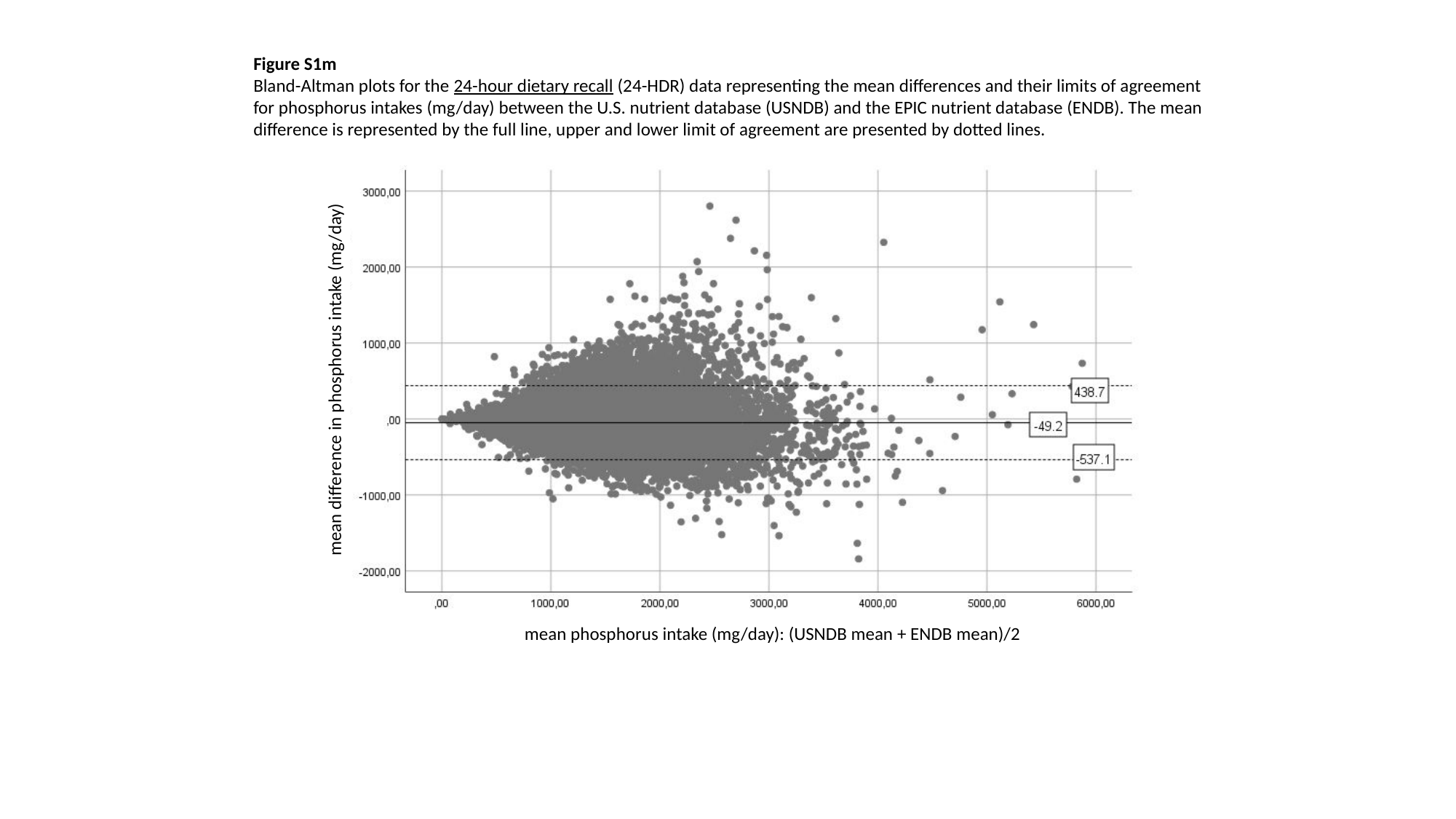

Figure S1m
Bland-Altman plots for the 24-hour dietary recall (24-HDR) data representing the mean differences and their limits of agreement for phosphorus intakes (mg/day) between the U.S. nutrient database (USNDB) and the EPIC nutrient database (ENDB). The mean difference is represented by the full line, upper and lower limit of agreement are presented by dotted lines.
mean difference in phosphorus intake (mg/day)
mean phosphorus intake (mg/day): (USNDB mean + ENDB mean)/2

## Slide 14
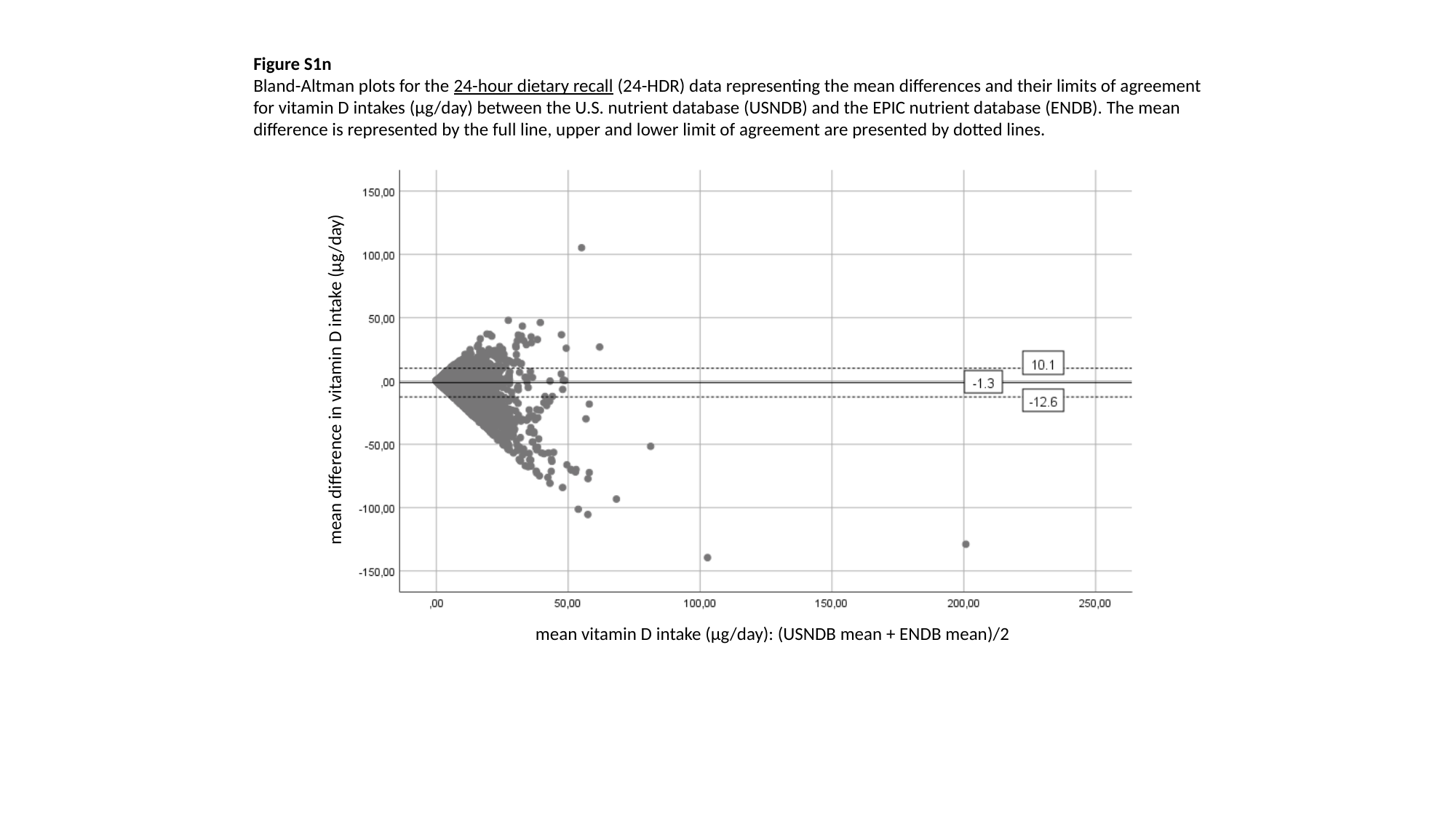

Figure S1n
Bland-Altman plots for the 24-hour dietary recall (24-HDR) data representing the mean differences and their limits of agreement for vitamin D intakes (µg/day) between the U.S. nutrient database (USNDB) and the EPIC nutrient database (ENDB). The mean difference is represented by the full line, upper and lower limit of agreement are presented by dotted lines.
mean difference in vitamin D intake (µg/day)
mean vitamin D intake (µg/day): (USNDB mean + ENDB mean)/2

## Slide 15
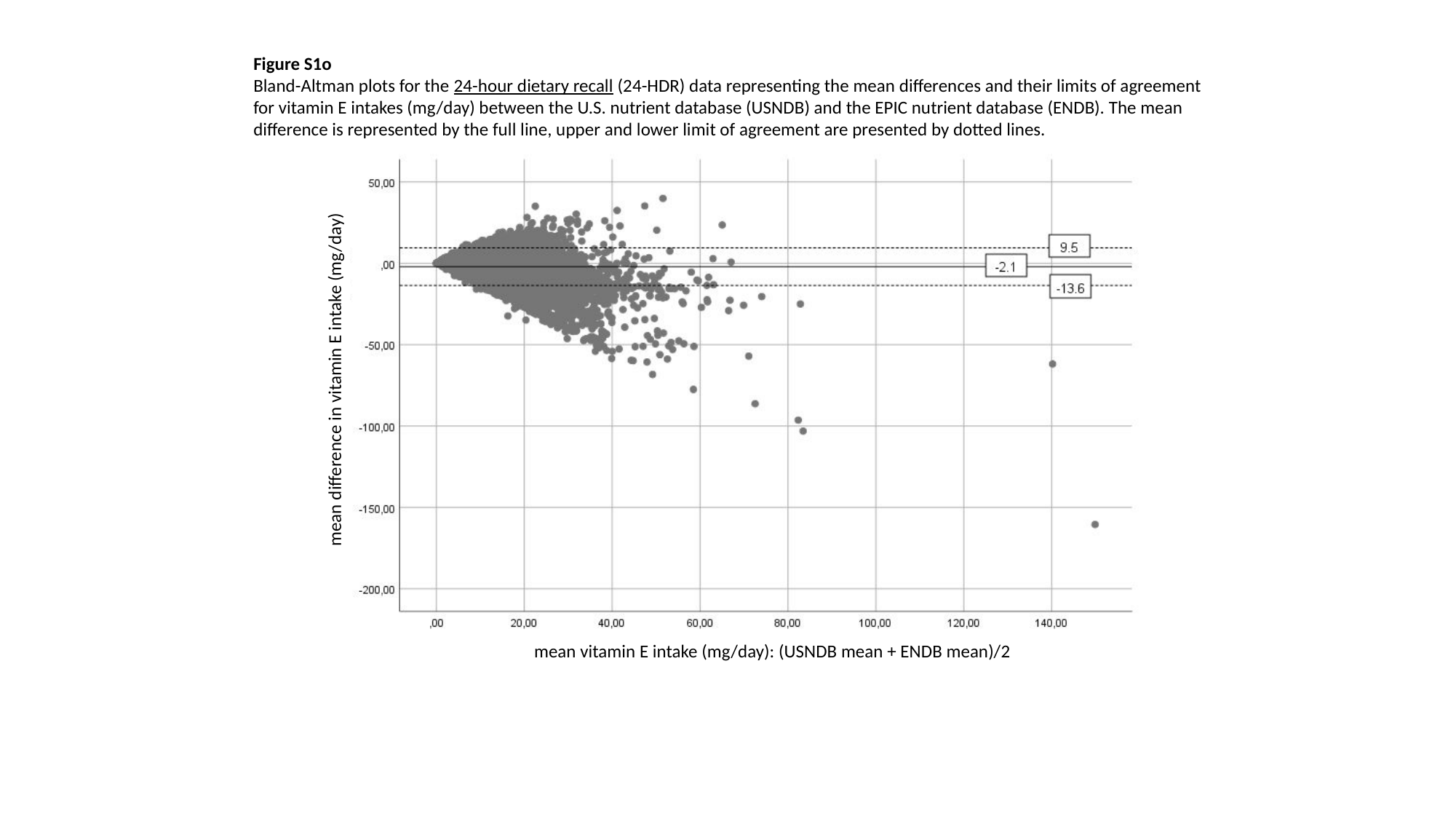

Figure S1o
Bland-Altman plots for the 24-hour dietary recall (24-HDR) data representing the mean differences and their limits of agreement for vitamin E intakes (mg/day) between the U.S. nutrient database (USNDB) and the EPIC nutrient database (ENDB). The mean difference is represented by the full line, upper and lower limit of agreement are presented by dotted lines.
mean difference in vitamin E intake (mg/day)
mean vitamin E intake (mg/day): (USNDB mean + ENDB mean)/2

## Slide 16
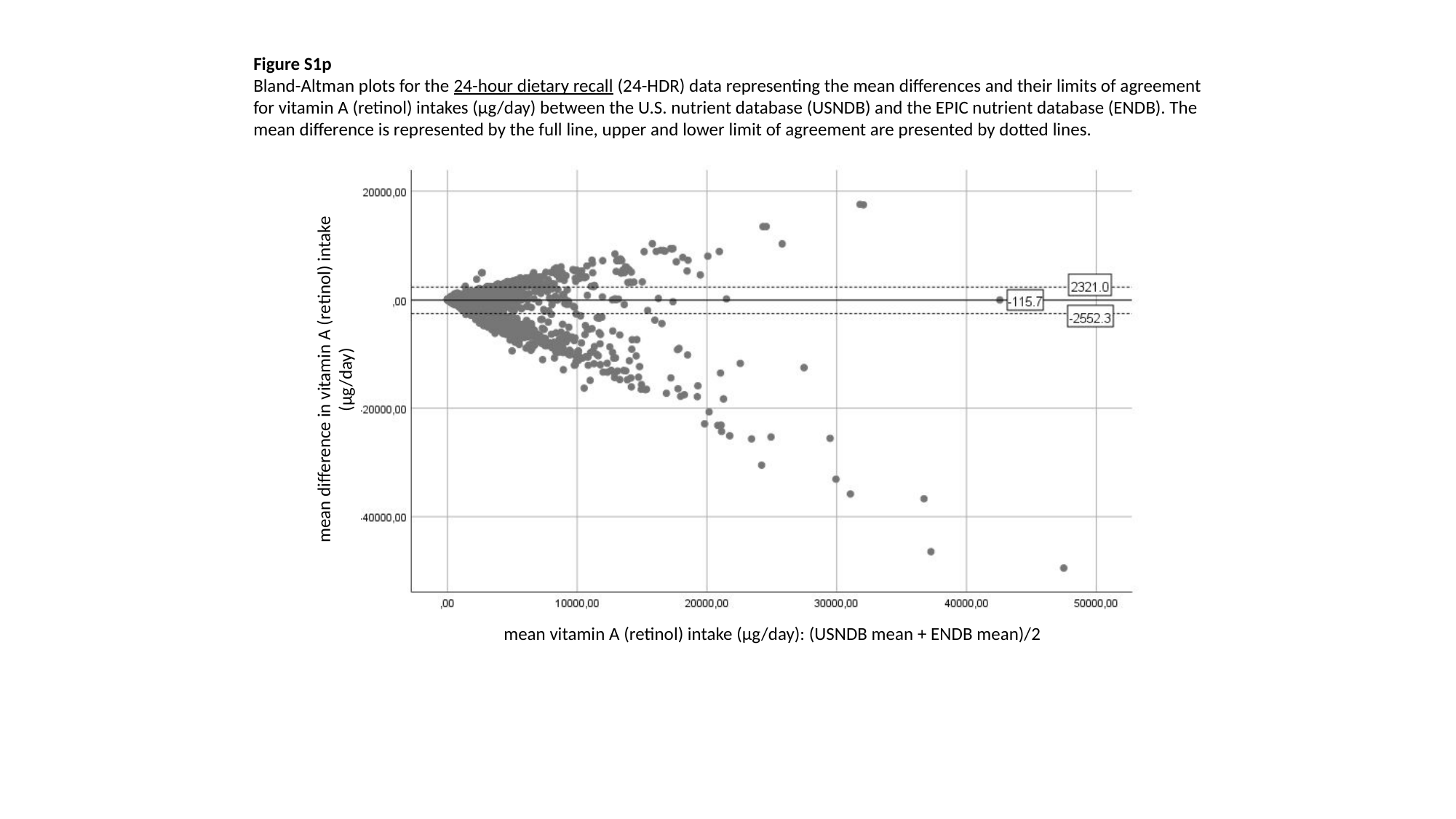

Figure S1p
Bland-Altman plots for the 24-hour dietary recall (24-HDR) data representing the mean differences and their limits of agreement for vitamin A (retinol) intakes (µg/day) between the U.S. nutrient database (USNDB) and the EPIC nutrient database (ENDB). The mean difference is represented by the full line, upper and lower limit of agreement are presented by dotted lines.
mean difference in vitamin A (retinol) intake (µg/day)
mean vitamin A (retinol) intake (µg/day): (USNDB mean + ENDB mean)/2

## Slide 17
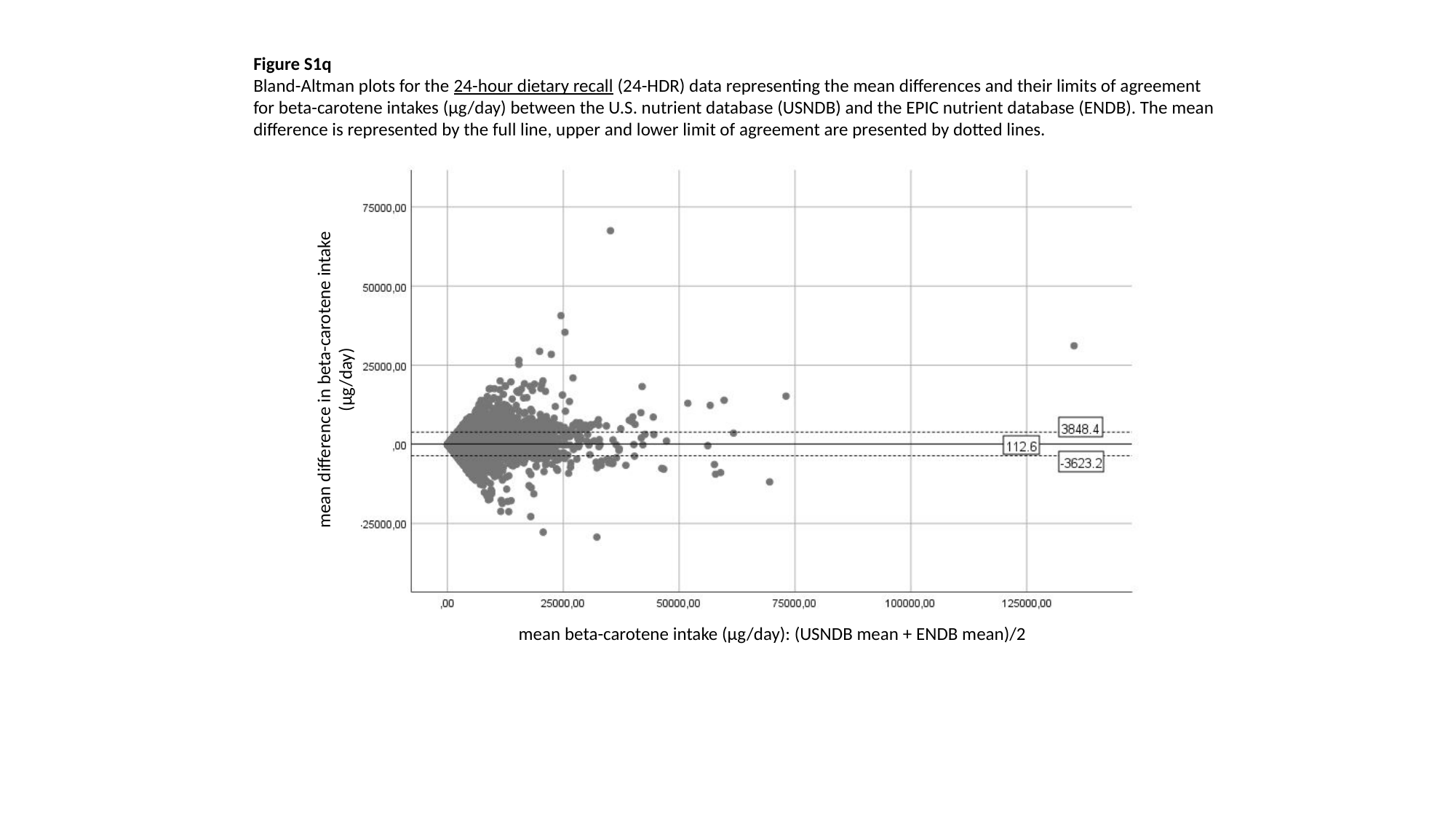

Figure S1q
Bland-Altman plots for the 24-hour dietary recall (24-HDR) data representing the mean differences and their limits of agreement for beta-carotene intakes (µg/day) between the U.S. nutrient database (USNDB) and the EPIC nutrient database (ENDB). The mean difference is represented by the full line, upper and lower limit of agreement are presented by dotted lines.
mean difference in beta-carotene intake (µg/day)
mean beta-carotene intake (µg/day): (USNDB mean + ENDB mean)/2

## Slide 18
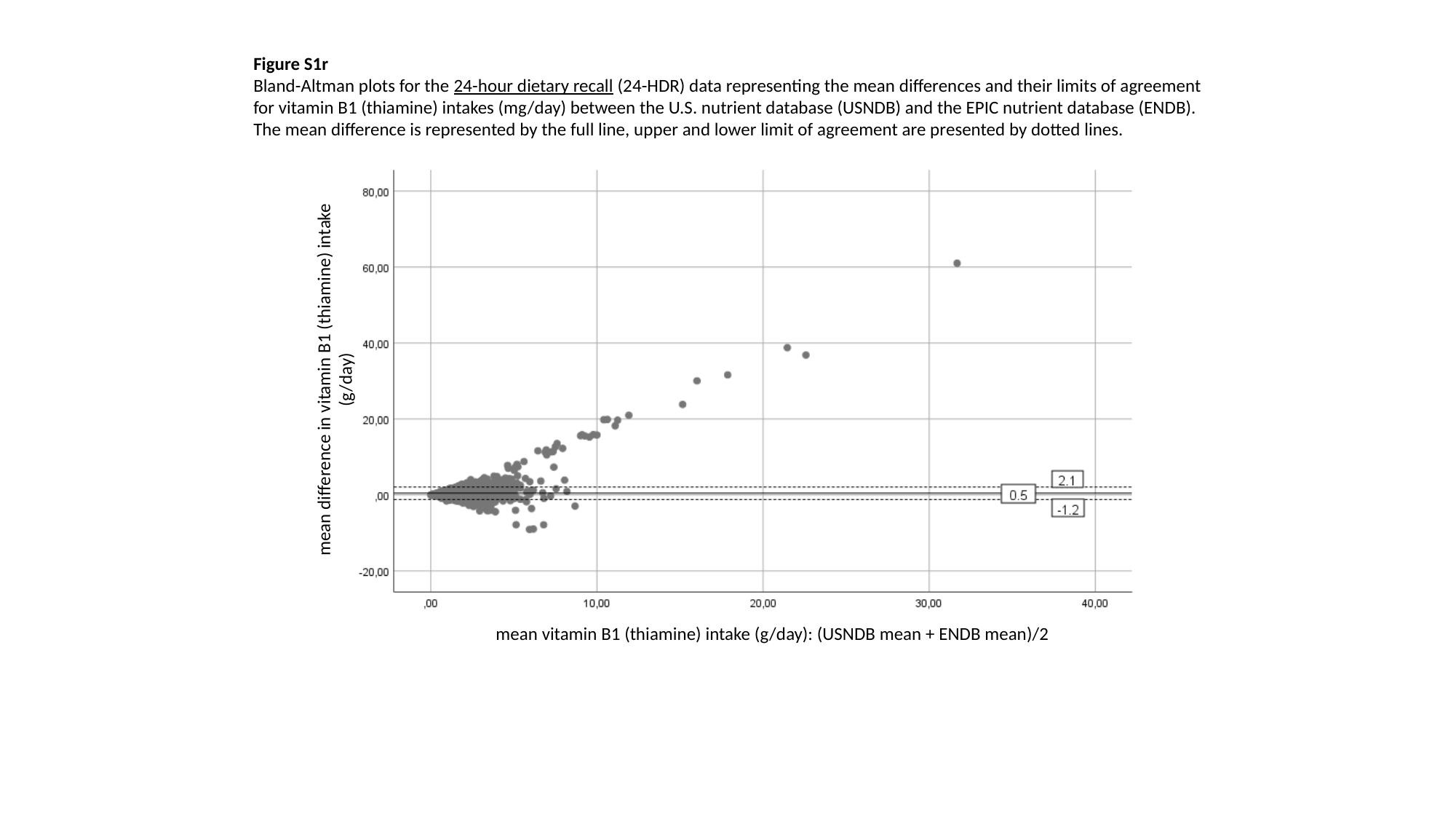

Figure S1r
Bland-Altman plots for the 24-hour dietary recall (24-HDR) data representing the mean differences and their limits of agreement for vitamin B1 (thiamine) intakes (mg/day) between the U.S. nutrient database (USNDB) and the EPIC nutrient database (ENDB). The mean difference is represented by the full line, upper and lower limit of agreement are presented by dotted lines.
mean difference in vitamin B1 (thiamine) intake (g/day)
mean vitamin B1 (thiamine) intake (g/day): (USNDB mean + ENDB mean)/2

## Slide 19
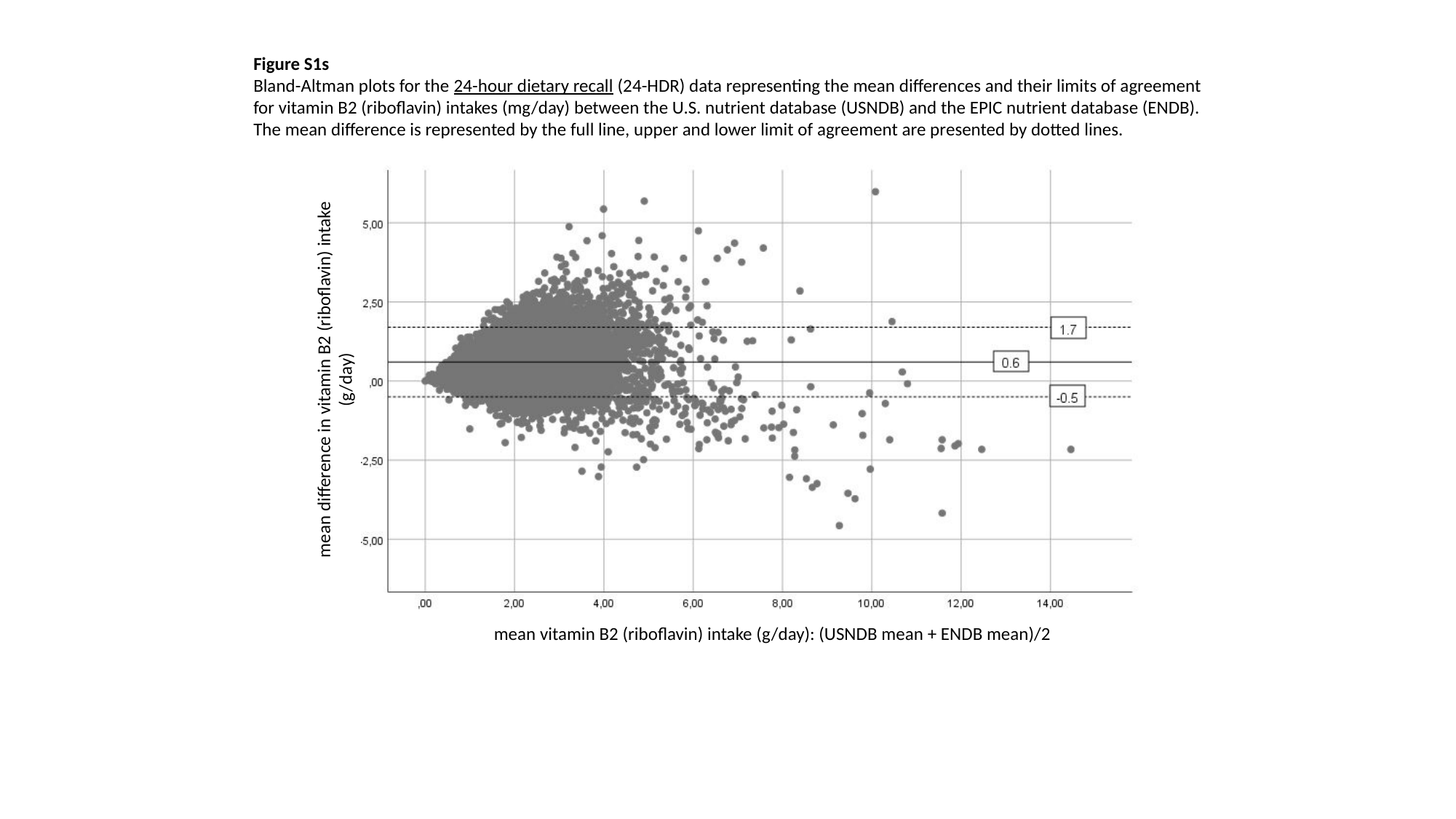

Figure S1s
Bland-Altman plots for the 24-hour dietary recall (24-HDR) data representing the mean differences and their limits of agreement for vitamin B2 (riboflavin) intakes (mg/day) between the U.S. nutrient database (USNDB) and the EPIC nutrient database (ENDB). The mean difference is represented by the full line, upper and lower limit of agreement are presented by dotted lines.
mean difference in vitamin B2 (riboflavin) intake (g/day)
mean vitamin B2 (riboflavin) intake (g/day): (USNDB mean + ENDB mean)/2

## Slide 20
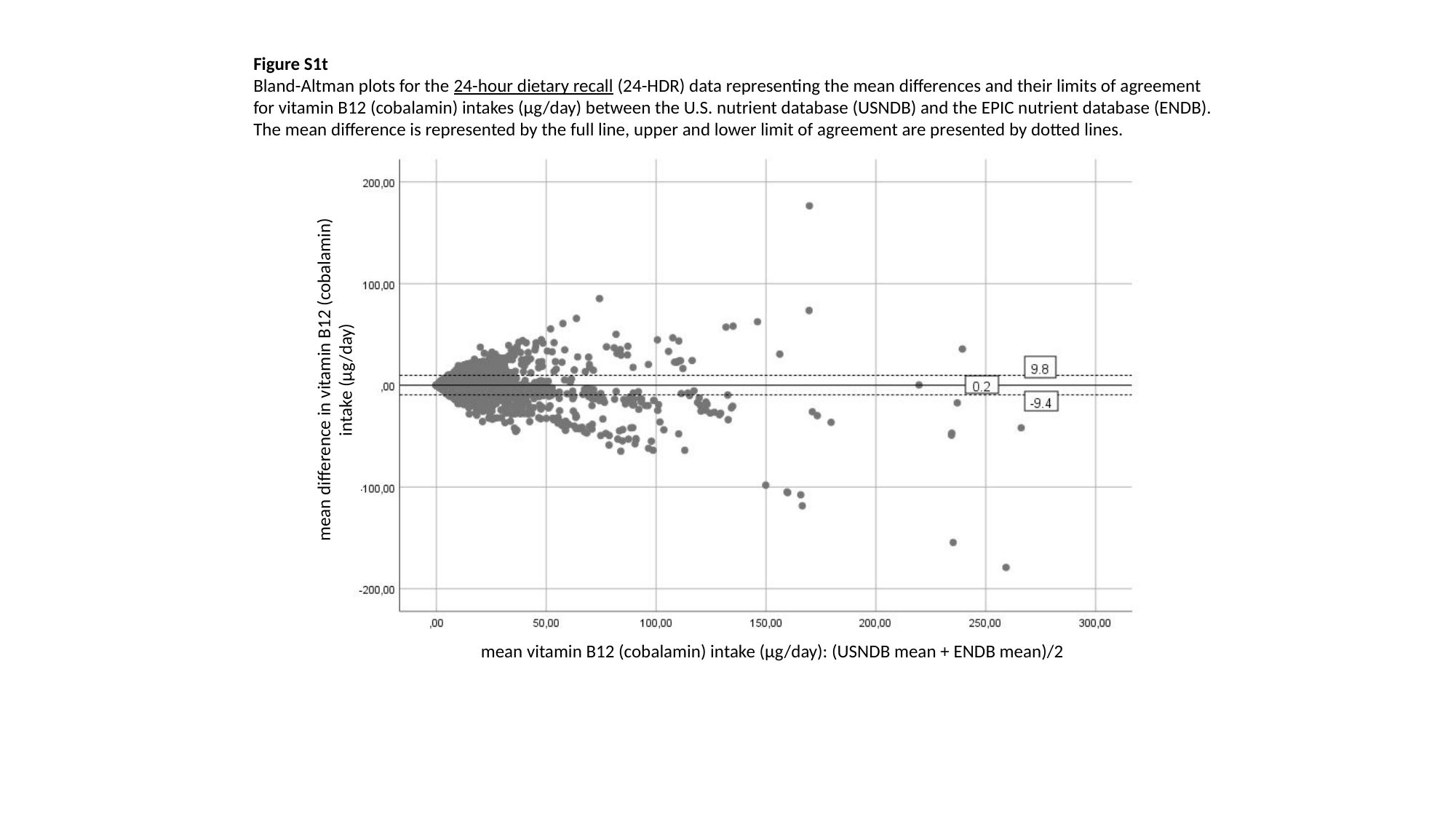

Figure S1t
Bland-Altman plots for the 24-hour dietary recall (24-HDR) data representing the mean differences and their limits of agreement for vitamin B12 (cobalamin) intakes (µg/day) between the U.S. nutrient database (USNDB) and the EPIC nutrient database (ENDB). The mean difference is represented by the full line, upper and lower limit of agreement are presented by dotted lines.
mean difference in vitamin B12 (cobalamin) intake (µg/day)
mean vitamin B12 (cobalamin) intake (µg/day): (USNDB mean + ENDB mean)/2

## Slide 21
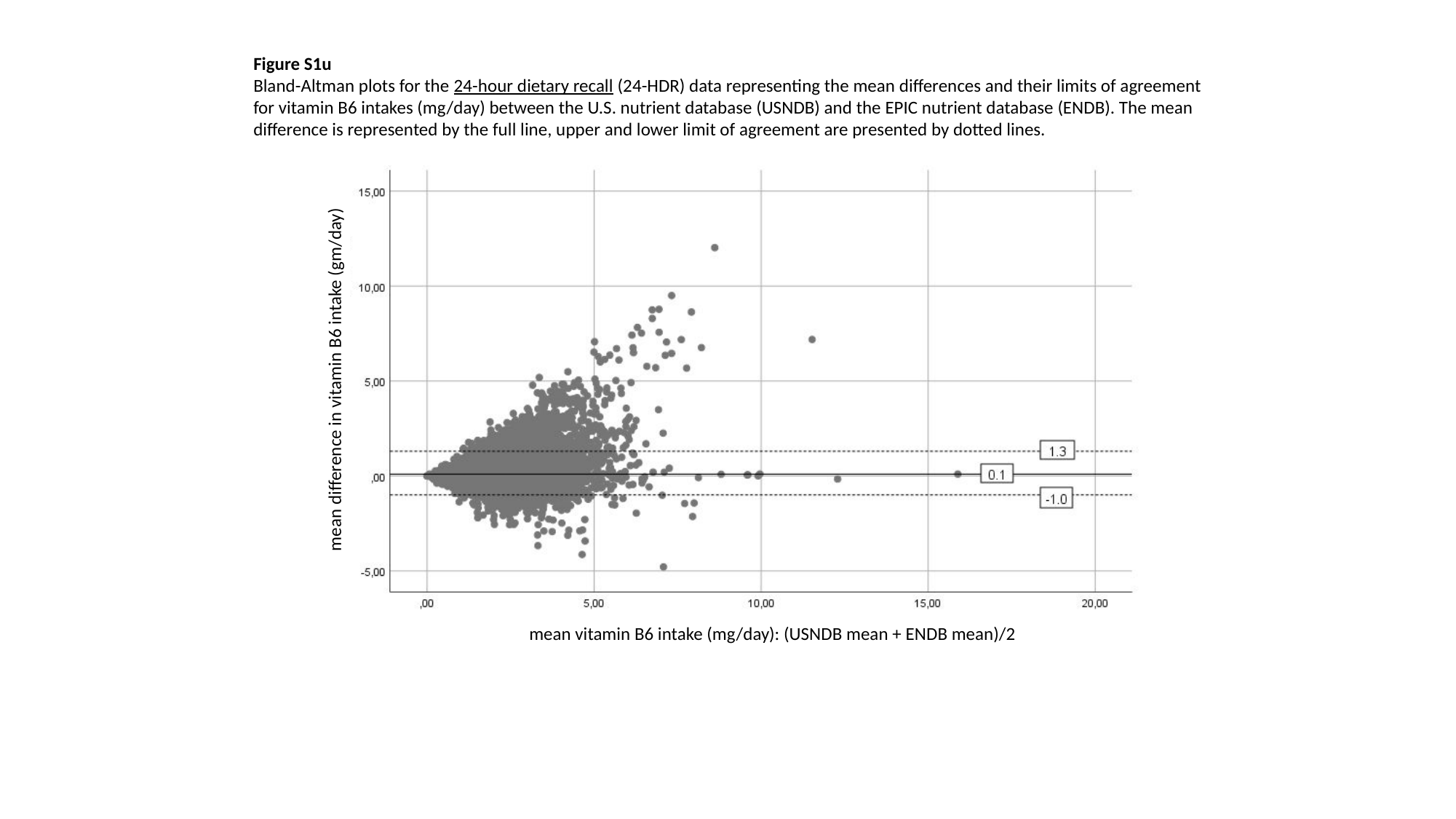

Figure S1u
Bland-Altman plots for the 24-hour dietary recall (24-HDR) data representing the mean differences and their limits of agreement for vitamin B6 intakes (mg/day) between the U.S. nutrient database (USNDB) and the EPIC nutrient database (ENDB). The mean difference is represented by the full line, upper and lower limit of agreement are presented by dotted lines.
mean difference in vitamin B6 intake (gm/day)
mean vitamin B6 intake (mg/day): (USNDB mean + ENDB mean)/2

## Slide 22
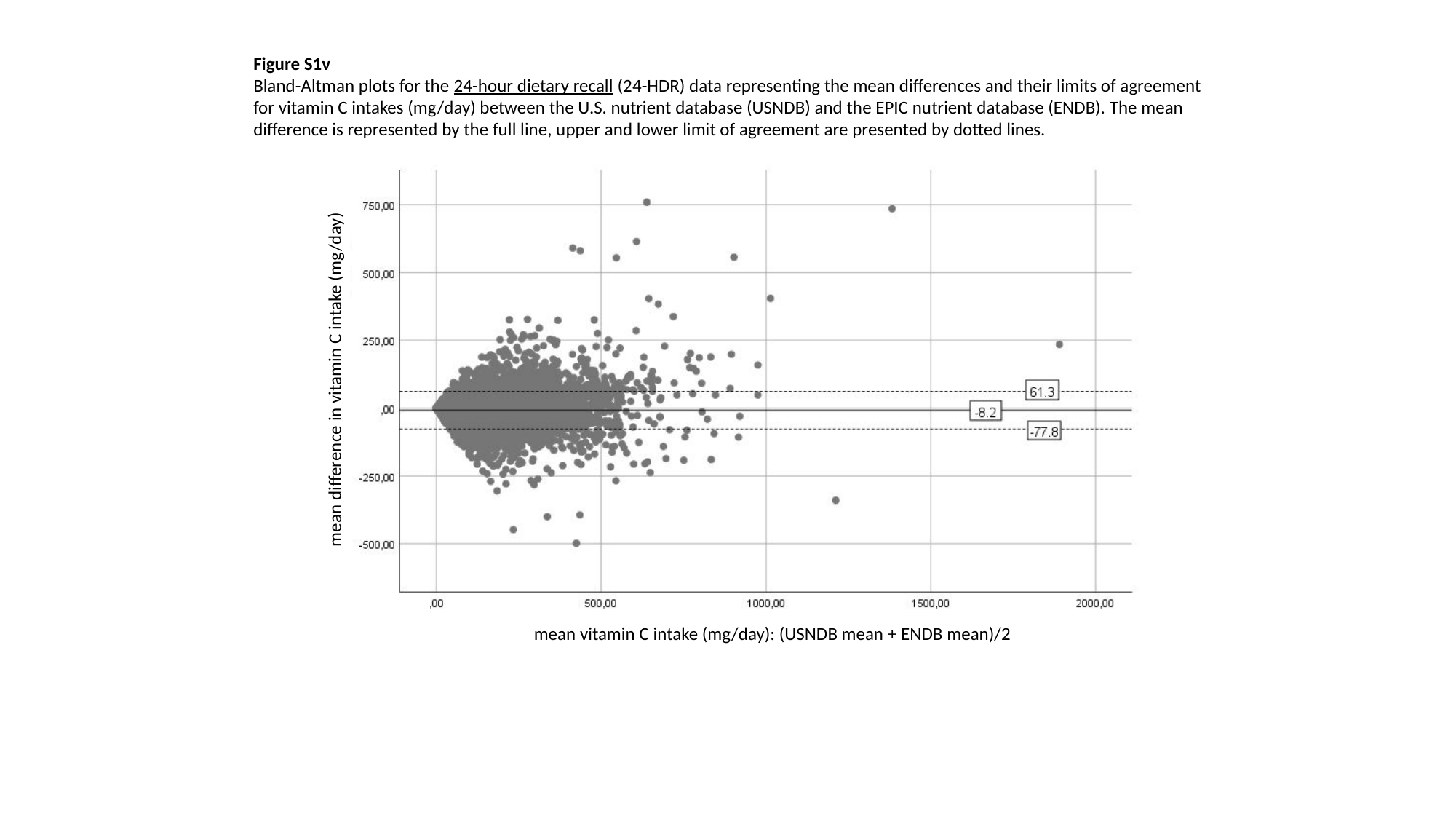

Figure S1v
Bland-Altman plots for the 24-hour dietary recall (24-HDR) data representing the mean differences and their limits of agreement for vitamin C intakes (mg/day) between the U.S. nutrient database (USNDB) and the EPIC nutrient database (ENDB). The mean difference is represented by the full line, upper and lower limit of agreement are presented by dotted lines.
mean difference in vitamin C intake (mg/day)
mean vitamin C intake (mg/day): (USNDB mean + ENDB mean)/2

## Slide 23
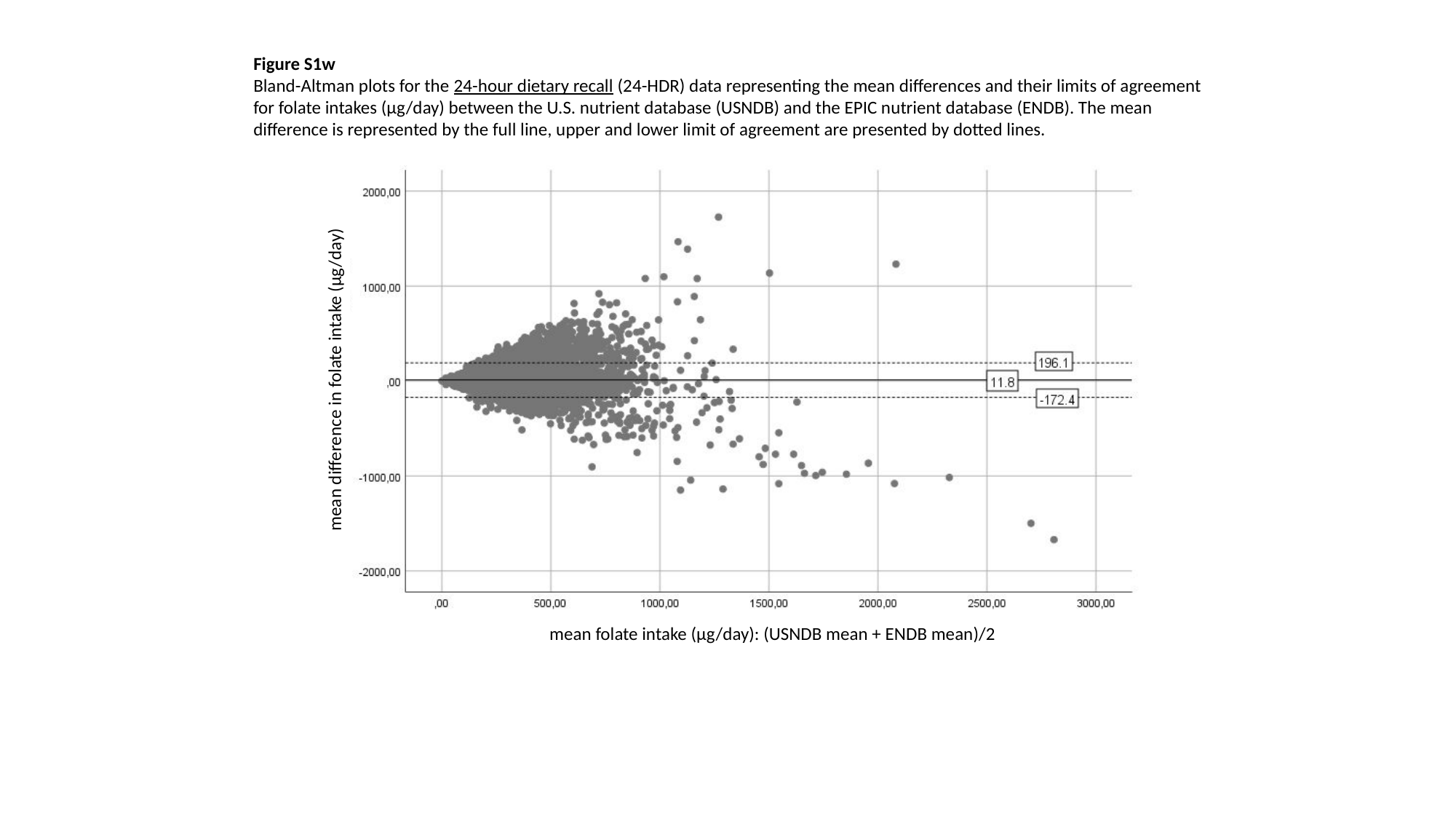

Figure S1w
Bland-Altman plots for the 24-hour dietary recall (24-HDR) data representing the mean differences and their limits of agreement for folate intakes (µg/day) between the U.S. nutrient database (USNDB) and the EPIC nutrient database (ENDB). The mean difference is represented by the full line, upper and lower limit of agreement are presented by dotted lines.
mean difference in folate intake (µg/day)
mean folate intake (µg/day): (USNDB mean + ENDB mean)/2
