# Supplementary material for: Comparing Calculated Nutrient Intakes Using Different Food Composition Databases: Results from the European Prospective Investigation into Cancer and Nutrition (EPIC) Cohort
Source: Nutrients. 2020 Sep 23;12(10):2906. doi: 10.3390/nu12102906 (PMC7650652; doi:10.3390/nu12102906)
Supplement: Supplementary file 1 [file nutrients-12-02906-s001.zip › Revision2_Nutrients_FigureS2.pptx]

## Slide 1
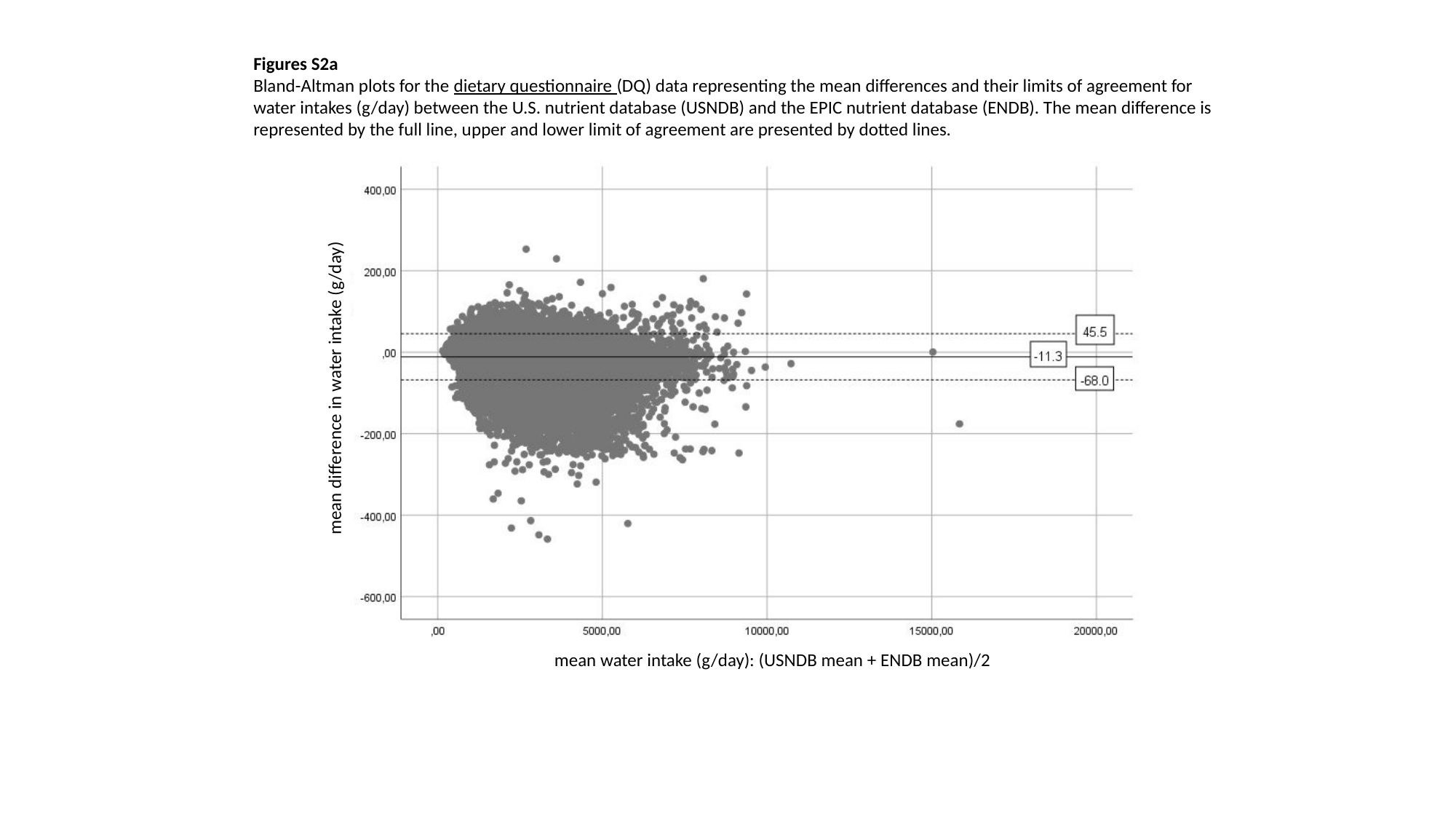

Figures S2a
Bland-Altman plots for the dietary questionnaire (DQ) data representing the mean differences and their limits of agreement for water intakes (g/day) between the U.S. nutrient database (USNDB) and the EPIC nutrient database (ENDB). The mean difference is represented by the full line, upper and lower limit of agreement are presented by dotted lines.
mean difference in water intake (g/day)
mean water intake (g/day): (USNDB mean + ENDB mean)/2

## Slide 2
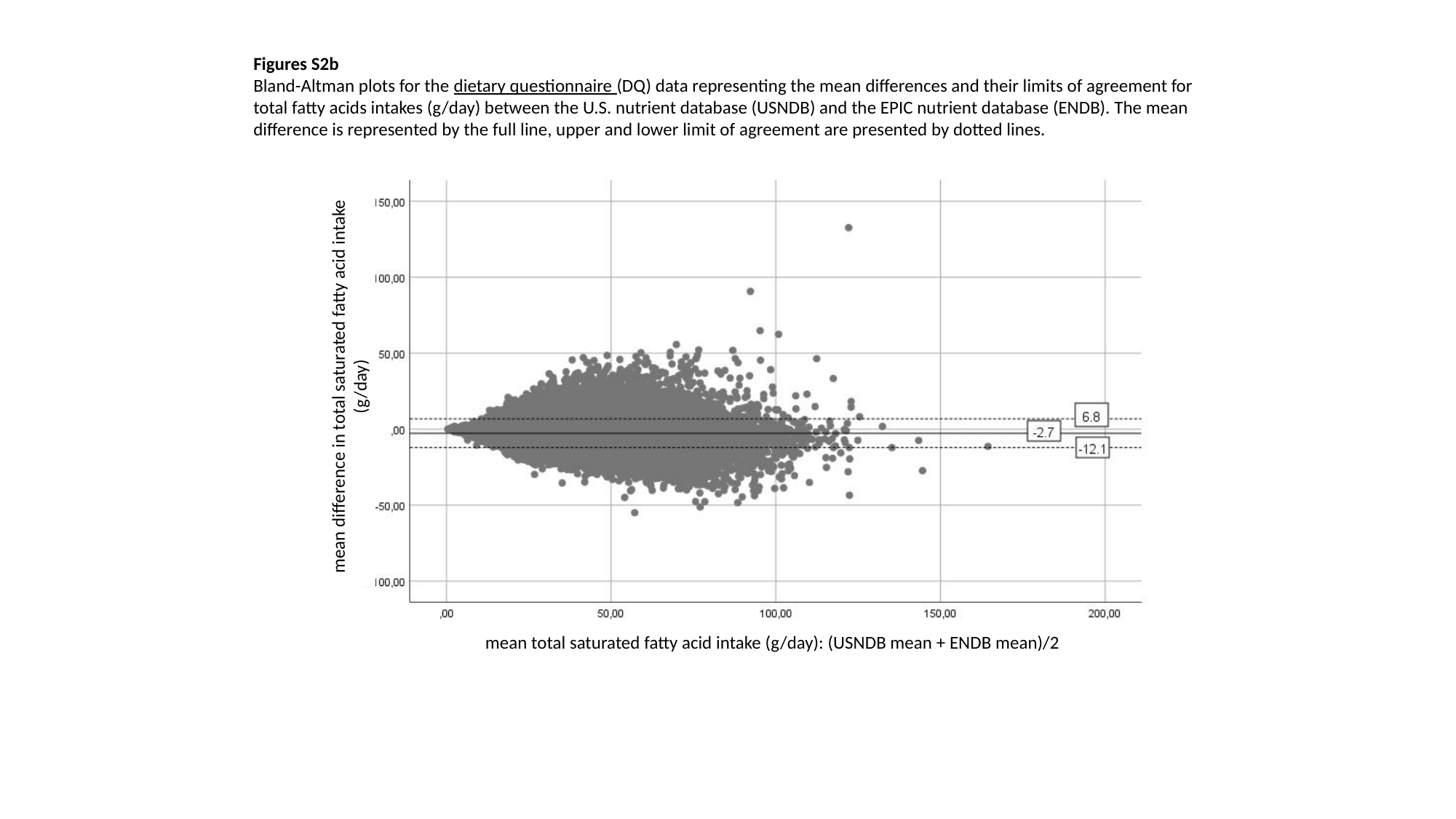

Figures S2b
Bland-Altman plots for the dietary questionnaire (DQ) data representing the mean differences and their limits of agreement for total fatty acids intakes (g/day) between the U.S. nutrient database (USNDB) and the EPIC nutrient database (ENDB). The mean difference is represented by the full line, upper and lower limit of agreement are presented by dotted lines.
mean difference in total saturated fatty acid intake (g/day)
mean difference in folate intake (µg/day)
mean total saturated fatty acid intake (g/day): (USNDB mean + ENDB mean)/2

## Slide 3
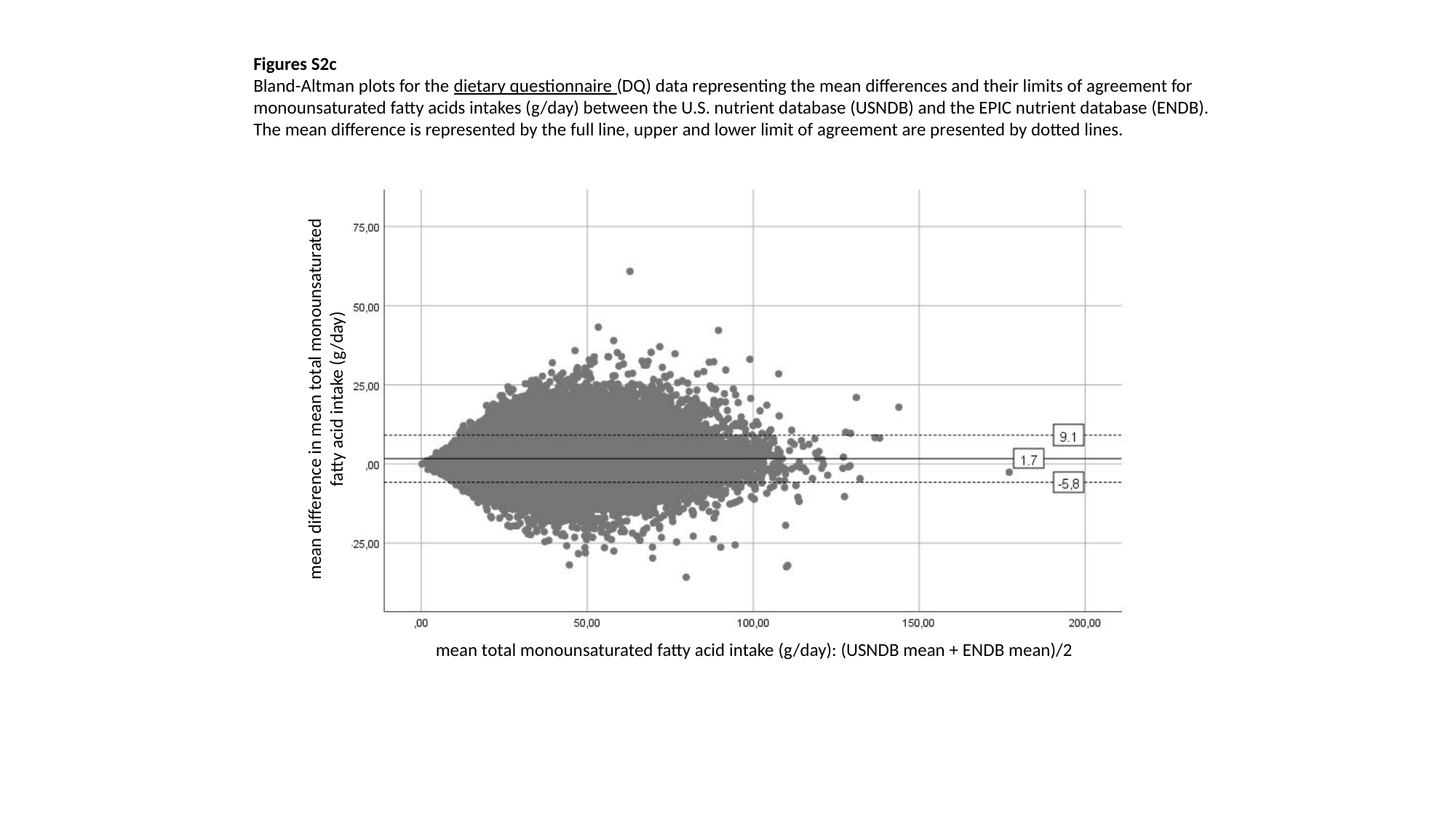

Figures S2c
Bland-Altman plots for the dietary questionnaire (DQ) data representing the mean differences and their limits of agreement for monounsaturated fatty acids intakes (g/day) between the U.S. nutrient database (USNDB) and the EPIC nutrient database (ENDB). The mean difference is represented by the full line, upper and lower limit of agreement are presented by dotted lines.
mean difference in mean total monounsaturated fatty acid intake (g/day)
mean total monounsaturated fatty acid intake (g/day): (USNDB mean + ENDB mean)/2
mean folate intake (µg/day): (USNDB mean + ENDB mean)/2

## Slide 4
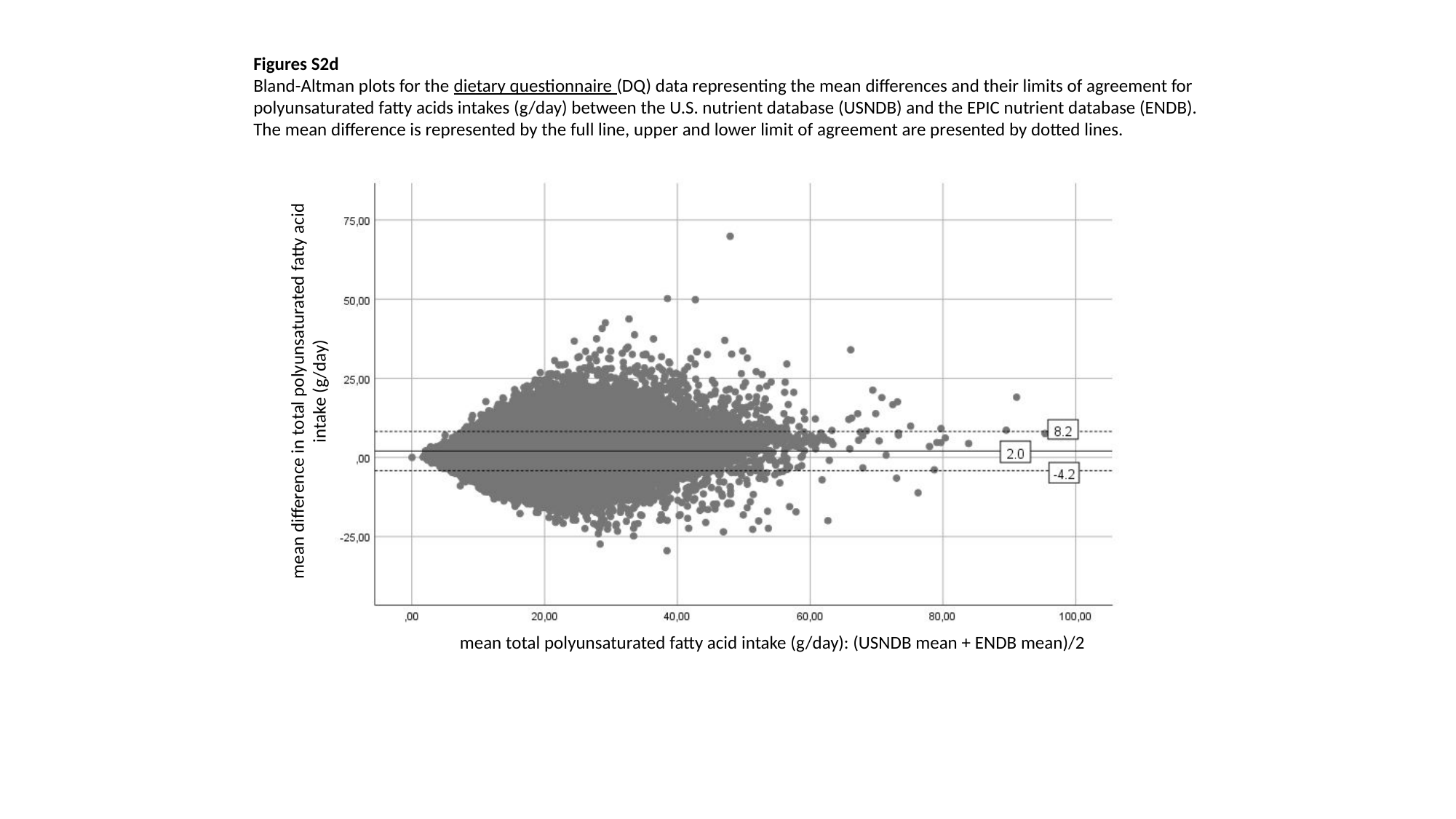

Figures S2d
Bland-Altman plots for the dietary questionnaire (DQ) data representing the mean differences and their limits of agreement for polyunsaturated fatty acids intakes (g/day) between the U.S. nutrient database (USNDB) and the EPIC nutrient database (ENDB). The mean difference is represented by the full line, upper and lower limit of agreement are presented by dotted lines.
mean difference in total polyunsaturated fatty acid intake (g/day)
mean total polyunsaturated fatty acid intake (g/day): (USNDB mean + ENDB mean)/2
mean folate intake (g/day): (USNDB mean + ENDB mean)/2

## Slide 5
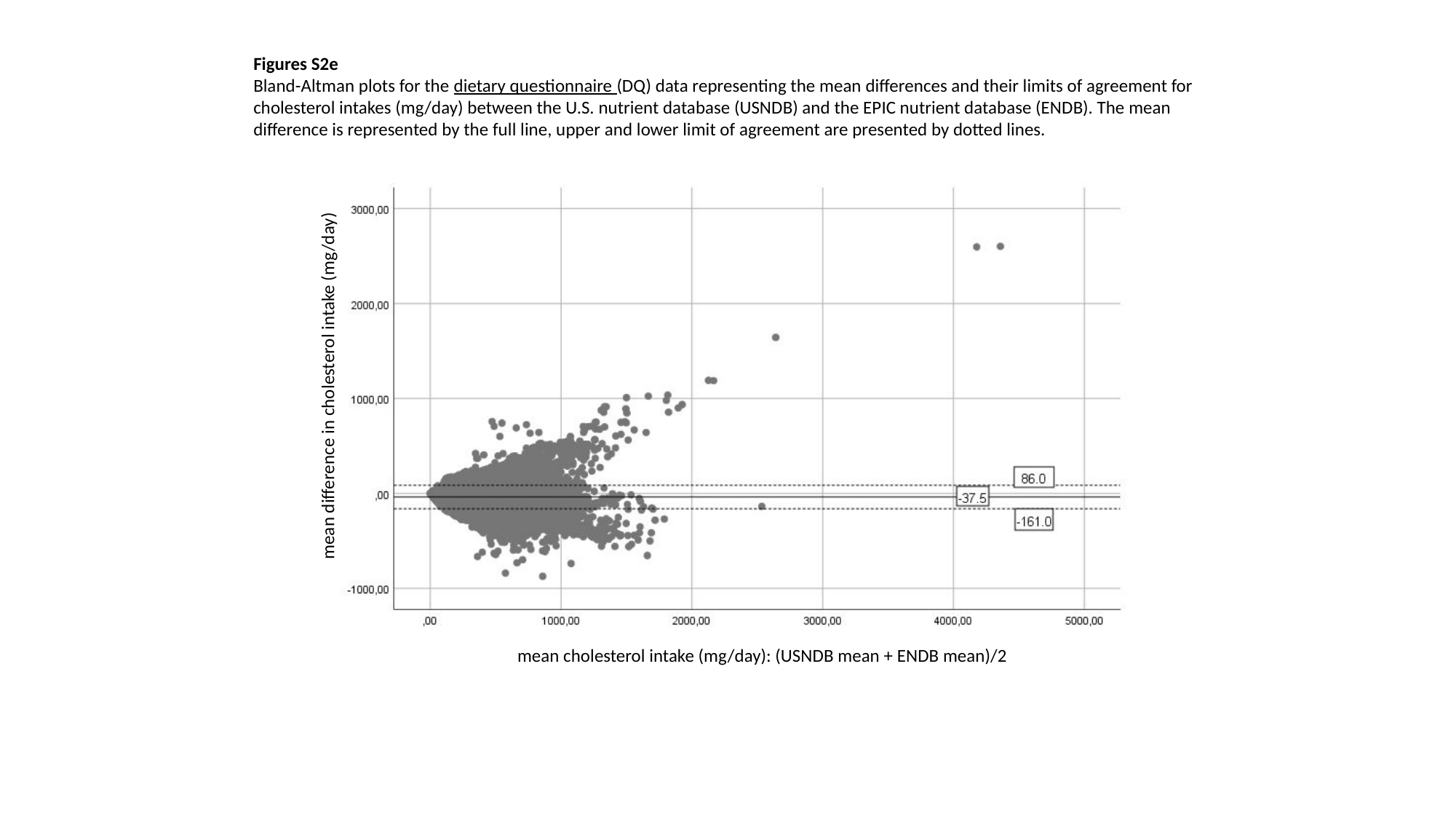

Figures S2e
Bland-Altman plots for the dietary questionnaire (DQ) data representing the mean differences and their limits of agreement for cholesterol intakes (mg/day) between the U.S. nutrient database (USNDB) and the EPIC nutrient database (ENDB). The mean difference is represented by the full line, upper and lower limit of agreement are presented by dotted lines.
mean difference in cholesterol intake (mg/day)
mean cholesterol intake (mg/day): (USNDB mean + ENDB mean)/2

## Slide 6
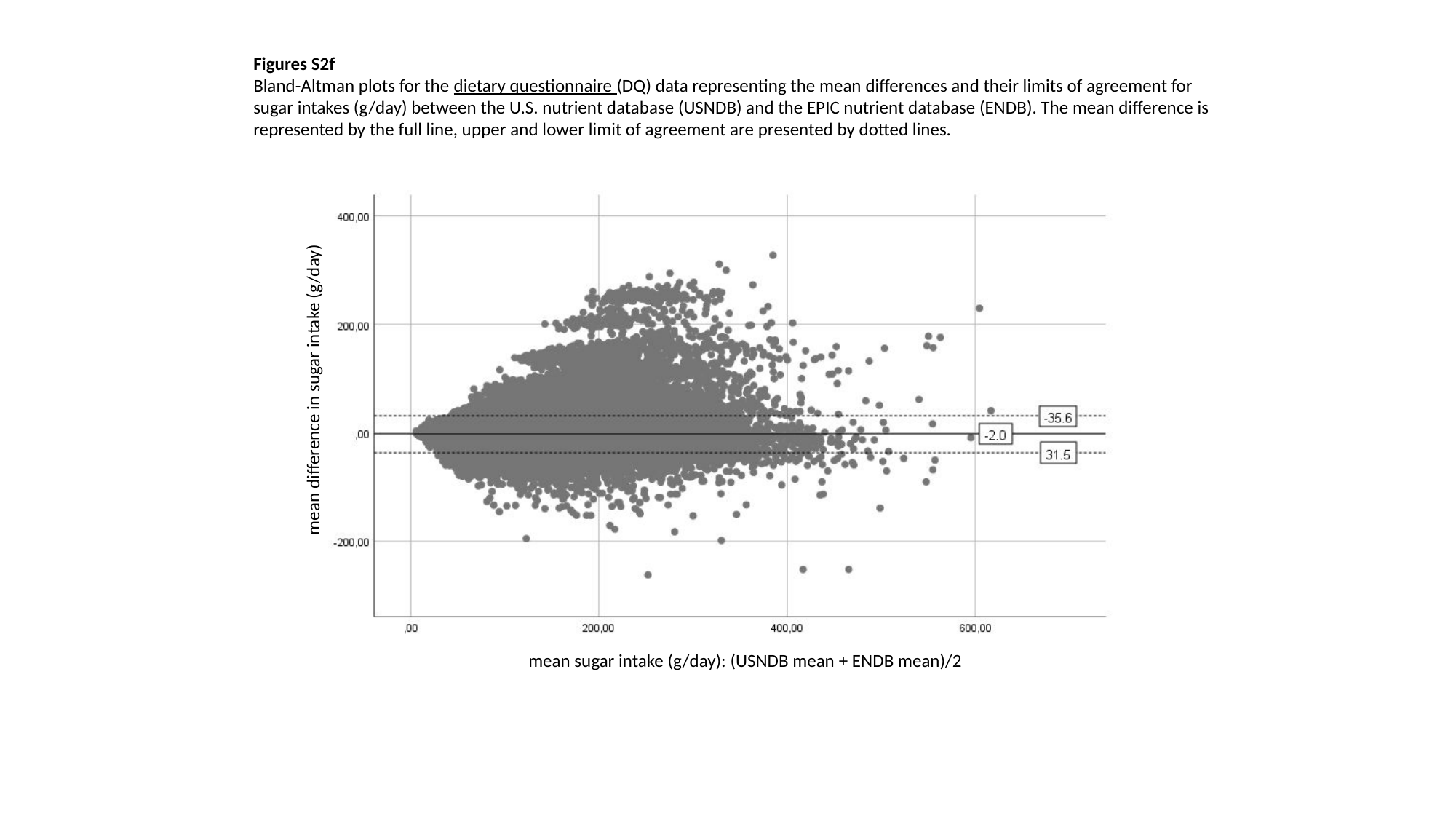

Figures S2f
Bland-Altman plots for the dietary questionnaire (DQ) data representing the mean differences and their limits of agreement for sugar intakes (g/day) between the U.S. nutrient database (USNDB) and the EPIC nutrient database (ENDB). The mean difference is represented by the full line, upper and lower limit of agreement are presented by dotted lines.
mean difference in sugar intake (g/day)
mean sugar intake (g/day): (USNDB mean + ENDB mean)/2

## Slide 7
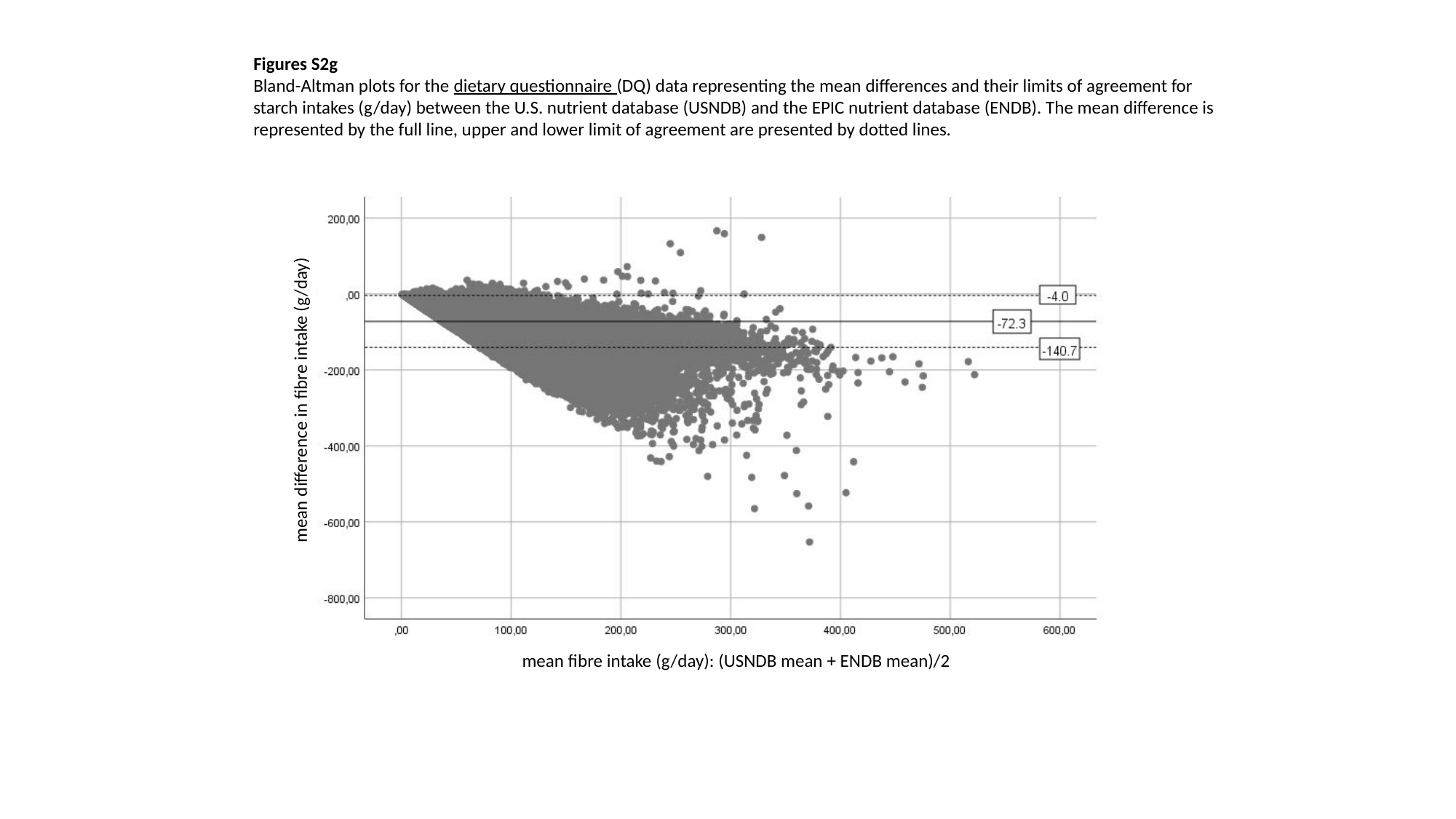

Figures S2g
Bland-Altman plots for the dietary questionnaire (DQ) data representing the mean differences and their limits of agreement for starch intakes (g/day) between the U.S. nutrient database (USNDB) and the EPIC nutrient database (ENDB). The mean difference is represented by the full line, upper and lower limit of agreement are presented by dotted lines.
mean difference in fibre intake (g/day)
mean fibre intake (g/day): (USNDB mean + ENDB mean)/2

## Slide 8
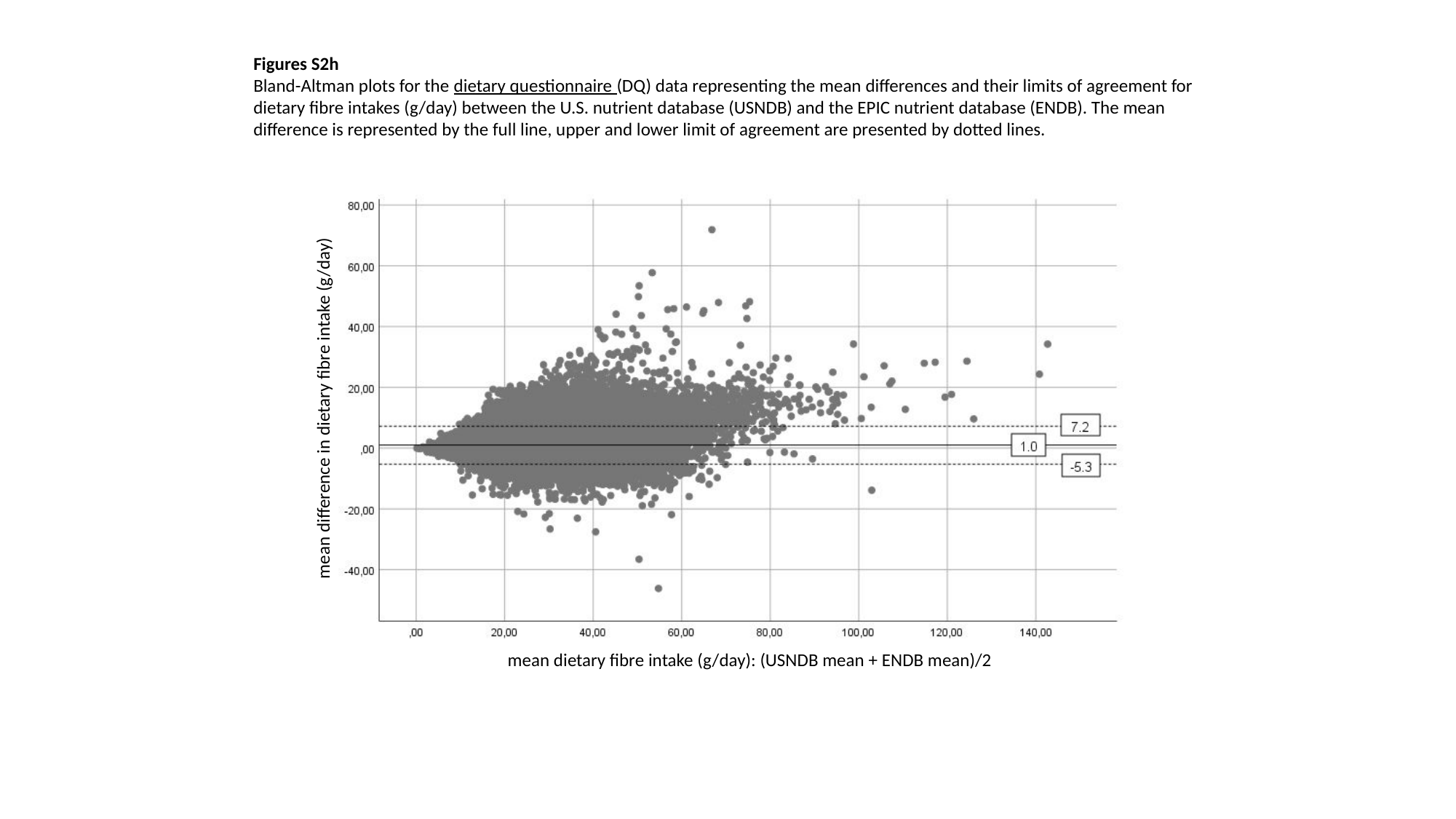

Figures S2h
Bland-Altman plots for the dietary questionnaire (DQ) data representing the mean differences and their limits of agreement for dietary fibre intakes (g/day) between the U.S. nutrient database (USNDB) and the EPIC nutrient database (ENDB). The mean difference is represented by the full line, upper and lower limit of agreement are presented by dotted lines.
mean difference in dietary fibre intake (g/day)
mean dietary fibre intake (g/day): (USNDB mean + ENDB mean)/2

## Slide 9
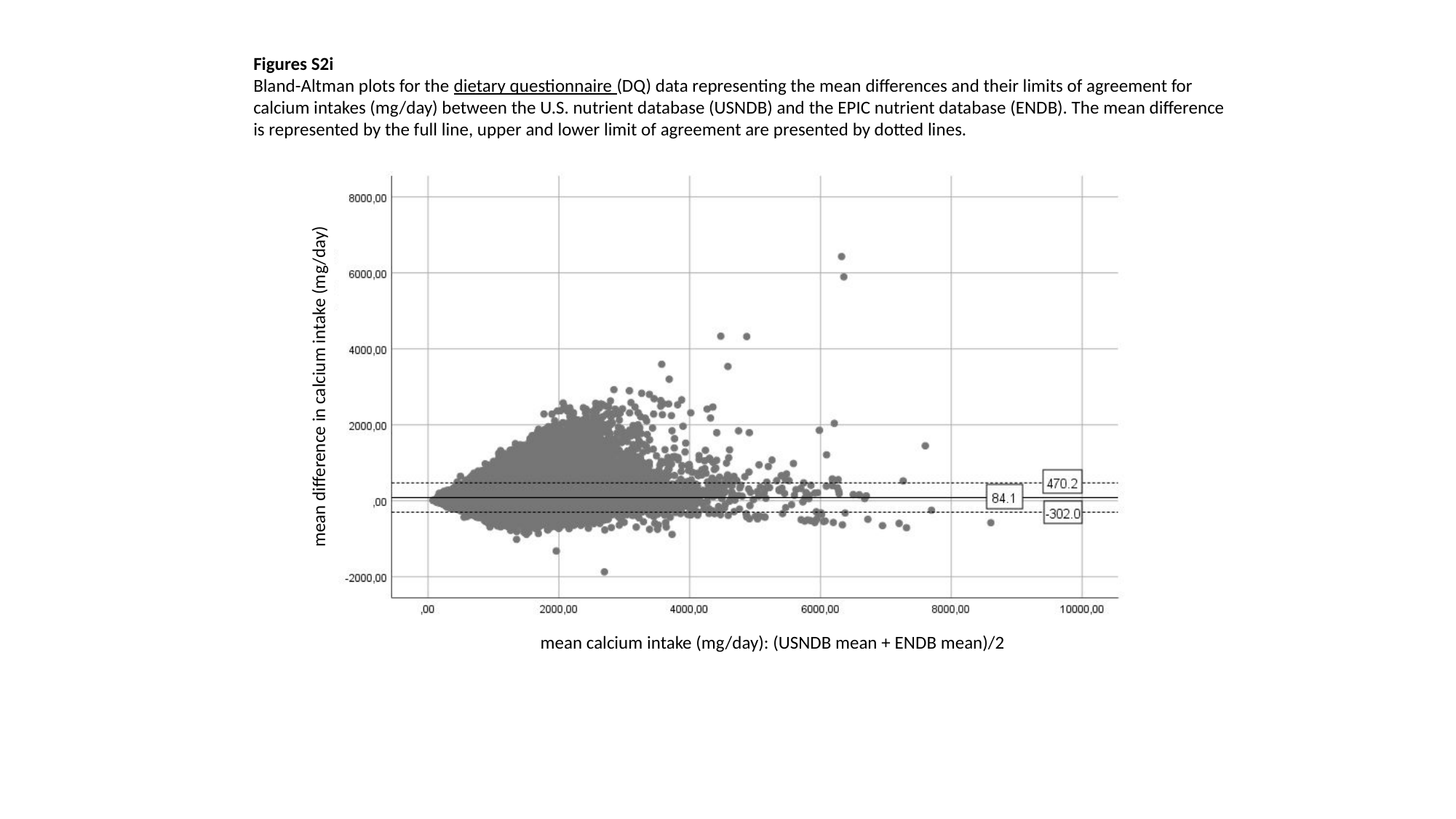

Figures S2i
Bland-Altman plots for the dietary questionnaire (DQ) data representing the mean differences and their limits of agreement for calcium intakes (mg/day) between the U.S. nutrient database (USNDB) and the EPIC nutrient database (ENDB). The mean difference is represented by the full line, upper and lower limit of agreement are presented by dotted lines.
mean difference in calcium intake (mg/day)
mean calcium intake (mg/day): (USNDB mean + ENDB mean)/2

## Slide 10
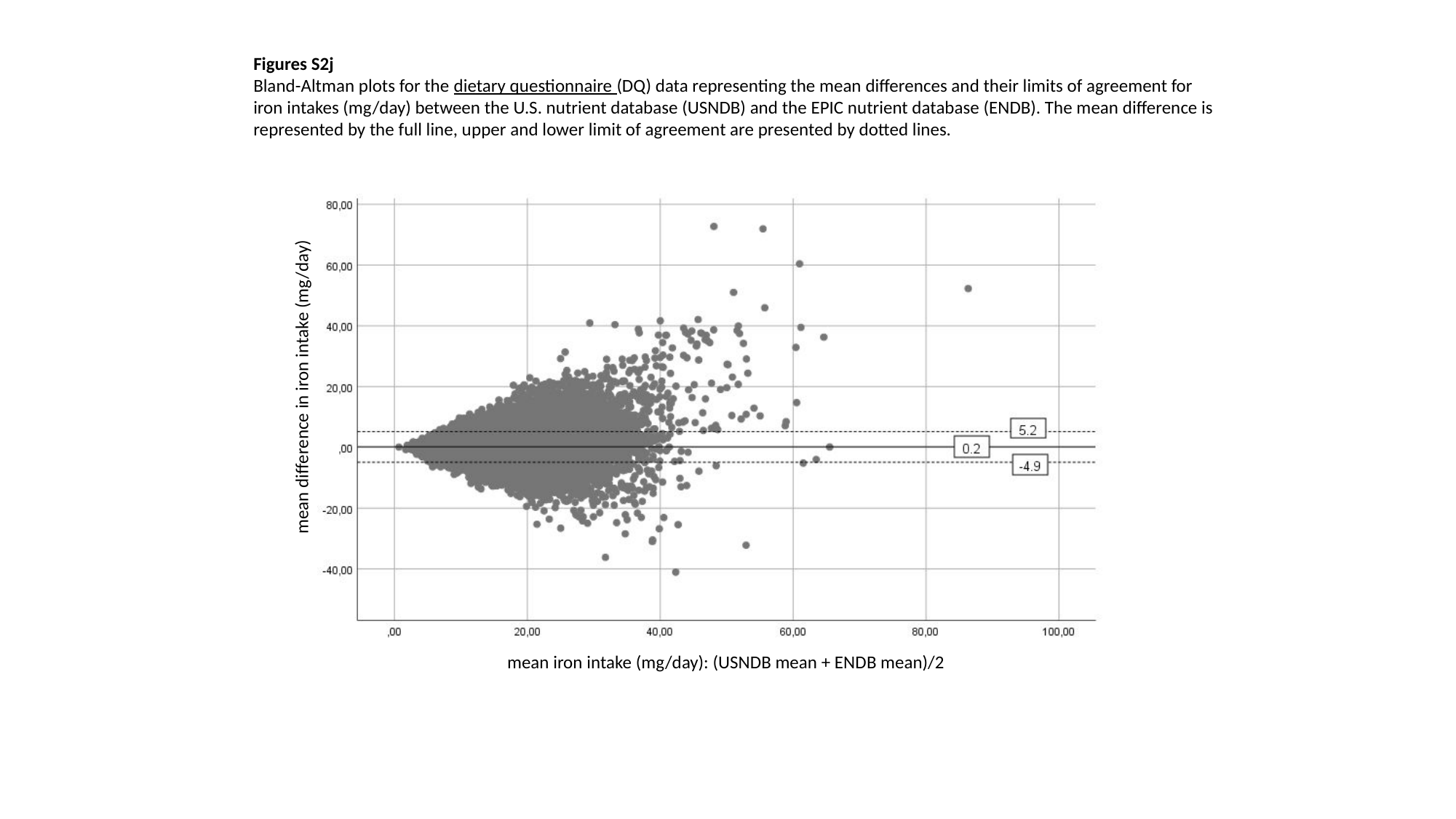

Figures S2j
Bland-Altman plots for the dietary questionnaire (DQ) data representing the mean differences and their limits of agreement for iron intakes (mg/day) between the U.S. nutrient database (USNDB) and the EPIC nutrient database (ENDB). The mean difference is represented by the full line, upper and lower limit of agreement are presented by dotted lines.
mean difference in iron intake (mg/day)
mean iron intake (mg/day): (USNDB mean + ENDB mean)/2

## Slide 11
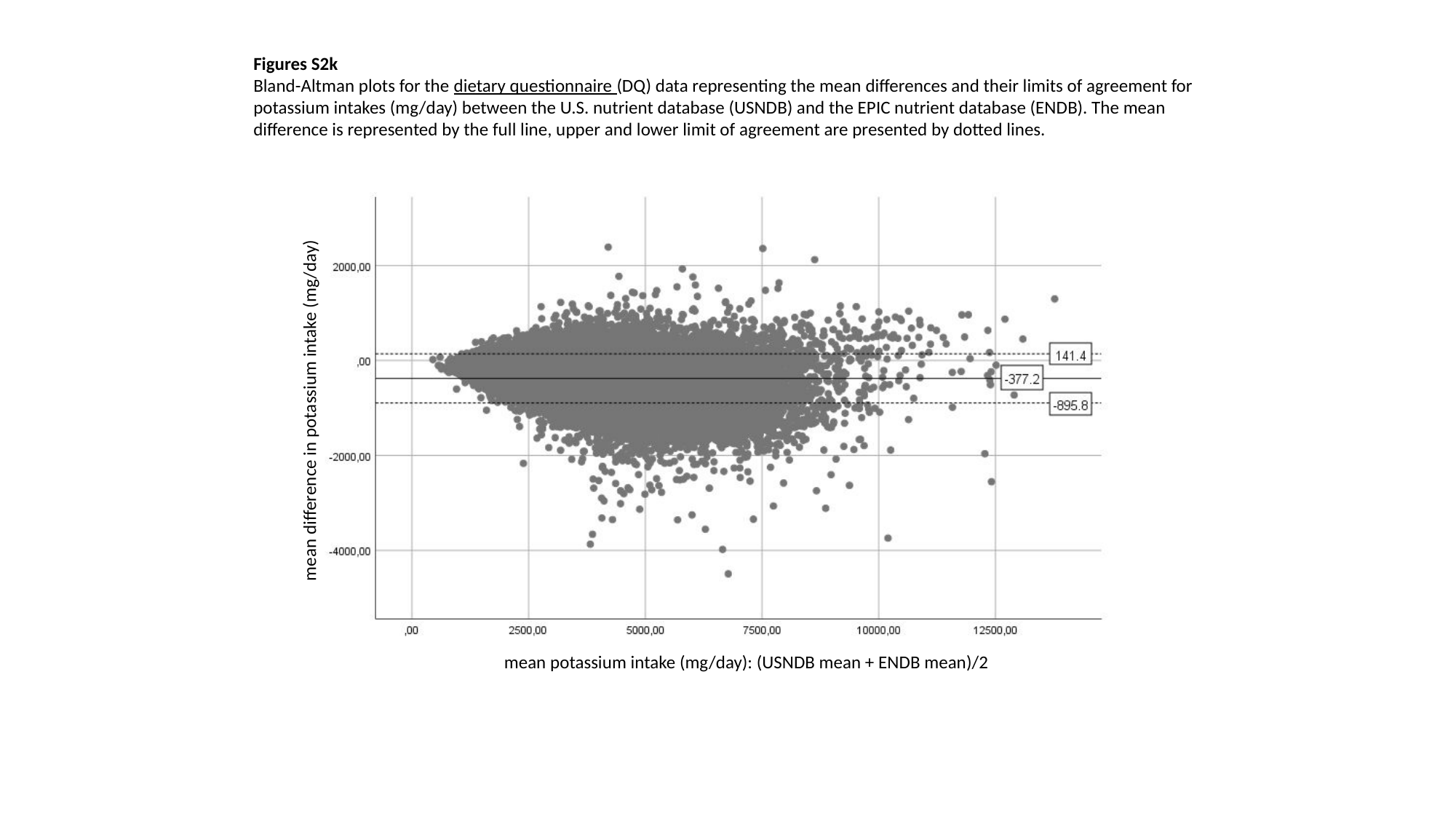

Figures S2k
Bland-Altman plots for the dietary questionnaire (DQ) data representing the mean differences and their limits of agreement for potassium intakes (mg/day) between the U.S. nutrient database (USNDB) and the EPIC nutrient database (ENDB). The mean difference is represented by the full line, upper and lower limit of agreement are presented by dotted lines.
mean difference in potassium intake (mg/day)
mean potassium intake (mg/day): (USNDB mean + ENDB mean)/2

## Slide 12
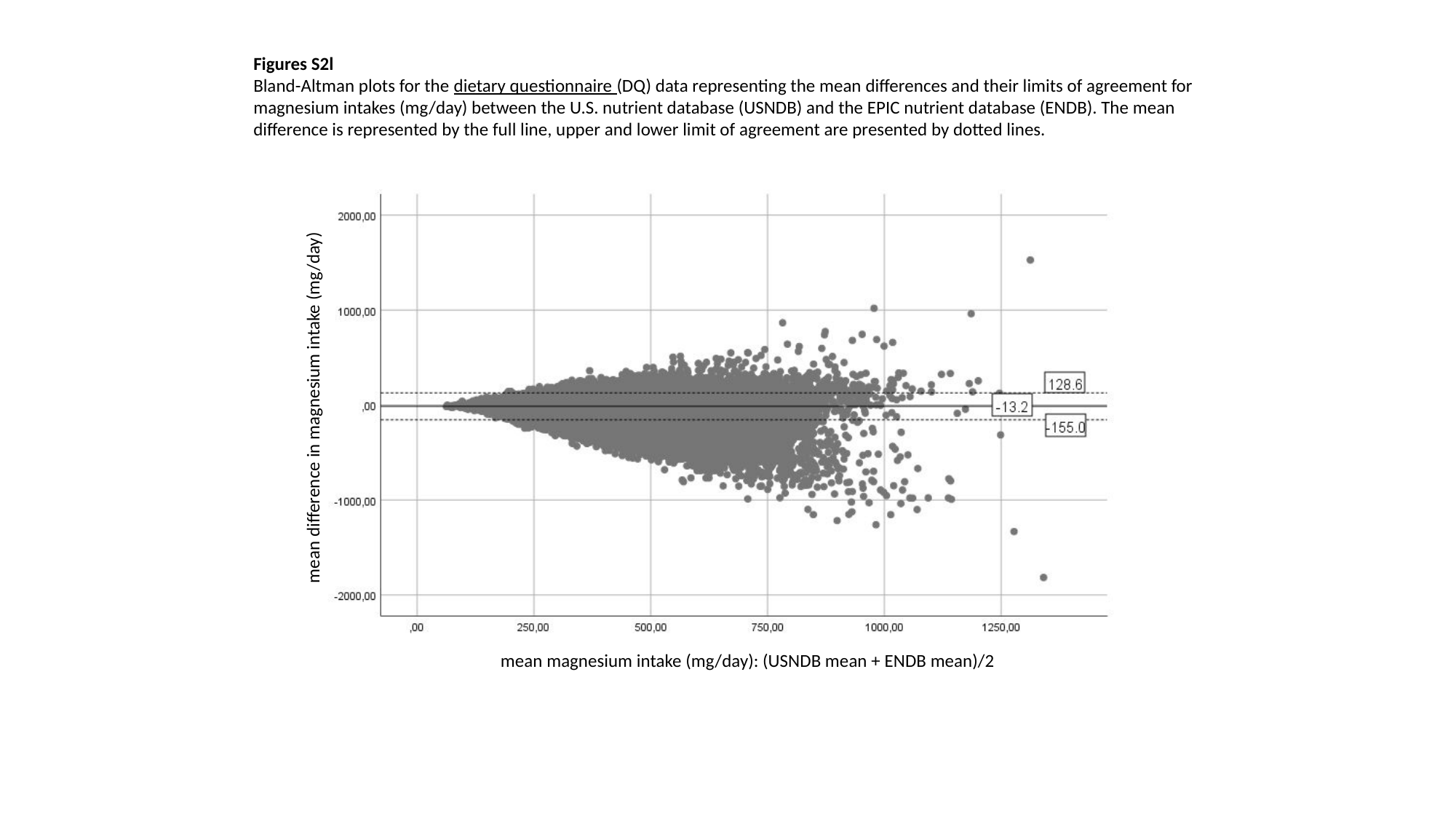

Figures S2l
Bland-Altman plots for the dietary questionnaire (DQ) data representing the mean differences and their limits of agreement for magnesium intakes (mg/day) between the U.S. nutrient database (USNDB) and the EPIC nutrient database (ENDB). The mean difference is represented by the full line, upper and lower limit of agreement are presented by dotted lines.
mean difference in magnesium intake (mg/day)
mean magnesium intake (mg/day): (USNDB mean + ENDB mean)/2

## Slide 13
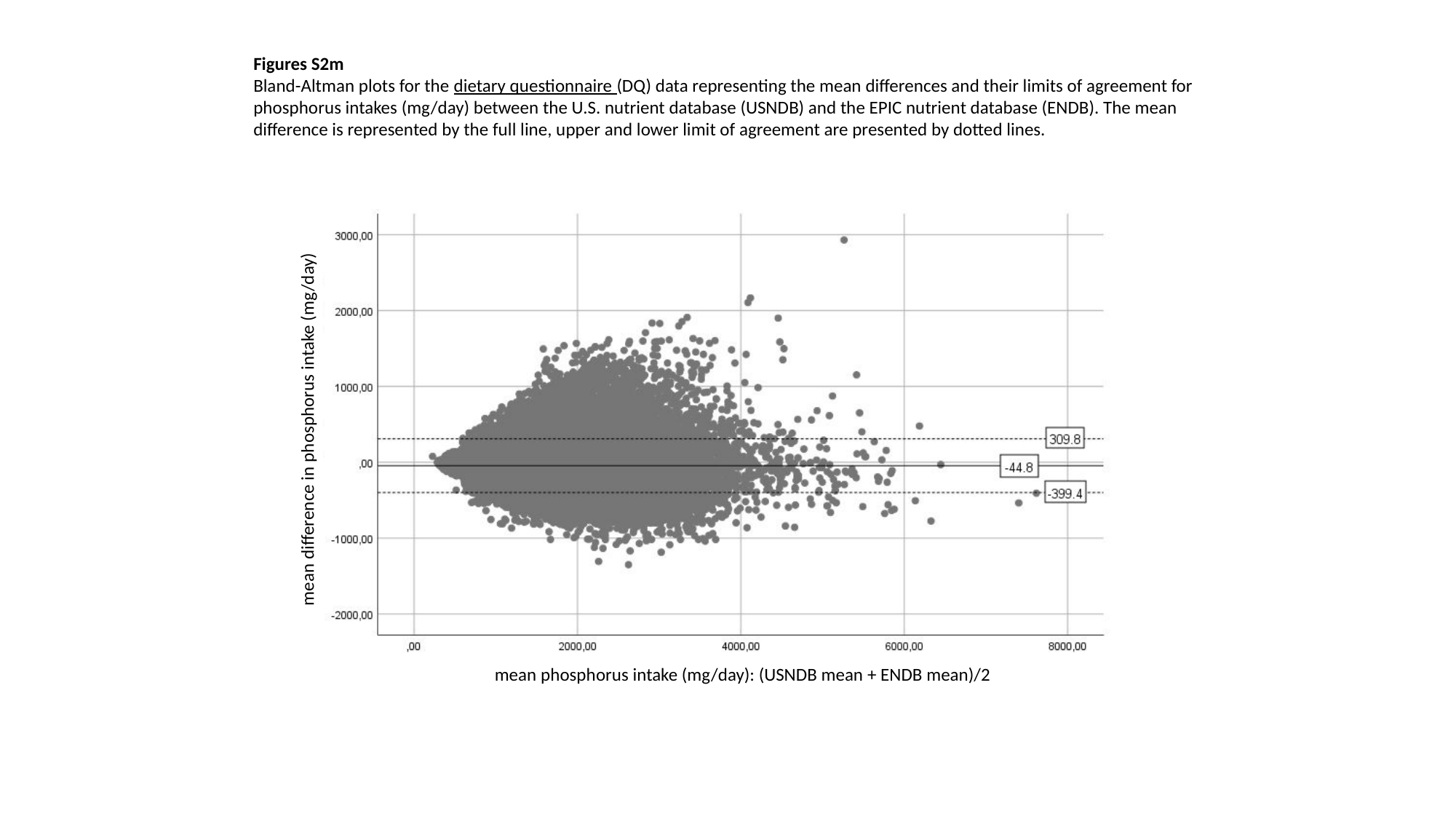

Figures S2m
Bland-Altman plots for the dietary questionnaire (DQ) data representing the mean differences and their limits of agreement for phosphorus intakes (mg/day) between the U.S. nutrient database (USNDB) and the EPIC nutrient database (ENDB). The mean difference is represented by the full line, upper and lower limit of agreement are presented by dotted lines.
mean difference in phosphorus intake (mg/day)
mean phosphorus intake (mg/day): (USNDB mean + ENDB mean)/2

## Slide 14
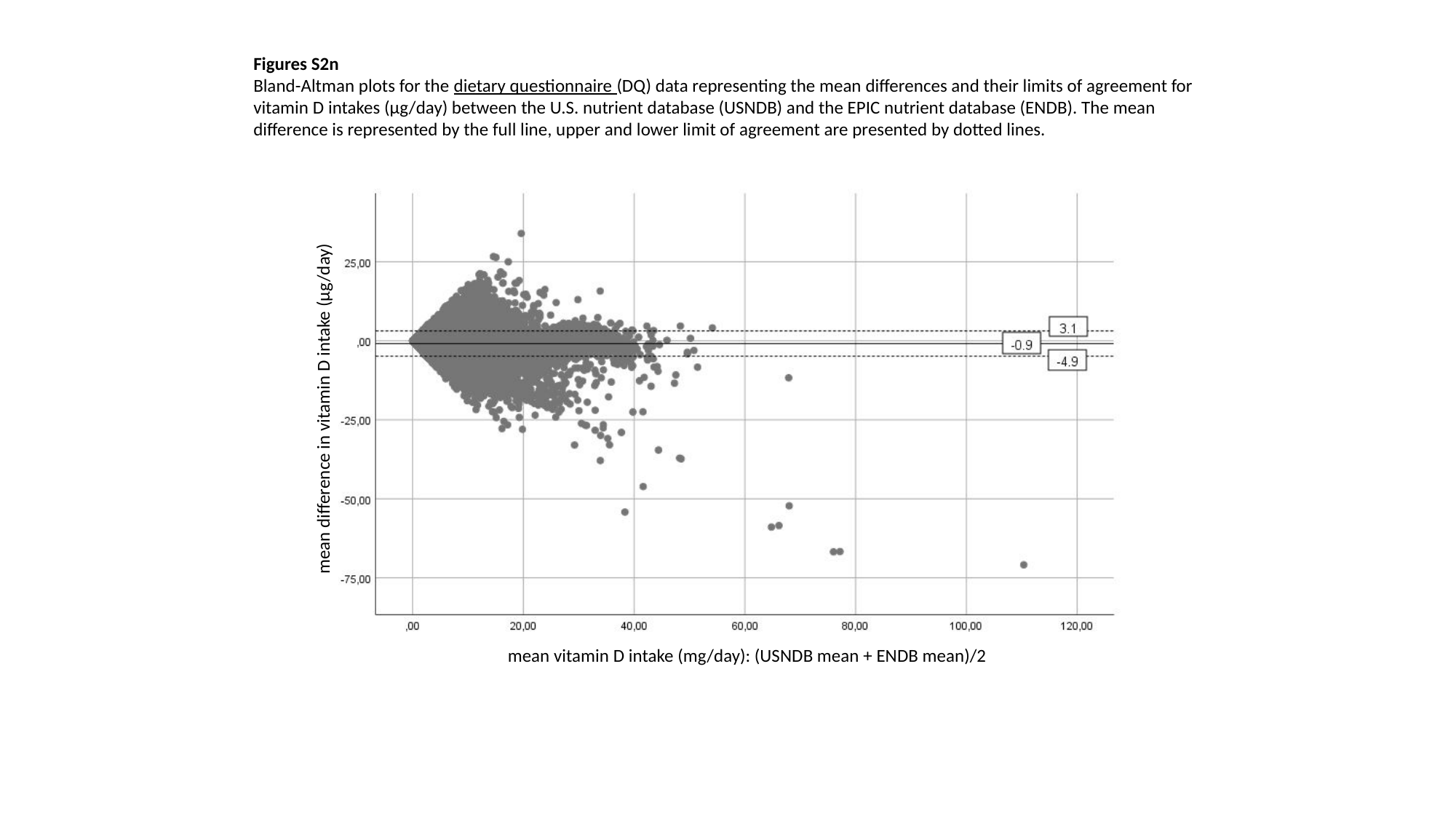

Figures S2n
Bland-Altman plots for the dietary questionnaire (DQ) data representing the mean differences and their limits of agreement for vitamin D intakes (µg/day) between the U.S. nutrient database (USNDB) and the EPIC nutrient database (ENDB). The mean difference is represented by the full line, upper and lower limit of agreement are presented by dotted lines.
mean difference in vitamin D intake (µg/day)
mean vitamin D intake (mg/day): (USNDB mean + ENDB mean)/2

## Slide 15
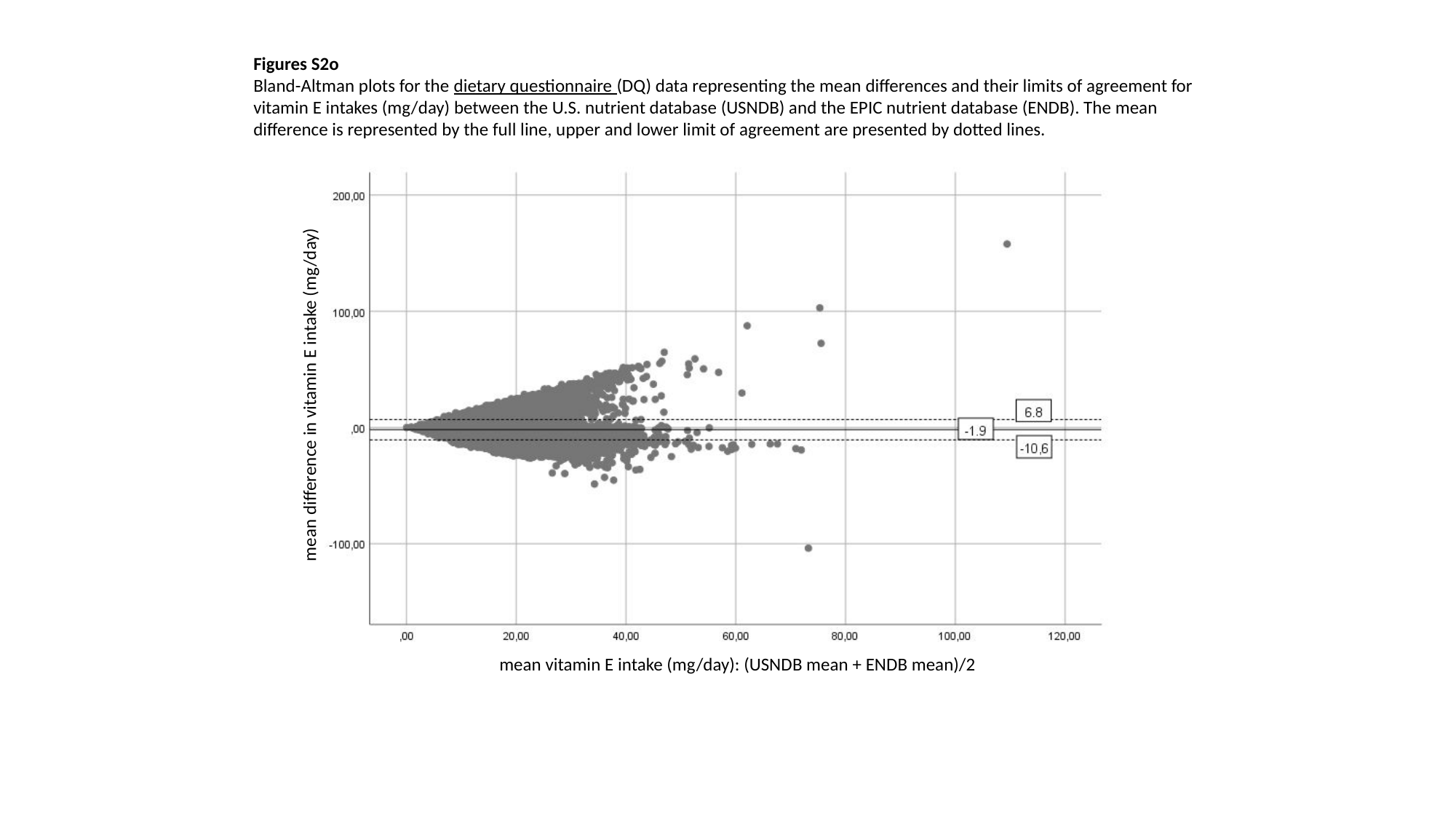

Figures S2o
Bland-Altman plots for the dietary questionnaire (DQ) data representing the mean differences and their limits of agreement for vitamin E intakes (mg/day) between the U.S. nutrient database (USNDB) and the EPIC nutrient database (ENDB). The mean difference is represented by the full line, upper and lower limit of agreement are presented by dotted lines.
mean difference in vitamin E intake (mg/day)
mean vitamin E intake (mg/day): (USNDB mean + ENDB mean)/2

## Slide 16
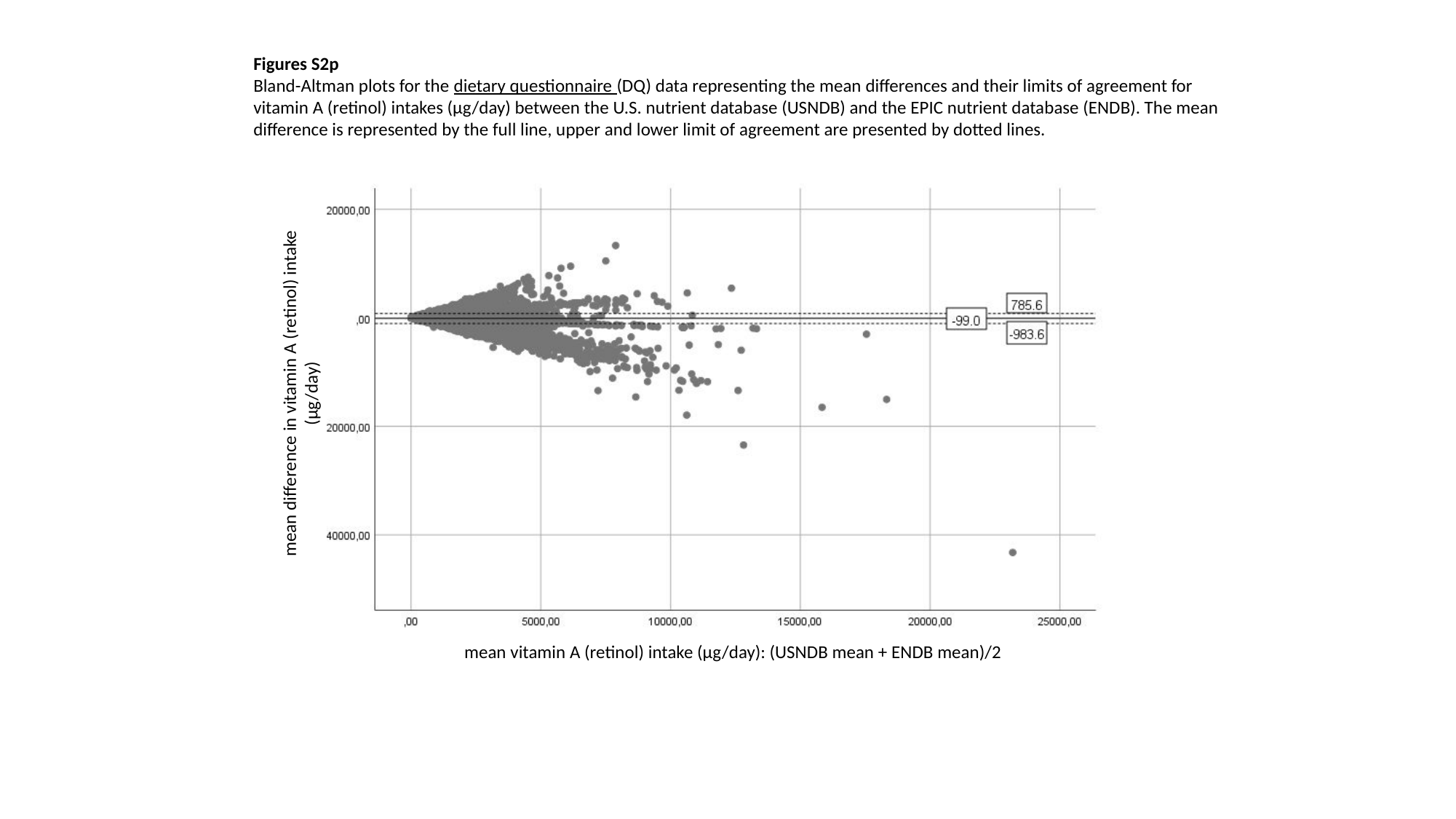

Figures S2p
Bland-Altman plots for the dietary questionnaire (DQ) data representing the mean differences and their limits of agreement for vitamin A (retinol) intakes (µg/day) between the U.S. nutrient database (USNDB) and the EPIC nutrient database (ENDB). The mean difference is represented by the full line, upper and lower limit of agreement are presented by dotted lines.
mean difference in vitamin A (retinol) intake (µg/day)
mean vitamin A (retinol) intake (µg/day): (USNDB mean + ENDB mean)/2

## Slide 17
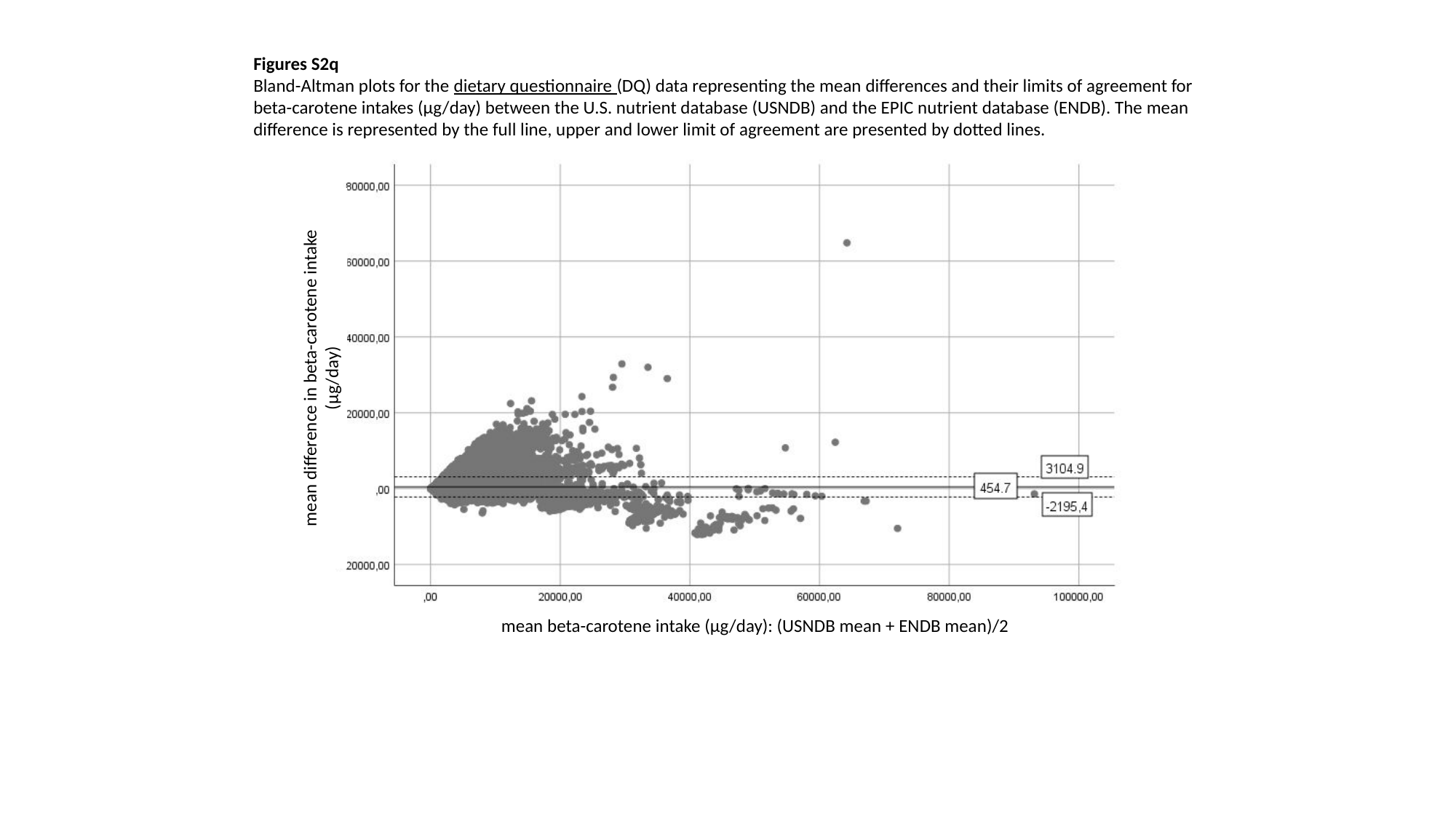

Figures S2q
Bland-Altman plots for the dietary questionnaire (DQ) data representing the mean differences and their limits of agreement for beta-carotene intakes (µg/day) between the U.S. nutrient database (USNDB) and the EPIC nutrient database (ENDB). The mean difference is represented by the full line, upper and lower limit of agreement are presented by dotted lines.
mean difference in beta-carotene intake (µg/day)
mean beta-carotene intake (µg/day): (USNDB mean + ENDB mean)/2

## Slide 18
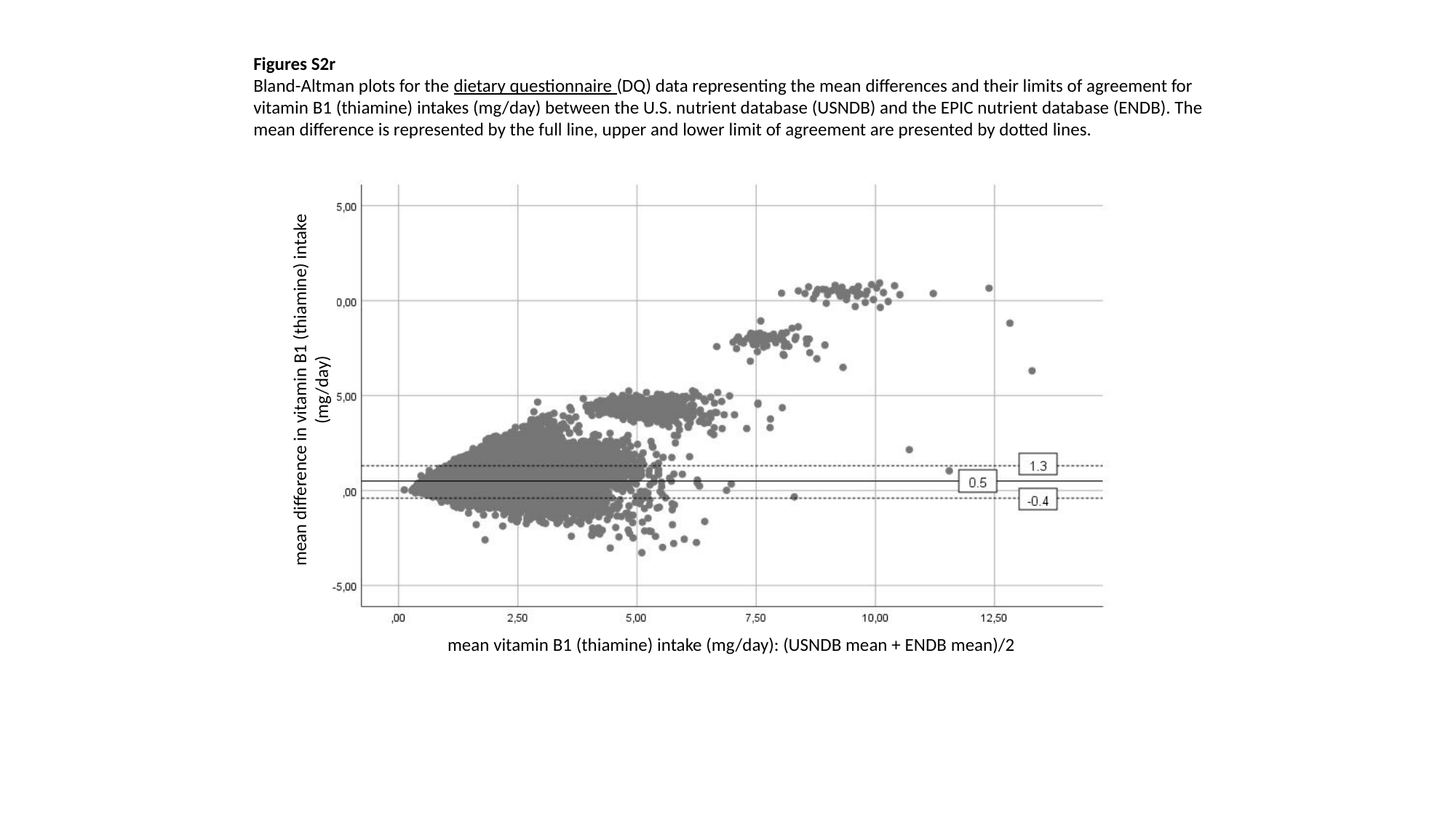

Figures S2r
Bland-Altman plots for the dietary questionnaire (DQ) data representing the mean differences and their limits of agreement for vitamin B1 (thiamine) intakes (mg/day) between the U.S. nutrient database (USNDB) and the EPIC nutrient database (ENDB). The mean difference is represented by the full line, upper and lower limit of agreement are presented by dotted lines.
mean difference in vitamin B1 (thiamine) intake (mg/day)
mean vitamin B1 (thiamine) intake (mg/day): (USNDB mean + ENDB mean)/2

## Slide 19
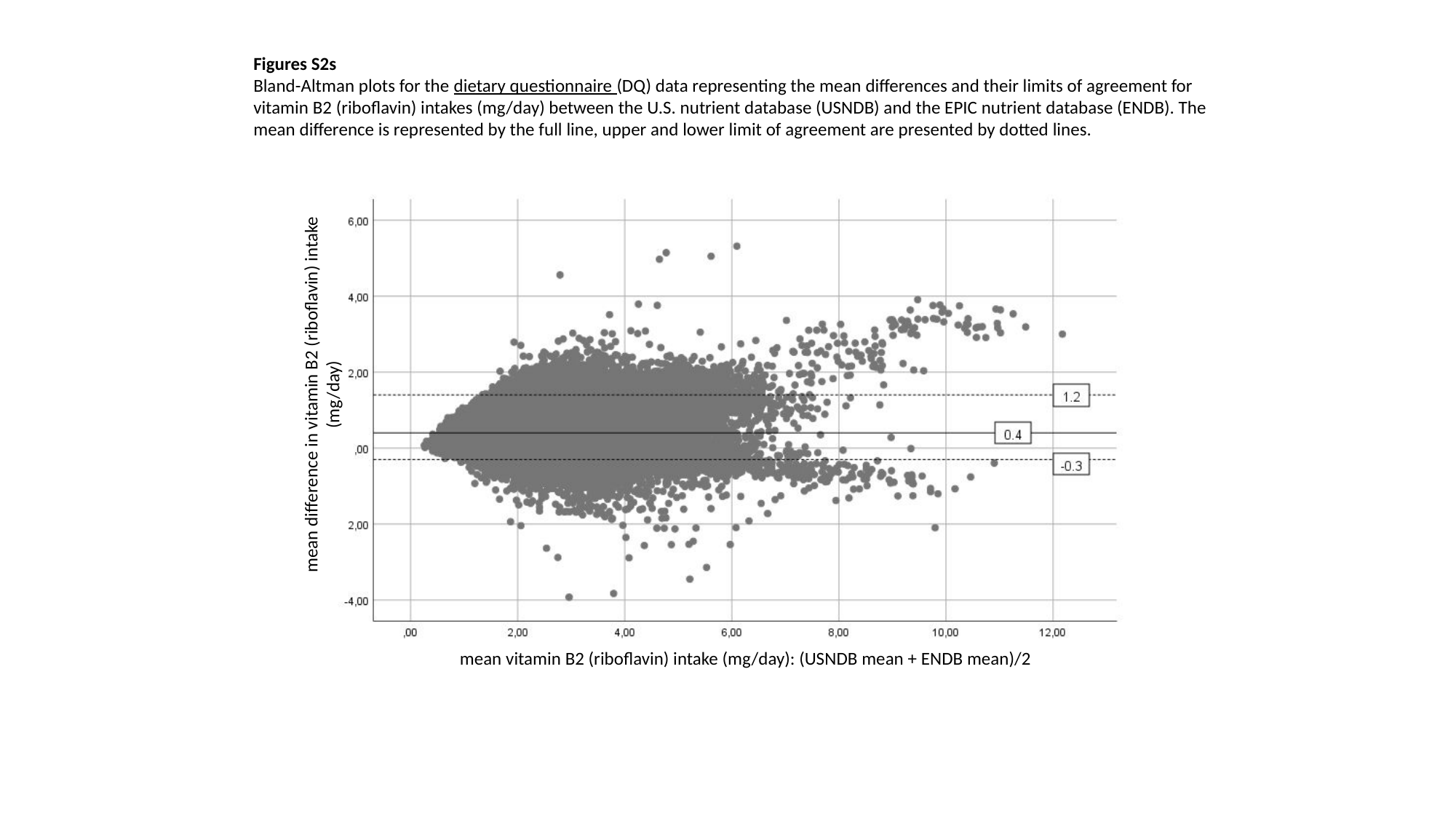

Figures S2s
Bland-Altman plots for the dietary questionnaire (DQ) data representing the mean differences and their limits of agreement for vitamin B2 (riboflavin) intakes (mg/day) between the U.S. nutrient database (USNDB) and the EPIC nutrient database (ENDB). The mean difference is represented by the full line, upper and lower limit of agreement are presented by dotted lines.
mean difference in vitamin B2 (riboflavin) intake (mg/day)
mean vitamin B2 (riboflavin) intake (mg/day): (USNDB mean + ENDB mean)/2

## Slide 20
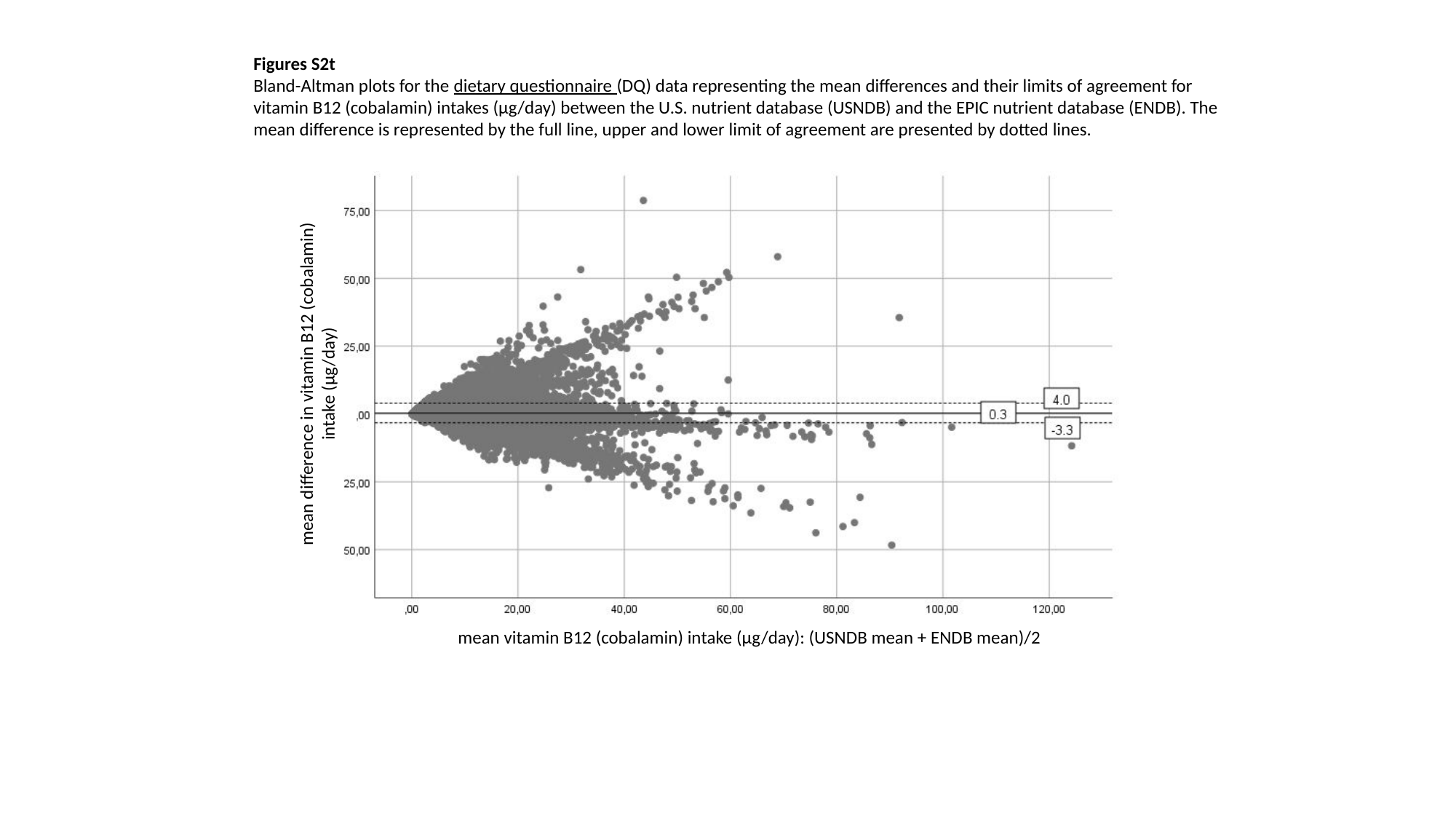

Figures S2t
Bland-Altman plots for the dietary questionnaire (DQ) data representing the mean differences and their limits of agreement for vitamin B12 (cobalamin) intakes (µg/day) between the U.S. nutrient database (USNDB) and the EPIC nutrient database (ENDB). The mean difference is represented by the full line, upper and lower limit of agreement are presented by dotted lines.
mean difference in vitamin B12 (cobalamin) intake (µg/day)
mean vitamin B12 (cobalamin) intake (µg/day): (USNDB mean + ENDB mean)/2

## Slide 21
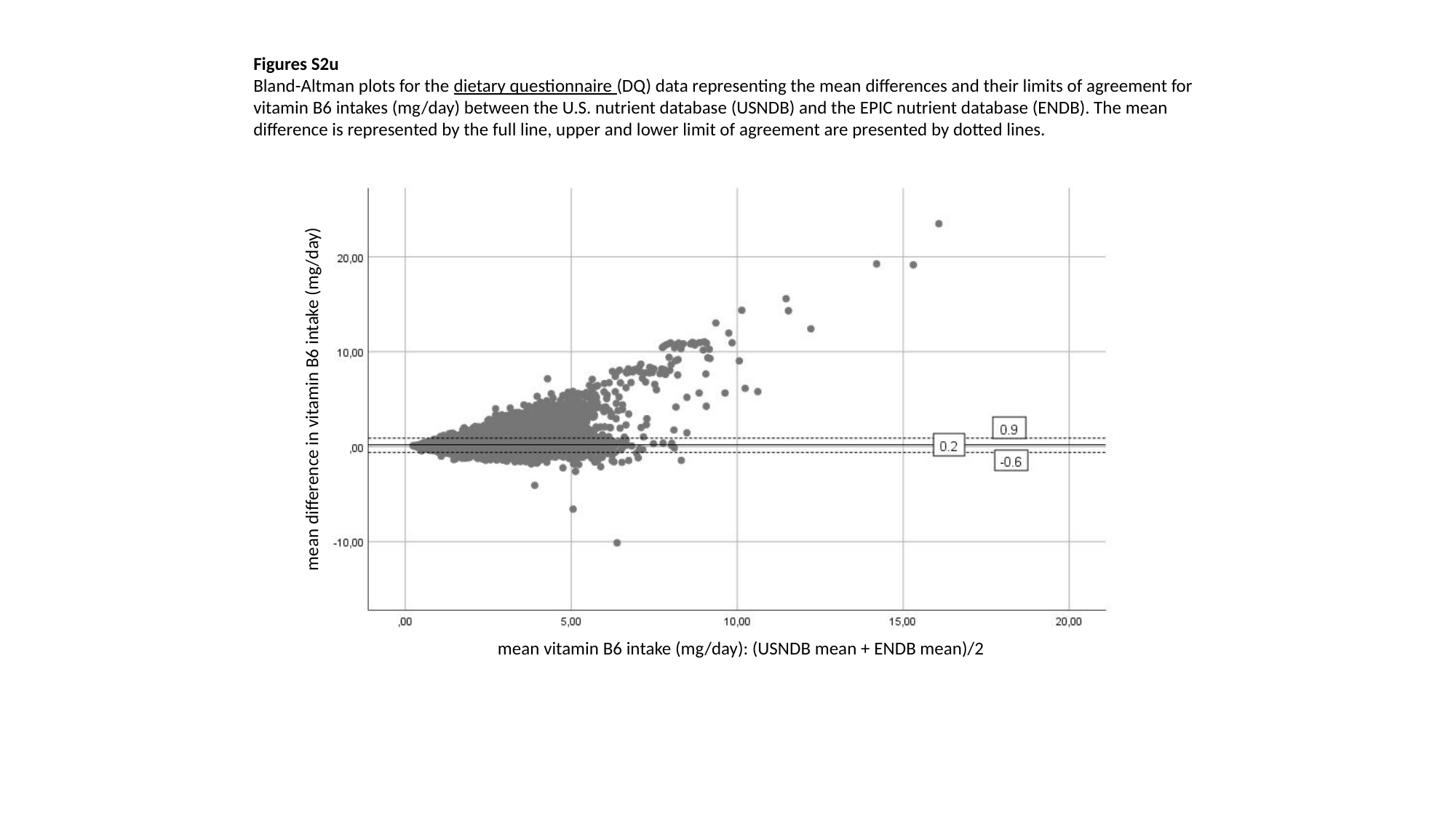

Figures S2u
Bland-Altman plots for the dietary questionnaire (DQ) data representing the mean differences and their limits of agreement for vitamin B6 intakes (mg/day) between the U.S. nutrient database (USNDB) and the EPIC nutrient database (ENDB). The mean difference is represented by the full line, upper and lower limit of agreement are presented by dotted lines.
mean difference in vitamin B6 intake (mg/day)
mean vitamin B6 intake (mg/day): (USNDB mean + ENDB mean)/2

## Slide 22
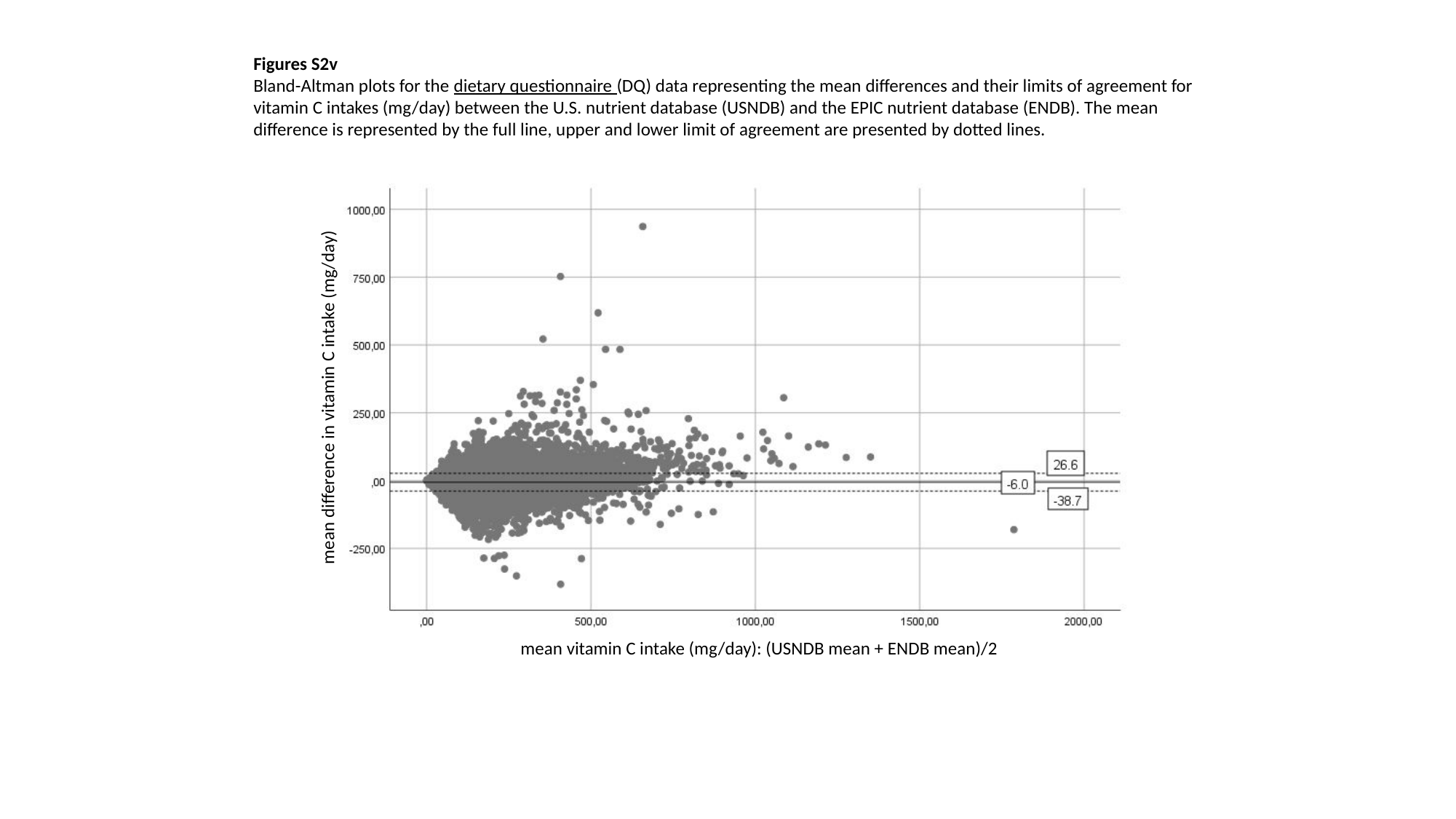

Figures S2v
Bland-Altman plots for the dietary questionnaire (DQ) data representing the mean differences and their limits of agreement for vitamin C intakes (mg/day) between the U.S. nutrient database (USNDB) and the EPIC nutrient database (ENDB). The mean difference is represented by the full line, upper and lower limit of agreement are presented by dotted lines.
mean difference in vitamin C intake (mg/day)
mean vitamin C intake (mg/day): (USNDB mean + ENDB mean)/2

## Slide 23
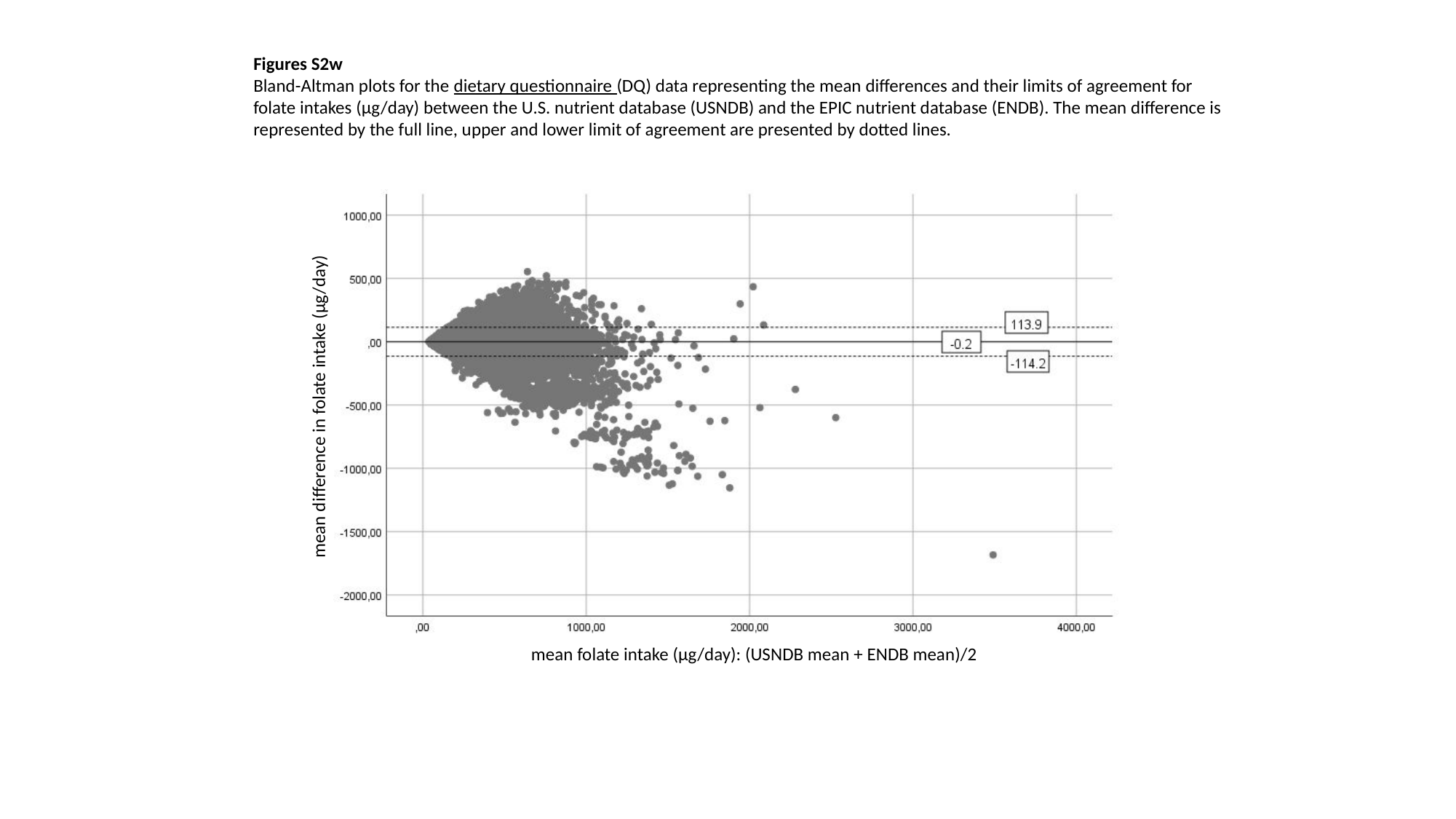

Figures S2w
Bland-Altman plots for the dietary questionnaire (DQ) data representing the mean differences and their limits of agreement for folate intakes (µg/day) between the U.S. nutrient database (USNDB) and the EPIC nutrient database (ENDB). The mean difference is represented by the full line, upper and lower limit of agreement are presented by dotted lines.
mean difference in folate intake (µg/day)
mean folate intake (µg/day): (USNDB mean + ENDB mean)/2
