# Supplementary material for: Comparing Calculated Nutrient Intakes Using Different Food Composition Databases: Results from the European Prospective Investigation into Cancer and Nutrition (EPIC) Cohort
Source: Nutrients. 2020 Sep 23;12(10):2906. doi: 10.3390/nu12102906 (PMC7650652; doi:10.3390/nu12102906)
Supplement: Supplementary file 1 [file nutrients-12-02906-s001.zip › Revision2_Nutrients_TableS1.docx]

**Comparing calculated nutrient intakes using different food composition databases: Results from the European prospective investigation into cancer and nutrition (EPIC) cohort**

Heleen Van Puyvelde, Aurora Perez-Cornago, Corinne Casagrande, Genevieve Nicolas, Vickà Versele, Guri Skeie, Matthias B, Schulze, Ingegerd Johansson, José María Huerta, Oliverio Andreina, Fulvio Ricceri, Jytte Halkjær, Pilar Amiano Etxezarreta, Koen Van Herck, Elisabete Weiderpass, Marc J, Gunter, Inge Huybrechts, on behalf of the EPIC Consortium

**Table 1S**: Mean, standard deviation and median of dietary intakes of 28 nutrients of the U.S. nutrient database (USNDB) and the EPIC nutrient database (ENDB) and their absolute mean difference in nutrient intake, reported for the 24-hour dietary recall data (24-HDR) and the dietary questionnaire data (DQ) by country.

| **Table 1Sa:** Mean, standard deviation and median of dietary intakes of 28 nutrients of the U.S. nutrient database (USNDB) and the EPIC nutrient database (ENDB) and their absolute mean difference in nutrient intake, reported for the 24-hour dietary recall data (24-HDR) and the dietary questionnaire data (DQ) for France | | | | | | | | | |
| --- | --- | --- | --- | --- | --- | --- | --- | --- | --- |
| **Food component** | **Database** | **24-HDR (N = 4,735)** | | | | **DQ (N = 73,035)** | | | |
|  |  | **Mean** | **Standard deviation** | **Median** | **Mean difference*** | **Mean** | **Standard deviation** | **Median** | **Mean difference*** |
| Energy (kcal/day) | ENDB | 1963.4 | ± 651.6 | 1875.2 |  | 2151.6 | ± 576.2 | 2098.3 |  |
|  | USNDB | 1933.4 | ± 613.2 | 1858.1 | -30.0 | 2174.5 | ± 574.0 | 2121.7 | 22.9 |
| Water (g/day) | ENDB | 2484.1 | ± 744.6 | 2394.2 |  | 2643.6 | ± 789.1 | 2535.3 |  |
|  | USNDB | 2461.7 | ± 741.0 | 2370.1 | -22.3 | 2600.6 | ± 783.3 | 2491.0 | -43.0 |
| Total fats (g/day) | ENDB | 82.8 | ± 38.4 | 76.5 |  | 87.7 | ± 27.7 | 84.6 |  |
|  | USNDB | 80.6 | ± 35.5 | 75.2 | -2.2 | 88.4 | ± 28.4 | 85.0 | 0.7 |
| Fatty acids, total saturated (g/day) | ENDB | 35.2 | ± 17.9 | 31.9 |  | 35.8 | ± 13.3 | 34.0 |  |
|  | USNDB | 33.1 | ± 16.4 | 30.3 | -2.1 | 35.1 | ± 13.6 | 33.3 | -0.7 |
| Fatty acids, total monounsaturated (g/day) | ENDB | 27.8 | ± 14.5 | 25.1 |  | 29.2 | ± 9.6 | 28.1 |  |
|  | USNDB | 28.2 | ± 13.8 | 25.8 | 0.4 | 30.9 | ± 9.9 | 29.9 | 1.7 |
| Fatty acids, total polyunsaturated (g/day) | ENDB | 11.7 | ± 8.1 | 9.8 |  | 14.7 | ± 5.7 | 13.9 |  |
|  | USNDB | 12.9 | ± 7.7 | 11.3 | 1.2 | 15.1 | ± 5.2 | 14.4 | 0.4 |
| Cholesterol (mg/day) | ENDB | 331.5 | ± 224.2 | 283.8 |  | 377.6 | ± 151.2 | 356.9 |  |
|  | USNDB | 300.3 | ±178.2 | 261.2 | -31.2 | 354.3 | ± 138.4 | 335.8 | -23.3 |
| Total proteins (g/day) | ENDB | 83.6 | ± 28.5 | 80.2 |  | 94.1 | ± 27.2 | 91.7 |  |
|  | USNDB | 78.2 | ± 27.5 | 74.7 | -5.4 | 92.2 | ± 26.8 | 89.4 | -1.9 |
| Carbohydrates (g/day) | ENDB | 201.5 | ± 74.4 | 192.6 |  | 226.2 | ± 72.9 | 219.0 |  |
|  | USNDB | 209.1 | ± 73.5 | 200.1 | 7.6 | 238.0 | ± 71.2 | 231.0 | 11.8 |
| Sugar, total (g/day) | ENDB | 97.3 | ± 42.1 | 91.5 |  | 103.1 | ± 37.6 | 98.7 |  |
|  | USNDB | 87.7 | ± 39.0 | 81.7 | -9.6 | 93.8 | ± 34.7 | 89.6 | -9.2 |
| Starch (g/day) | ENDB | 102.5 | ± 50.2 | 95.2 |  | 121.4 | ± 51.3 | 114.8 |  |
|  | USNDB | 46.6 | ± 33.6 | 40.5 | -55.8 | 66.6 | ± 34.1 | 61.9 | -54.8 |
| Dietary fiber, total (g/day) | ENDB | 20.1 | ± 8.8 | 18.8 |  | 22.6 | ± 7.0 | 21.9 |  |
|  | USNDB | 20.2 | ± 10.1 | 18.2 | 0.1 | 24.2 | ± 7.8 | 23.4 | 1.6 |
| Alcohol (g/day) | ENDB | 11.1 | ± 16.7 | 4.3 |  | 11.6 | ± 14.7 | 6.4 |  |
|  | USNDB | 12.3 | ± 19.0 | 4.3 | 1.1 | 13.1 | ± 16.4 | 7.4 | 1.5 |
| Calcium, Ca (mg/day) | ENDB | 907.4 | ± 400.7 | 842.6 |  | 1055.6 | ± 437.6 | 979.6 |  |
|  | USNDB | 1081.3 | ± 535.8 | 973.9 | 173.9 | 1352.0 | ± 558.7 | 1255.8 | 296.4 |
| Iron, Fe (mg/day) | ENDB | 12.6 | ± 5.6 | 11.6 |  | 14.1 | ± 4.0 | 13.7 |  |
|  | USNDB | 11.8 | ± 5.3 | 10.8 | -0.8 | 14.8 | ± 4.8 | 14.2 | 0.7 |
| Potassium, K (mg/day) | ENDB | 3287.5 | ± 1050.8 | 3191.8 |  | 3704.0 | ± 983.5 | 3601.1 |  |
|  | USNDB | 2995.0 | ± 969.9 | 2905.4 | -292.5 | 3451.6 | ± 944.7 | 3342.8 | -252.4 |
| Magnesium, Mg (mg/day) | ENDB | 368.0 | ± 135.9 | 349.4 |  | 425.8 | ± 144.0 | 405.0 |  |
|  | USNDB | 299.6 | ± 107.5 | 281.5 | -68.3 | 350.5 | ± 98.3 | 337.6 | -75.4 |
| Phosphorus, P (mg/day) | ENDB | 1269.2 | ± 421.0 | 1215.5 |  | 1459.2 | ± 450.6 | 1402.4 |  |
|  | USNDB | 1340.4 | ± 483.1 | 1265.5 | 71.2 | 1615.2 | ± 511.2 | 1546.8 | 156.0 |
| Vitamin D (µg/day) | ENDB | 2.7 | ± 4.5 | 1.4 |  | 2.8 | ± 1.3 | 2.6 |  |
|  | USNDB | 2.2 | ± 2.5 | 1.6 | -0.4 | 2.8 | ± 1.3 | 2.6 | 0.0 |
| Vitamin E (alpha-tocopherol) (mg/day) | ENDB | 11.0 | ± 7.7 | 9.1 |  | 13.5 | ± 5.4 | 2.7 |  |
|  | USNDB | 8.1 | ± 4.7 | 7.1 | -2.9 | 9.6 | ± 3.3 | 9.2 | -3.9 |
| Retinol (µg/day) | ENDB | 640.3 | ± 1429.9 | 362.1 |  | 880.4 | ± 741.9 | 662.5 |  |
|  | USNDB | 638.2 | ± 1316.6 | 397.9 | -2.1 | 864.7 | ± 642.8 | 690.6 | -15.8 |
| Beta-carotene (µg/day) | ENDB | 4282.5 | ± 4352.2 | 2888.7 |  | 4982.4 | ± 2217.9 | 4687.4 |  |
|  | USNDB | 4131.6 | ± 4286.6 | 2846.0 | -150.9 | 6157.4 | ± 2671.6 | 5825.1 | 1174.9 |
| Thiamin, B1 (mg/day) | ENDB | 1.2 | ± 0.6 | 1.1 |  | 1.3 | ± 0.4 | 1.3 |  |
|  | USNDB | 1.4 | ± 0.6 | 1.3 | 0.2 | 1.7 | ± 0.6 | 1.7 | 0.4 |
| Riboflavin, B2 (mg/day) | ENDB | 1.9 | ± 0.8 | 1.8 |  | 2.3 | ± 0.8 | 2.2 |  |
|  | USNDB | 2.0 | ± 0.8 | 1.8 | 0.1 | 2.4 | ± 0.8 | 2.3 | 0.1 |
| Cobalamin, B12 (µg/day) | ENDB | 6.3 | ± 9.8 | 4.2 |  | 8.2 | ± 5.5 | 6.7 |  |
|  | USNDB | 7.1 | ± 10.1 | 4.8 | 0.7 | 9.4 | ± 5.4 | 8.1 | 1.2 |
| Vitamin B6 (mg/day) | ENDB | 1.6 | ± 0.6 | 1.5 |  | 1.8 | ± 0.5 | 1.8 |  |
|  | USNDB | 1.8 | ± 0.8 | 1.7 | 0.2 | 2.0 | ± 0.6 | 1.9 | 0.1 |
| Vitamin C (mg/day) | ENDB | 113.2 | ± 75.2 | 99.7 |  | 140.0 | ± 60.1 | 131.3 |  |
|  | USNDB | 107.5 | ± 78.2 | 92.2 | -5.7 | 133.4 | ± 63.9 | 123.1 | -6.5 |
| Folate, food (µg/day) | ENDB | 291.3 | ± 142.6 | 263.6 |  | 355.1 | ± 106.2 | 342.7 |  |
|  | USNDB | 278.7 | ± 141.8 | 250.0 | -12.6 | 336.8 | ± 97.5 | 326.4 | -18.3 |
| * Absolute differences in mean nutrient intake between the USNDB and ENDB were statistically significant (paired samples t-test: p < 0.001) for all nutrients | | | | | | | | | |

| **Table 1Sb:** Mean, standard deviation and median of dietary intakes of 28 nutrients of the U.S. nutrient database (USNDB) and the EPIC nutrient database (ENDB) and their absolute mean difference in nutrient intake, reported for the 24-hour dietary recall data (24-HDR) and the dietary questionnaire data (DQ) for Italy | | | | | | | | | |
| --- | --- | --- | --- | --- | --- | --- | --- | --- | --- |
| **Food component** | **Database** | **24-HDR (N = 3,961)** | | | | **DQ (N = 45,908)** | | | |
|  |  | **Mean** | **Standard deviation** | **Median** | **Mean difference*** | **Mean** | **Standard deviation** | **Median** | **Mean difference*** |
| Energy (kcal/day) | ENDB | 2098.8 | ± 812.7 | 1977.8 |  | 2305.4 | ± 662.7 | 2235.5 |  |
|  | USNDB | 2069.2 | ± 784.9 | 1949.8 | -29.6 | 2297.8 | ± 657.9 | 2230.1 | -7.6 |
| Water (g/day) | ENDB | 2109.1 | ± 777.4 | 1991.6 |  | 1349.6 | ± 410.2 | 1294.7 |  |
|  | USNDB | 2113.6 | ± 776.6 | 1998.4 | 4.5 | 1371.4 | ± 412.7 | 1316.2 | 21.8 |
| Total fats (g/day) | ENDB | 72.7 | ± 36.0 | 66.3 |  | 86.9 | ± 27.8 | 83.5 |  |
|  | USNDB | 73.3 | ± 34.9 | 67.5 | 0.6 | 89.4 | ± 27.9 | 86.0 | 2.6 |
| Fatty acids, total saturated (g/day) | ENDB | 24.3 | ± 13.2 | 21.8 |  | 30.2 | ± 11.2 | 28.6 |  |
|  | USNDB | 23.3 | ± 12.1 | 21.2 | -0.9 | 29.8 | ± 10.9 | 28.3 | -0.3 |
| Fatty acids, total monounsaturated (g/day) | ENDB | 34.1 | ± 18.8 | 30.5 |  | 40.6 | ± 13.5 | 38.8 |  |
|  | USNDB | 33.2 | ± 18.3 | 29.7 | -0.8 | 39.9 | ± 13.2 | 38.2 | -0.7 |
| Fatty acids, total polyunsaturated (g/day) | ENDB | 9.9 | ± 6.6 | 8.3 |  | 11.0 | ± 4.2 | 10.3 |  |
|  | USNDB | 11.2 | ± 7.2 | 9.5 | 1.3 | 13.1 | ± 4.7 | 12.4 | 2.1 |
| Cholesterol (mg/day) | ENDB | 305.3 | ± 232.4 | 243.2 |  | 368.5 | ± 134.2 | 352.5 |  |
|  | USNDB | 221.3 | ± 154.3 | 185.3 | -84.0 | 288.7 | ± 103.4 | 276.2 | -79.9 |
| Total proteins (g/day) | ENDB | 85.3 | ± 35.8 | 79.7 |  | 96.6 | ± 28.1 | 93.8 |  |
|  | USNDB | 76.1 | ± 30.3 | 71.5 | -9.3 | 89.2 | ± 26.1 | 86.6 | -7.4 |
| Carbohydrates (g/day) | ENDB | 249.1 | ± 103.1 | 235.4 |  | 260.6 | ± 88.2 | 250.2 |  |
|  | USNDB | 254.4 | ± 99.9 | 241.2 | 5.3 | 265.4 | ± 85.4 | 256.0 | 4.8 |
| Sugar, total (g/day) | ENDB | 94.4 | ± 45.5 | 86.8 |  | 99.8 | ± 39.9 | 93.5 |  |
|  | USNDB | 87.9 | ± 43.1 | 81.4 | -6.4 | 98.5 | ± 38.3 | 92.7 | -1.3 |
| Starch (g/day) | ENDB | 154.7 | ± 80.3 | 143.6 |  | 160.7 | ± 69.3 | 150.3 |  |
|  | USNDB | 61.6 | ± 48.7 | 52.7 | -93.1 | 74.6 | ± 42.7 | 66.6 | -86.2 |
| Dietary fiber, total (g/day) | ENDB | 21.8 | ± 10.0 | 20.2 |  | 22.3 | ± 7.6 | 21.3 |  |
|  | USNDB | 21.2 | ± 10.1 | 19.4 | -0.6 | 22.1 | ± 7.9 | 21.0 | -0.2 |
| Alcohol (g/day) | ENDB | 15.2 | ± 22.3 | 6.0 |  | 13.6 | ± 17.7 | 5.9 |  |
|  | USNDB | 16.5 | ± 24.4 | 6.2 | 1.4 | 15.0 | ± 19.6 | 6.4 | 1.4 |
| Calcium, Ca (mg/day) | ENDB | 815.3 | ± 439.5 | 735.6 |  | 1027.1 | ± 398.8 | 967.7 |  |
|  | USNDB | 942.8 | ± 464.8 | 866.5 | 127.5 | 1139.0 | ± 417.4 | 1087.6 | 111.9 |
| Iron, Fe (mg/day) | ENDB | 12.8 | ± 6.6 | 11.7 |  | 13.6 | ± 4.0 | 13.2 |  |
|  | USNDB | 12.4 | ± 5.9 | 11.5 | -0.4 | 13.9 | ± 4.7 | 13.3 | 0.3 |
| Potassium, K (mg/day) | ENDB | 3489.0 | ± 1348.7 | 3289.2 |  | 3394.3 | ± 936.7 | 3292.3 |  |
|  | USNDB | 2687.8 | ± 1092.0 | 2528.6 | -801.1 | 2874.9 | ± 821.5 | 2783.3 | -519.4 |
| Magnesium, Mg (mg/day) | ENDB | 315.5 | ± 122.7 | 297.6 |  | 302.3 | ± 96.6 | 287.9 |  |
|  | USNDB | 298.4 | ± 118.6 | 282.0 | -17.1 | 310.2 | ± 90.4 | 300.4 | 7.8 |
| Phosphorus, P (mg/day) | ENDB | 1303.9 | ± 503.8 | 1233.0 |  | 1491.2 | ± 423.5 | 1449.3 |  |
|  | USNDB | 1194.5 | ± 454.0 | 1137.1 | -109.3 | 1401.4 | ± 408.4 | 1360.3 | -89.9 |
| Vitamin D (µg/day) | ENDB | 2.1 | ± 3.6 | 1.0 |  | 2.7 | ± 1.4 | 2.5 |  |
|  | USNDB | 1.8 | ± 2.7 | 1.0 | -0.3 | 2.4 | ± 1.2 | 2.1 | -0.4 |
| Vitamin E (alpha-tocopherol) (mg/day) | ENDB | 11.2 | ± 6.4 | 10.0 |  | 12.1 | ± 4.1 | 11.5 |  |
|  | USNDB | 8.1 | ± 4.9 | 7.0 | -3.1 | 9.5 | ± 3.4 | 9.0 | -2.6 |
| Retinol (µg/day) | ENDB | 448.2 | ± 2190.2 | 194.7 |  | 733.1 | ± 748.9 | 466.3 |  |
|  | USNDB | 357.4 | ± 1217.2 | 212.1 | -90.8 | 471.9 | ± 310.4 | 393.5 | -261.2 |
| Beta-carotene (µg/day) | ENDB | 2545.1 | ± 2632.9 | 1675.3 |  | 2809.0 | ± 1613.0 | 2433.0 |  |
|  | USNDB | 3251.9 | ± 3867.1 | 1955.8 | 706.8 | 3743.4 | ± 2391.4 | 3170.9 | 934.3 |
| Thiamin, B1 (mg/day) | ENDB | 0.9 | ± 0.4 | 0.8 |  | 0.9 | ± 0.3 | 0.9 |  |
|  | USNDB | 1.6 | ± 0.8 | 1.5 | 0.7 | 1.7 | ± 0.6 | 1.6 | 0.8 |
| Riboflavin, B2 (mg/day) | ENDB | 1.5 | ± 0.7 | 1.3 |  | 1.6 | ± 0.5 | 1.5 |  |
|  | USNDB | 1.8 | ± 0.8 | 1.7 | 0.3 | 1.9 | ± 0.6 | 1.9 | 0.3 |
| Cobalamin, B12 (µg/day) | ENDB | 6.3 | ± 14.2 | 3.7 |  | 7.6 | ± 3.7 | 6.9 |  |
|  | USNDB | 5.4 | ± 9.6 | 3.4 | -0.9 | 6.7 | ± 3.2 | 6.1 | -0.9 |
| Vitamin B6 (mg/day) | ENDB | 1.7 | ± 0.8 | 1.6 |  | 1.7 | ± 0.5 | 1.6 |  |
|  | USNDB | 1.7 | ± 0.8 | 1.5 | 0.0 | 1.7 | ± 0.5 | 1.6 | 0.0 |
| Vitamin C (mg/day) | ENDB | 123.4 | ± 96.2 | 97.9 |  | 127.1 | ± 68.0 | 114.4 |  |
|  | USNDB | 116.4 | ± 106.7 | 81.9 | -7.1 | 119.5 | ± 69.0 | 105.7 | -7.6 |
| Folate, food (µg/day) | ENDB | 261.4 | ± 150.2 | 233.2 |  | 257.0 (85) | ± 85.0 | 246.0 |  |
|  | USNDB | 259.0 | ± 152.6 | 227.3 | -2.4 | 266.2 (94.6) | ± 94.6 | 250.4 | 9.2 |
| * Absolute differences in mean nutrient intake between the USNDB and ENDB were statistically significant (paired samples t-test: p < 0.001) for all nutrients | | | | | | | | | |

| **Table 1Sc:** Mean, standard deviation and median of dietary intakes of 28 nutrients of the U.S. nutrient database (USNDB) and the EPIC nutrient database (ENDB) and their absolute mean difference in nutrient intake, reported for the 24-hour dietary recall data (24-HDR) and the dietary questionnaire data (DQ) for Spain | | | | | | | | | |
| --- | --- | --- | --- | --- | --- | --- | --- | --- | --- |
| **Food component** | **Database** | **24-HDR (N = 3,220)** | | | | **DQ (N = 40,621)** | | | |
|  |  | **Mean** | **Standard deviation** | **Median** | **Mean difference*** | **Mean** | **Standard deviation** | **Median** | **Mean difference*** |
| Energy (kcal/day) | ENDB | 2319.7 | ± 887.4 | 2196.0 |  | 2128.0 | ± 663.8 | 2040.2 |  |
|  | USNDB | 2330.0 | ± 885.0 | 2210.8 | 10.3 | 2220.3 | ± 689.4 | 2125.9 | 92.3 |
| Water (g/day) | ENDB | 2475.8 | ± 959.1 | 2299.9 |  | 1501.3 | ± 438.2 | 1445.2 |  |
|  | USNDB | 2466.1 | ± 955.5 | 2288.7 | -9.7 | 1494.7 | ± 434.4 | 1438.9 | -6.6 |
| Total fats (g/day) | ENDB | 96.7 | ± 46.3 | 89.2 |  | 84.3 | ± 31.2 | 79.9 |  |
|  | USNDB | 93.9 | ± 44.9 | 86.6 | -2.8 | 85.6 | ± 31.2 | 81.1 | 1.3 |
| Fatty acids, total saturated (g/day) | ENDB | 28.3 | ± 15.4 | 25.4 |  | 26.3 | ± 11.9 | 24.4 |  |
|  | USNDB | 25.6 | ± 14.2 | 22.9 | -2.7 | 24.6 | ± 11.0 | 22.7 | -1.8 |
| Fatty acids, total monounsaturated (g/day) | ENDB | 45.2 | ± 24.2 | 40.8 |  | 37.3 | ± 15.2 | 35.1 |  |
|  | USNDB | 43.9 | ± 23.9 | 39.1 | -1.3 | 37.2 | ± 15.3 | 34.9 | -0.1 |
| Fatty acids, total polyunsaturated (g/day) | ENDB | 15.6 | ± 11.4 | 12.5 |  | 13.7 | ± 7.6 | 11.7 |  |
|  | USNDB | 18.0 | ± 12.1 | 14.8 | 2.4 | 17.4 | ± 8.8 | 15.4 | 3.7 |
| Cholesterol (mg/day) | ENDB | 441.2 | ± 286.2 | 383.0 |  | 379.9 | ± 152.2 | 361.4 |  |
|  | USNDB | 346.9 | ± 226.8 | 298.9 | -94.2 | 315.1 | ± 123.0 | 300.0 | -64.8 |
| Total proteins (g/day) | ENDB | 108.9 | ± 46.4 | 101.2 |  | 101.9 | ± 30.7 | 98.1 |  |
|  | USNDB | 96.6 | ± 39.6 | 89.9 | -12.3 | 94.8 | ± 28.4 | 91.2 | -7.1 |
| Carbohydrates (g/day) | ENDB | 219.5 | ± 89.0 | 205.6 |  | 217.0 | ± 68.3 | 209.6 |  |
|  | USNDB | 247.3 | ± 99.6 | 233.6 | 27.8 | 250.5 | ± 76.0 | 242.0 | 33.5 |
| Sugar, total (g/day) | ENDB | 105.4 | ± 51.0 | 97.0 |  | 88.9 | ± 34.8 | 84.2 |  |
|  | USNDB | 105.2 | ± 49.0 | 97.0 | -0.2 | 91.8 | ± 34.7 | 87.1 | 3.0 |
| Starch (g/day) | ENDB | 114.2 | ± 64.6 | 103.5 |  | 128.1 | ± 52.5 | 122.2 |  |
|  | USNDB | 54.9 | ± 42.8 | 44.3 | -59.3 | 71.5 | ± 37.1 | 66.1 | -56.6 |
| Dietary fiber, total (g/day) | ENDB | 23.7 | ± 12.3 | 21.5 |  | 24.5 | ± 8.3 | 23.5 |  |
|  | USNDB | 24.6 | ± 13.4 | 22.1 | 1.0 | 27.2 | ± 9.3 | 26.1 | 2.7 |
| Alcohol (g/day) | ENDB | 19.3 | ± 30.1 | 6.0 |  | 13.4 | ± 22.4 | 2.3 |  |
|  | USNDB | 20.6 | ± 32.9 | 5.7 | 1.3 | 14.2 | ± 24.2 | 2.3 | 0.8 |
| Calcium, Ca (mg/day) | ENDB | 980.9 | ± 403.6 | 913.1 |  | 903.2 | ± 415.7 | 829.8 |  |
|  | USNDB | 1042.2 | ± 435.6 | 971.7 | 61.3 | 1032.0 | ± 421.5 | 966.3 | 128.8 |
| Iron, Fe (mg/day) | ENDB | 16.1 | ± 7.5 | 14.7 |  | 15.4 | ± 5.4 | 14.5 |  |
|  | USNDB | 13.8 | ± 7.0 | 12.4 | -2.3 | 14.5 | ± 5.6 | 13.6 | -0.8 |
| Potassium, K (mg/day) | ENDB | 4059.5 | ± 1520.2 | 3829.6 |  | 3716.3 | ± 1040.2 | 3601.6 |  |
|  | USNDB | 3498.3 | ± 1283.9 | 3348.9 | -561.1 | 3361.8 | ± 966.4 | 3250.3 | -354.5 |
| Magnesium, Mg (mg/day) | ENDB | 390.7 | ± 138.3 | 367.7 |  | 337.9 | ± 103.1 | 322.7 |  |
|  | USNDB | 367.2 | ± 142.3 | 343.7 | -23.5 | 355.9 | ± 109.5 | 340.7 | 18.0 |
| Phosphorus, P (mg/day) | ENDB | 1554.6 | ± 576.5 | 1463.6 |  | 1487.2 | ± 454.8 | 1432.0 |  |
|  | USNDB | 1553.0 | ± 562.3 | 1467.7 | -1.6 | 1523.4 | ± 449.9 | 1468.5 | 36.2 |
| Vitamin D (µg/day) | ENDB | 5.0 | ± 8.0 | 2.0 |  | 4.1 | ± 2.8 | 3.4 |  |
|  | USNDB | 3.8 | ± 4.7 | 2.7 | -1.2 | 3.4 | ± 2.0 | 3.0 | -0.7 |
| Vitamin E (alpha-tocopherol) (mg/day) | ENDB | 15.2 | ± 9.9 | 12.6 |  | 13.4 | ± 6.6 | 11.8 |  |
|  | USNDB | 12.7 | ± 7.8 | 10.9 | -2.6 | 11.9 | ± 5.7 | 10.8 | -1.4 |
| Retinol (µg/day) | ENDB | 493.5 | ± 1602.4 | 252.2 |  | 491.1 | ± 750.4 | 279.4 |  |
|  | USNDB | 493.8 | ± 1959.3 | 253.0 | 0.3 | 446.3 | ± 543.3 | 284.9 | -44.9 |
| Beta-carotene (µg/day) | ENDB | 2415.3 | ± 2617.9 | 1529.3 |  | 2459.2 | ± 1566.7 | 2078.5 |  |
|  | USNDB | 3241.2 | ± 3347.7 | 2283.9 | 825.9 | 4669.3 | ± 3129.0 | 4007.2 | 2210.1 |
| Thiamin, B1 (mg/day) | ENDB | 1.5 | ± 0.7 | 1.4 |  | 1.5 | ± 0.5 | 1.4 |  |
|  | USNDB | 1.8 | ± 0.8 | 1.6 | 0.3 | 1.8 | ± 0.6 | 1.7 | 0.3 |
| Riboflavin, B2 (mg/day) | ENDB | 1.8 | ± 0.9 | 1.7 |  | 1.7 | ± 0.6 | 1.6 |  |
|  | USNDB | 2.3 | ± 1.0 | 2.1 | 0.4 | 2.1 | ± 0.7 | 2.0 | 0.4 |
| Cobalamin, B12 (µg/day) | ENDB | 8.3 | ± 14.1 | 5.3 |  | 6.9 | ± 4.2 | 5.8 |  |
|  | USNDB | 9.0 | ± 14.8 | 5.9 | 0.7 | 8.4 | ± 5.3 | 7.2 | 1.5 |
| Vitamin B6 (mg/day) | ENDB | 2.2 | ± 1.0 | 2.0 |  | 2.0 | ± 0.6 | 2.0 |  |
|  | USNDB | 2.4 | ± 1.2 | 2.2 | 0.2 | 2.3 | ± 0.8 | 2.2 | 0.3 |
| Vitamin C (mg/day) | ENDB | 147.6 | ± 119.8 | 118.8 |  | 148.2 | ± 80.3 | 134.8 |  |
|  | USNDB | 145.6 | ± 131.8 | 110.1 | -2.0 | 147.4 | ± 87.7 | 131.3 | -0.8 |
| Folate, food (µg/day) | ENDB | 296.1 | ± 160.3 | 266.1 |  | 312.8 | ± 111.8 | 298.0 |  |
|  | USNDB | 364.6 | ± 203.2 | 317.5 | 68.5 | 387.2 | ± 387.7 | 369.5 | 74.5 |
| * Absolute differences in mean nutrient intake between the USNDB and ENDB were statistically significant (paired samples t-test: p < 0.001) for all nutrients | | | | | | | | | |

| **Table 1Sd:** Mean, standard deviation and median of dietary intakes of 28 nutrients of the U.S. nutrient database (USNDB) and the EPIC nutrient database (ENDB) and their absolute mean difference in nutrient intake, reported for the 24-hour dietary recall data (24-HDR) and the dietary questionnaire data (DQ) for United Kingdom | | | | | | | | | |
| --- | --- | --- | --- | --- | --- | --- | --- | --- | --- |
| **Food component** | **Database** | **24-HDR (N = 1,315)** | | | | **DQ (N = 81,097)** | | | |
|  |  | **Mean** | **Standard deviation** | **Median** | **Mean difference*** | **Mean** | **Standard deviation** | **Median** | **Mean difference*** |
| Energy (kcal/day) | ENDB | 1990.1 | ± 694.9 | 1911.2 |  | 1984.1 | ± 557.5 | 1915.7 |  |
|  | USNDB | 2045.1 | ± 700.8 | 1957.9 | 55.0 | 2095.6 | ± 583.2 | 2028.5 | 111.5 |
| Water (g/day) | ENDB | 2730.9 | ± 870.5 | 2599.1 |  | 2366.1 | ± 657.0 | 2330.0 |  |
|  | USNDB | 2721.8 | ± 867.8 | 2576.2 | -9.1 | 2356.2 | ± 653.5 | 2322.2 | -9.9 |
| Total fats (g/day) | ENDB | 73.9 | ± 37.1 | 66.9 |  | 73.8 | ± 27.8 | 70.0 |  |
|  | USNDB | 74.0 | ± 37.3 | 67.9 | 0.0 | 73.8 | ± 28.4 | 70.0 | 0.0 |
| Fatty acids, total saturated (g/day) | ENDB | 28.3 | ± 16.4 | 25.5 |  | 28.3 | ± 12.3 | 26.3 |  |
|  | USNDB | 24.1 | ± 14.4 | 21.3 | -4.1 | 24.7 | ± 11.0 | 23.0 | -3.6 |
| Fatty acids, total monounsaturated (g/day) | ENDB | 26.4 | ± 14.6 | 23.6 |  | 24.5 | ± 9.8 | 23.1 |  |
|  | USNDB | 26.7 | ± 15.1 | 23.8 | 0.3 | 24.8 | ± 10.2 | 23.4 | 0.3 |
| Fatty acids, total polyunsaturated (g/day) | ENDB | 13.5 | ± 9.0 | 11.4 |  | 15.3 | ± 6.8 | 13.9 |  |
|  | USNDB | 16.9 | ± 10.2 | 15.0 | 3.4 | 17.6 | ± 7.3 | 16.4 | 2.3 |
| Cholesterol (mg/day) | ENDB | 242.2 | ± 179.6 | 206.3 |  | 248.7 | ± 128.0 | 235.1 |  |
|  | USNDB | 183.6 | ± 148.4 | 150.0 | -58.6 | 192.5 | ± 95.2 | 183.5 | -56.2 |
| Total proteins (g/day) | ENDB | 74.9 | ± 28.7 | 70.6 |  | 80.5 | ± 24.3 | 78.3 |  |
|  | USNDB | 67.7 | ± 24.4 | 64.2 | -7.2 | 74.3 | ± 20.9 | 72.5 | -6.2 |
| Carbohydrates (g/day) | ENDB | 231.6 | ± 84.6 | 219.9 |  | 232.4 | ± 71.7 | 223.4 |  |
|  | USNDB | 262.2 | ± 93.7 | 250.8 | 30.5 | 279.1 | ± 85.1 | 268.0 | 46.7 |
| Sugar, total (g/day) | ENDB | 114.3 | ± 53.8 | 105.2 |  | 123.8 | ± 46.8 | 117.0 |  |
|  | USNDB | 109.0 | ± 52.4 | 100.5 | -5.3 | 127.2 | ± 50.9 | 118.5 | 3.3 |
| Starch (g/day) | ENDB | 117.1 | ± 52.2 | 109.3 |  | 107.7 | ± 37.5 | 103.4 |  |
|  | USNDB | 51.6 | ± 36.2 | 43.3 | -65.5 | 50.4 | ± 22.8 | 46.9 | -57.4 |
| Dietary fiber, total (g/day) | ENDB | 21.0 | ± 9.8 | 19.3 |  | 24.5 | ± 9.0 | 23.2 |  |
|  | USNDB | 23.9 | ± 11.6 | 21.9 | 2.9 | 28.4 | ± 10.5 | 27.0 | 4.0 |
| Alcohol (g/day) | ENDB | 14.1 | ± 25.3 | 0.0 |  | 9.7 | ± 13.3 | 5.6 |  |
|  | USNDB | 15.0 | ± 25.9 | 0.1 | 0.9 | 10.2 | ± 13.4 | 6.0 | 0.4 |
| Calcium, Ca (mg/day) | ENDB | 967.9 | ± 402.6 | 909.9 |  | 1073.2 | ± 349.9 | 1046.0 |  |
|  | USNDB | 863.3 | ± 350.9 | 809.9 | -104.5 | 1022.4 | ± 337.4 | 995.4 | -50.9 |
| Iron, Fe (mg/day) | ENDB | 12.7 | ± 5.8 | 11.9 |  | 12.5 | ± 3.7 | 12.1 |  |
|  | USNDB | 14.1 | ± 7.7 | 12.6 | 1.4 | 13.9 | ± 4.9 | 13.3 | 1.4 |
| Potassium, K (mg/day) | ENDB | 3585.3 | ± 1114.3 | 3467.9 |  | 4062.6 | ± 1028.3 | 3975.7 |  |
|  | USNDB | 3291.3 | ± 1032.4 | 3200.0 | -293.9 | 3764.2 | ± 961.0 | 3676.5 | -298.4 |
| Magnesium, Mg (mg/day) | ENDB | 331.3 | ± 116.4 | 316.8 |  | 359.6 | ± 96.4 | 349.6 |  |
|  | USNDB | 364.4 | ± 136.1 | 343.4 | 33.1 | 398.8 | ± 114.7 | 385.8 | 39.3 |
| Phosphorus, P (mg/day) | ENDB | 1359.4 | ± 450.4 | 1305.3 |  | 1533.8 | ± 412.8 | 1502.3 |  |
|  | USNDB | 1294.0 | ± 439.3 | 1237.4 | -65.4 | 1430.2 | 388.3 | 1399.1 | -103.6 |
| Vitamin D (µg/day) | ENDB | 3.7 | ± 4.1 | 2.8 |  | 3.8 | ± 2.2 | 3.4 |  |
|  | USNDB | 1.8 | ± 2.6 | 1.1 | -2.0 | 1.8 | ± 1.1 | 1.6 | -2.0 |
| Vitamin E (alpha-tocopherol) (mg/day) | ENDB | 10.9 | ± 7.2 | 9.2 |  | 13.0 | ± 5.9 | 11.5 |  |
|  | USNDB | 9.9 | ± 5.8 | 8.6 | -1.0 | 10.5 | ± 4.1 | 9.9 | -2.4 |
| Retinol (µg/day) | ENDB | 588.2 | ± 1410.1 | 370.1 |  | 649.3 | ± 568.0 | 493.8 |  |
|  | USNDB | 505.7 | ± 642.0 | 407.6 | -82.5 | 545.7 | ± 321.1 | 491.7 | -103.6 |
| Beta-carotene (µg/day) | ENDB | 3280.1 | ± 4312.9 | 1157.7 |  | 4426.9 | ± 3180.9 | 4098.6 |  |
|  | USNDB | 3081.7 | ± 3590.0 | 1607.0 | -198.4 | 4202.1 | ± 2722.8 | 3817.4 | -224.8 |
| Thiamin, B1 (mg/day) | ENDB | 1.7 | ± 0.8 | 1.6 |  | 1.8 | ± 0.6 | 1.8 |  |
|  | USNDB | 1.9 | ± 1.0 | 1.8 | 0.2 | 2.2 | ± 1.0 | 2.0 | 0.3 |
| Riboflavin, B2 (mg/day) | ENDB | 2.0 | ± 0.9 | 1.9 |  | 2.3 | ± 0.8 | 2.3 |  |
|  | USNDB | 2.2 | ± 0.9 | 2.0 | 0.2 | 2.6 | ± 0.9 | 2.4 | 0.2 |
| Cobalamin, B12 (µg/day) | ENDB | 4.7 | ± 5.9 | 3.7 |  | 5.2 | ± 2.8 | 5.0 |  |
|  | USNDB | 5.3 | ± 8.5 | 3.7 | 0.6 | 5.4 | ± 3.2 | 4.9 | 0.1 |
| Vitamin B6 (mg/day) | ENDB | 2.1 | ± 0.9 | 2.0 |  | 2.3 | ± 0.6 | 2.3 |  |
|  | USNDB | 2.3 | ± 1.4 | 1.9 | 0.1 | 2.4 | ± 0.8 | 2.2 | 0.0 |
| Vitamin C (mg/day) | ENDB | 112.7 | ± 83.8 | 93.6 |  | 142.9 | ± 68.3 | 130.9 |  |
|  | USNDB | 101.7 | ± 78.6 | 83.1 | -11.0 | 143.0 | ± 70.9 | 130.3 | 0.1 |
| Folate, food (µg/day) | ENDB | 322.0 | ± 140.1 | 300.1 |  | 406.4 | ± 158.0 | 376.8 |  |
|  | USNDB | 296.5 | ± 131.4 | 271.8 | -25.5 | 364.6 | ± 124.0 | 346.1 | -41.8 |
| * Absolute differences in mean nutrient intake between the USNDB and ENDB were statistically significant (paired samples t-test: p < 0.001) for all nutrients | | | | | | | | | |

| **Table 1Se:** Mean, standard deviation and median of dietary intakes of 28 nutrients of the U.S. nutrient database (USNDB) and the EPIC nutrient database (ENDB) and their absolute mean difference in nutrient intake, reported for the 24-hour dietary recall data (24-HDR) and the dietary questionnaire data (DQ) for The Netherlands | | | | | | | | | |
| --- | --- | --- | --- | --- | --- | --- | --- | --- | --- |
| **Food component** | **Database** | **24-HDR (N = 4,567)** | | | | **DQ (N = 39,036)** | | | |
|  |  | **Mean** | **Standard deviation** | **Median** | **Mean difference*** | **Mean** | **Standard deviation** | **Median** | **Mean difference*** |
| Energy (kcal/day) | ENDB | 2178.1 | ± 843.5 | 2061.4 |  | 2047.4 | ± 591.4 | 1953.1 |  |
|  | USNDB | 2254.3 | ± 843.2 | 2140.0 | 76.2 | 2182.4 | ± 602.3 | 2093.7 | 135.0 |
| Water (g/day) | ENDB | 2702.1 | ± 905.0 | 2581.5 |  | 2531.8 | ± 674.8 | 2454.8 |  |
|  | USNDB | 2689.9 | ± 894.8 | 2571.6 | -12.2 | 2513.7 | ± 668.1 | 2437.2 | -18.1 |
| Total fats (g/day) | ENDB | 85.1 | ± 43.5 | 77.8 |  | 78.5 | ± 27.6 | 74.3 |  |
|  | USNDB | 88.6 | ± 43.6 | 81.2 | 3.5 | 87.0 | ± 28.1 | 83.0 | 8.5 |
| Fatty acids, total saturated (g/day) | ENDB | 34.4 | ± 18.5 | 31.4 |  | 31.7 | ± 11.6 | 30.0 |  |
|  | USNDB | 31.6 | ± 16.8 | 28.9 | -2.7 | 31.8 | ± 10.6 | 30.5 | 0.1 |
| Fatty acids, total monounsaturated (g/day) | ENDB | 25.8 | ± 14.6 | 22.9 |  | 23.9 | ± 9.0 | 22.5 |  |
|  | USNDB | 32.3 | ± 17.7 | 28.9 | 6.6 | 31.3 | ± 11.0 | 29.5 | 7.3 |
| Fatty acids, total polyunsaturated (g/day) | ENDB | 15.9 | ± 10.4 | 13.5 |  | 14.5 | ± 6.1 | 13.5 |  |
|  | USNDB | 17.5 | ± 10.4 | 15.0 | 1.6 | 16.7 | ± 6.3 | 15.6 | 2.2 |
| Cholesterol (mg/day) | ENDB | 243.1 | ± 176.3 | 200.8 |  | 233.1 | ± 89.2 | 220.1 |  |
|  | USNDB | 269.0 | ± 173.9 | 229.1 | 26.0 | 262.4 | ± 93.0 | 250.5 | 29.3 |
| Total proteins (g/day) | ENDB | 86.2 | ± 35.2 | 80.9 |  | 86.7 | ± 23.9 | 84.4 |  |
|  | USNDB | 82.4 | ± 32.0 | 78.0 | -3.8 | 83.9 | ± 22.9 | 81.4 | -2.8 |
| Carbohydrates (g/day) | ENDB | 240.2 | ± 95.6 | 226.9 |  | 229.0 | ± 71.9 | 218.4 |  |
|  | USNDB | 264.1 | ± 102.0 | 249.4 | 23.9 | 256.7 | ± 76.5 | 246.0 | 27.7 |
| Sugar, total (g/day) | ENDB | 123.3 | ± 60.7 | 113.8 |  | 115.7 | ± 43.2 | 109.6 |  |
|  | USNDB | 113.9 | ± 59.8 | 103.1 | -9.4 | 110.3 | ± 44.0 | 103.7 | -5.4 |
| Starch (g/day) | ENDB | 116.1 | ± 52.9 | 106.8 |  | 112.6 | ± 41.3 | 105.6 |  |
|  | USNDB | 46.8 | ± 32.8 | 41.2 | -69.3 | 51.2 | ± 22.7 | 47.3 | -61.4 |
| Dietary fiber, total (g/day) | ENDB | 22.4 | ± 9.8 | 21.1 |  | 23.0 | ± 6.5 | 22.3 |  |
|  | USNDB | 21.4 | ± 9.9 | 19.9 | -1.0 | 23.5 | ± 6.8 | 22.9 | 0.5 |
| Alcohol (g/day) | ENDB | 15.3 | ± 26.7 | 0.0 |  | 11.1 | ± 15.6 | 5.0 |  |
|  | USNDB | 15.5 | ± 26.9 | 0.0 | 0.3 | 11.4 | ± 15.9 | 5.1 | 0.3 |
| Calcium, Ca (mg/day) | ENDB | 1046.5 | ± 513.1 | 975.8 |  | 1088.6 | ± 422.5 | 1035.9 |  |
|  | USNDB | 1141.0 | ± 553.9 | 1073.6 | 94.5 | 1178.6 | ± 438.9 | 1129.0 | 90.0 |
| Iron, Fe (mg/day) | ENDB | 12.1 | ± 5.0 | 11.3 |  | 12.4 | ± 3.2 | 12.0 |  |
|  | USNDB | 11.8 | ± 5.1 | 10.9 | -0.3 | 12.0 | ± 3.4 | 11.5 | -0.4 |
| Potassium, K (mg/day) | ENDB | 3765.1 | ± 1299.5 | 3658.6 |  | 3847.7 | ± 928.0 | 3762.2 |  |
|  | USNDB | 3367.6 | ± 1131.5 | 3261.5 | -397.4 | 3449.1 | ± 826.7 | 3381.9 | -398.6 |
| Magnesium, Mg (mg/day) | ENDB | 349.6 | ± 122.0 | 332.6 |  | 355.8 | ± 91.5 | 344.7 |  |
|  | USNDB | 368.6 | ± 144.7 | 345.7 | 19.0 | 382.4 | ± 102.8 | 370.8 | 26.6 |
| Phosphorus, P (mg/day) | ENDB | 1562.8 | ± 585.5 | 1493.9 |  | 1576.9 | ± 453.2 | 1524.2 |  |
|  | USNDB | 1517.1 | ± 583.4 | 1451.5 | -45.7 | 1532.6 | ± 444.6 | 1482.0 | -44.3 |
| Vitamin D (µg/day) | ENDB | 4.3 | ± 4.8 | 3.1 |  | 3.0 | ± 1.4 | 2.8 |  |
|  | USNDB | 3.1 | ± 3.8 | 2.1 | -1.2 | 2.9 | ± 2.0 | 2.3 | -0.1 |
| Vitamin E (alpha-tocopherol) (mg/day) | ENDB | 12.2 | ± 8.2 | 10.4 |  | 12.1 | ± 4.9 | 11.3 |  |
|  | USNDB | 11.3 | ± 6.7 | 9.9 | -0.9 | 10.5 | ± 3.7 | 9.9 | -1.6 |
| Retinol (µg/day) | ENDB | 837.3 | ± 1483.0 | 483.2 |  | 750.8 | ± 521.1 | 614.9 |  |
|  | USNDB | 827.2 | ± 1212.3 | 534.6 | -10.1 | 776.4 | ± 485.1 | 665.5 | 25.6 |
| Beta-carotene (µg/day) | ENDB | 2000.8 | ± 2643.0 | 1148.3 |  | 2541.5 | ± 1129.1 | 2353.8 |  |
|  | USNDB | 2015.0 | ± 3224.0 | 934.3 | 14.2 | 2707.0 | ± 1295.5 | 2483.0 | 165.6 |
| Thiamin, B1 (mg/day) | ENDB | 1.1 | ± 0.5 | 1.1 |  | 1.1 | ± 0.3 | 1.1 |  |
|  | USNDB | 1.7 | ± 0.7 | 1.6 | 0.6 | 1.7 | ± 0.5 | 1.6 | 0.5 |
| Riboflavin, B2 (mg/day) | ENDB | 1.5 | ± 0.8 | 1.4 |  | 1.6 | ± 0.6 | 1.5 |  |
|  | USNDB | 2.4 | ± 0.9 | 2.3 | 0.9 | 2.4 | ± 0.7 | 2.4 | 0.8 |
| Cobalamin, B12 (µg/day) | ENDB | 4.5 | ± 5.6 | 3.4 |  | 4.6 | ± 2.2 | 4.2 |  |
|  | USNDB | 5.8 | ± 5.1 | 4.6 | 1.3 | 5.7 | ± 2.4 | 5.3 | 1.1 |
| Vitamin B6 (mg/day) | ENDB | 1.7 | ± 0.7 | 1.6 |  | 1.7 | ± 0.5 | 1.6 |  |
|  | USNDB | 2.0 | ± 1.0 | 1.8 | 0.3 | 2.0 | ± 0.6 | 1.9 | 0.3 |
| Vitamin C (mg/day) | ENDB | 104.1 | ± 79.8 | 85.8 |  | 113.2 | ± 45.4 | 106.9 |  |
|  | USNDB | 106.3 | ± 94.8 | 81.4 | 2.2 | 112.4 | ± 52.7 | 103.8 | -0.8 |
| Folate, food (µg/day) | ENDB | 271.9 | ± 150.6 | 248.4 |  | 287.2 | ± 75.0 | 280.2 |  |
|  | USNDB | 289.2 | ± 134.2 | 265.5 | 17.3 | 303.3 | ± 76.2 | 296.2 | 16.2 |
| * Absolute differences in mean nutrient intake between the USNDB and ENDB were statistically significant (paired samples t-test: p < 0.001) for all nutrients | | | | | | | | | |

| **Table 1Sf:** Mean, standard deviation and median of dietary intakes of 28 nutrients of the U.S. nutrient database (USNDB) and the EPIC nutrient database (ENDB) and their absolute mean difference in nutrient intake, reported for the 24-hour dietary recall data (24-HDR) and the dietary questionnaire data (DQ) Germany | | | | | | | | | |
| --- | --- | --- | --- | --- | --- | --- | --- | --- | --- |
| **Food component** | **Database** | **24-HDR (N = 4,418)** | | | | **DQ (N = 52,013)** | | | |
|  |  | **Mean** | **Standard deviation** | **Median** | **Mean difference*** | **Mean** | **Standard deviation** | **Median** | **Mean difference*** |
| Energy (kcal/day) | ENDB | 2174.9 | ± 811.7 | 2073.4 |  | 2050.2 | ± 643.8 | 1954.5 |  |
|  | USNDB | 2267.2 | ± 840.6 | 2164.1 | 92.4 | 2149.0 | ± 646.5 | 2056.7 | 98.8 |
| Water (g/day) | ENDB | 2809.4 | ± 890.3 | 2707.8 |  | 2428.7 | ± 842.2 | 2284.3 |  |
|  | USNDB | 2797.6 | ± 889.3 | 2698.0 | -11.8 | 2415.4 | ± 842.1 | 2270.8 | -13.3 |
| Total fats (g/day) | ENDB | 92.0 | ± 45.3 | 85.2 |  | 80.3 | ± 30.3 | 75.3 |  |
|  | USNDB | 96.3 | ± 46.8 | 89.3 | 4.3 | 85.3 | ± 30.7 | 80.3 | 5.0 |
| Fatty acids, total saturated (g/day) | ENDB | 37.6 | ± 20.3 | 33.9 |  | 33.3 | ± 13.7 | 30.9 |  |
|  | USNDB | 38.3 | ± 21.3 | 34.1 | 0.7 | 33.6 | ± 13.4 | 31.2 | 0.3 |
| Fatty acids, total monounsaturated (g/day) | ENDB | 31.5 | ± 16.8 | 28.7 |  | 27.8 | ± 10.9 | 25.9 |  |
|  | USNDB | 34.0 | ± 17.5 | 31.2 | 2.4 | 30.5 | ± 11.2 | 28.6 | 2.7 |
| Fatty acids, total polyunsaturated (g/day) | ENDB | 16.6 | ± 12.0 | 13.4 |  | 13.6 | ± 5.8 | 12.4 |  |
|  | USNDB | 15.9 | ± 10.8 | 13.3 | -0.7 | 13.5 | ± 5.1 | 12.6 | -0.1 |
| Cholesterol (mg/day) | ENDB | 318.9 | ± 209.4 | 272.5 |  | 309.3 | ± 127.6 | 288.9 |  |
|  | USNDB | 341.5 | ± 284.3 | 250.0 | 22.6 | 348.0 | ± 164.5 | 320.7 | 38.7 |
| Total proteins (g/day) | ENDB | 78.0 | ± 34.6 | 72.3 |  | 76.1 | ± 24.9 | 72.3 |  |
|  | USNDB | 73.8 | ± 30.1 | 69.9 | -4.1 | 74.6 | ± 23.4 | 71.4 | -1.5 |
| Carbohydrates (g/day) | ENDB | 227.1 | ± 92.3 | 214.5 |  | 228.0 | ± 77.6 | 217.0 |  |
|  | USNDB | 252.4 | ± 103.0 | 237.6 | 25.3 | 248.7 | ± 80.6 | 237.5 | 20.7 |
| Sugar, total (g/day) | ENDB | 109.8 | ± 66.9 | 98.3 |  | 107.2 | ± 53.0 | 96.8 |  |
|  | USNDB | 105.4 | ± 63.5 | 94.3 | -4.4 | 100.9 | ± 48.1 | 91.9 | -6.3 |
| Starch (g/day) | ENDB | 106.3 | ± 48.8 | 99.7 |  | 111.9 | ± 39.4 | 107.4 |  |
|  | USNDB | 26.1 | ± 38.0 | 13.9 | -80.2 | 23.8 | ± 12.3 | 21.6 | -88.1 |
| Dietary fiber, total (g/day) | ENDB | 21.8 | ± 9.8 | 20.2 |  | 21.6 | ± 6.8 | 20.8 |  |
|  | USNDB | 21.3 | ± 10.0 | 19.8 | -0.5 | 21.0 | ± 6.7 | 20.3 | -0.6 |
| Alcohol (g/day) | ENDB | 18.1 | ± 25.7 | 7.9 |  | 15.8 | ± 20.1 | 8.9 |  |
|  | USNDB | 18.2 | ± 26.1 | 7.2 | 0.0 | 16.1 | ± 20.4 | 9.4 | 0.3 |
| Calcium, Ca (mg/day) | ENDB | 959.3 | ± 474.6 | 874.6 |  | 897.8 | ± 368.1 | 833.3 |  |
|  | USNDB | 927.3 | ± 476.9 | 828.5 | -32.1 | 942.4 | ± 386.0 | 870.9 | 44.6 |
| Iron, Fe (mg/day) | ENDB | 13.9 | ± 5.7 | 13.0 |  | 13.4 | ± 3.9 | 12.9 |  |
|  | USNDB | 12.2 | ± 5.8 | 11.1 | -1.7 | 12.4 | ± 4.1 | 11.8 | -1.0 |
| Potassium, K (mg/day) | ENDB | 3390.9 | ± 1224.1 | 3241.3 |  | 3121.5 | ± 866.4 | 3010.3 |  |
|  | USNDB | 2952.4 | ± 1069.8 | 2819.5 | -438.5 | 2782.6 | ± 767.2 | 2686.3 | -338.9 |
| Magnesium, Mg (mg/day) | ENDB | 415.7 | ± 139.6 | 398.3 |  | 378.5 | ± 105.1 | 365.6 |  |
|  | USNDB | 318.9 | ± 118.7 | 300.6 | -96.8 | 319.4 | ± 89.6 | 308.0 | -59.1 |
| Phosphorus, P (mg/day) | ENDB | 1320.8 | ± 517.8 | 1245.5 |  | 1350.3 | ± 422.3 | 1289.7 |  |
|  | USNDB | 1277.3 | ± 507.7 | 1205.2 | -43.5 | 1305.9 | ± 411.2 | 1246.9 | -44.5 |
| Vitamin D (µg/day) | ENDB | 3.6 | ± 7.8 | 1.8 |  | 3.9 | ± 3.0 | 3.3 |  |
|  | USNDB | 2.7 | ± 3.4 | 1.8 | -1.0 | 2.8 | ± 1.8 | 2.4 | -1.1 |
| Vitamin E (alpha-tocopherol) (mg/day) | ENDB | 15.4 | ± 12.0 | 12.1 |  | 12.6 | ± 5.1 | 11.5 |  |
|  | USNDB | 9.4 | ± 6.2 | 8.0 | -6.1 | 8.7 | ± 3.3 | 8.2 | -3.9 |
| Retinol (µg/day) | ENDB | 935.6 | ± 2342.7 | 533.5 |  | 841.5 | ± 549.3 | 713.6 |  |
|  | USNDB | 1104.0 | ± 1494.5 | 651.4 | 168.4 | 899.7 | ± 604.1 | 747.2 | 58.2 |
| Beta-carotene (µg/day) | ENDB | 3586.3 | ± 4708.9 | 2246.2 |  | 2694.7 | ± 1336.0 | 2437.8 |  |
|  | USNDB | 2869.8 | ± 5416.8 | 1191.9 | -716.5 | 2096.9 | ± 1285.5 | 1823.8 | -597.8 |
| Thiamin, B1 (mg/day) | ENDB | 1.3 | ± 0.7 | 1.2 |  | 1.2 | ± 0.4 | 1.2 |  |
|  | USNDB | 1.8 | ± 0.8 | 1.6 | 0.5 | 1.7 | ± 0.5 | 1.6 | 0.5 |
| Riboflavin, B2 (mg/day) | ENDB | 1.6 | ± 0.8 | 1.4 |  | 1.5 | ± 0.5 | 1.4 |  |
|  | USNDB | 2.2 | ± 0.9 | 2.1 | 0.7 | 2.1 | ± 0.7 | 2.0 | 0.6 |
| Cobalamin, B12 (µg/day) | ENDB | 6.3 | ± 8.7 | 4.6 |  | 6.2 | ± 2.9 | 5.7 |  |
|  | USNDB | 5.9 | ± 8.0 | 4.3 | -0.4 | 5.8 | ± 2.8 | 5.3 | -0.4 |
| Vitamin B6 (mg/day) | ENDB | 1.7 | ± 0.8 | 1.6 |  | 1.6 | ± 0.5 | 1.6 |  |
|  | USNDB | 1.8 | ± 0.8 | 1.7 | 0.1 | 1.7 | ± 0.5 | 1.6 | 0.1 |
| Vitamin C (mg/day) | ENDB | 123.8 | ± 95.3 | 99.0 |  | 112.1 | ± 54.2 | 101.3 |  |
|  | USNDB | 109.0 | ± 101.6 | 76.9 | -14.8 | 103.9 | ± 60.8 | 90.0 | -8.2 |
| Folate, food (µg/day) | ENDB | 247.6 | ± 146.0 | 225.0 |  | 247.2 | ± 70.9 | 238.9 |  |
|  | USNDB | 290.0 | ± 146.6 | 263.6 | 42.4 | 293.1 | ± 84.7 | 282.7 | 45.9 |
| * Absolute differences in mean nutrient intake between the USNDB and ENDB were statistically significant (paired samples t-test: p < 0.001) for all nutrients | | | | | | | | | |

| **Table 1Sg:** Mean, standard deviation and median of dietary intakes of 28 nutrients of the U.S. nutrient database (USNDB) and the EPIC nutrient database (ENDB) and their absolute mean difference in nutrient intake, reported for the 24-hour dietary recall data (24-HDR) and the dietary questionnaire data (DQ) for Sweden | | | | | | | | | |
| --- | --- | --- | --- | --- | --- | --- | --- | --- | --- |
| **Food component** | **Database** | **24-HDR (N = 6,132)** | | | | **DQ (N = 52,750)** | | | |
|  |  | Mean | Standard deviation | Median | Mean difference* | Mean | Standard deviation | Median | Mean difference* |
| Energy (kcal/day) | ENDB | 2066.5 | ± 750.0 | 1966.2 |  | 2039.4 | ± 642.1 | 1961.8 |  |
|  | USNDB | 2060.3 | ± 762.9 | 1959.7 | -6.3 | 2067.9 | ± 639.0 | 1992.5 | 28.5 |
| Water (g/day) | ENDB | 2485.7 | ± 799.3 | 2377.8 |  | 2197.8 | ± 793.5 | 2086.3 |  |
|  | USNDB | 2460.3 | ± 797.8 | 2350.0 | -25.4 | 2186.7 | ± 798.0 | 2074.4 | -11.1 |
| Total fats (g/day) | ENDB | 86.0 | ± 42.1 | 79.0 |  | 81.4 | ± 32.8 | 76.4 |  |
|  | USNDB | 85.0 | ± 42.1 | 77.7 | -1.0 | 81.7 | ± 32.4 | 76.5 | 0.2 |
| Fatty acids, total saturated (g/day) | ENDB | 37.6 | ± 19.6 | 34.2 |  | 35.1 | ± 15.1 | 32.4 |  |
|  | USNDB | 29.3 | ± 15.3 | 26.4 | -8.3 | 27.9 | ± 12.1 | 25.9 | -7.2 |
| Fatty acids, total monounsaturated (g/day) | ENDB | 30.7 | ± 15.7 | 28.0 |  | 28.4 | ± 11.6 | 26.6 |  |
|  | USNDB | 32.0 | ± 17.1 | 28.7 | 1.4 | 31.1 | ± 12.8 | 28.9 | 2.7 |
| Fatty acids, total polyunsaturated (g/day) | ENDB | 11.5 | ± 6.5 | 10.2 |  | 12.0 | ± 5.5 | 10.9 |  |
|  | USNDB | 17.1 | ± 10.5 | 14.7 | 5.6 | 16.0 | ± 6.7 | 14.8 | 4.1 |
| Cholesterol (mg/day) | ENDB | 338.6 | ± 225.5 | 277.4 |  | 282.2 | ± 149.5 | 250.5 |  |
|  | USNDB | 264.6 | ± 172.1 | 222.5 | -74.1 | 234.4 | ± 120.6 | 211.7 | -47.8 |
| Total proteins (g/day) | ENDB | 79.5 | ± 29.2 | 75.7 |  | 76.6 | ± 24.8 | 74.1 |  |
|  | USNDB | 75.7 | ± 27.9 | 72.3 | -3.9 | 76.5 | ± 25.6 | 73.9 | -0.1 |
| Carbohydrates (g/day) | ENDB | 228.6 | ± 83.6 | 217.4 |  | 236.9 | ± 75.7 | 226.5 |  |
|  | USNDB | 238.1 | ± 95.8 | 222.5 | 9.6 | 249.6 | ± 79.1 | 239.2 | 12.7 |
| Sugar, total (g/day) | ENDB | 100.0 | ± 49.6 | 92.0 |  | 99.3 | ± 40.1 | 93.8 |  |
|  | USNDB | 90.8 | ± 47.5 | 83.1 | -9.2 | 92.9 | ± 38.7 | 87.8 | -6.4 |
| Starch (g/day) | ENDB | 128.4 | ± 49.8 | 121.3 |  | 137.6 | ± 49.2 | 128.7 |  |
|  | USNDB | 36.5 | ± 42.3 | 23.7 | -91.9 | 42.5 | ± 26.5 | 36.1 | -95.1 |
| Dietary fiber, total (g/day) | ENDB | 17.8 | ± 7.8 | 16.7 |  | 19.8 | ± 7.1 | 18.9 |  |
|  | USNDB | 19.3 | ± 9.8 | 17.4 | 1.5 | 21.5 | ± 8.1 | 20.1 | 1.7 |
| Alcohol (g/day) | ENDB | 8.6 | ± 18.1 | 0.0 |  | 7.5 | ± 10.4 | 3.9 |  |
|  | USNDB | 9.6 | ± 19.2 | 0.0 | 1.0 | 8.4 | ± 11.4 | 4.4 | 0.9 |
| Calcium, Ca (mg/day) | ENDB | 937.8 | ± 433.7 | 878.6 |  | 990.5 | ± 404.0 | 939.0 |  |
|  | USNDB | 977.6 | ± 461.7 | 910.8 | 39.8 | 1089.7 | ± 443.2 | 1032.4 | 99.2 |
| Iron, Fe (mg/day) | ENDB | 10.2 | ± 5.3 | 9.3 |  | 11.1 | ± 4.3 | 10.4 |  |
|  | USNDB | 11.4 | ± 5.4 | 10.3 | 1.2 | 11.0 | ± 4.1 | 10.4 | -0.1 |
| Potassium, K (mg/day) | ENDB | 3291.5 | ± 1118.0 | 3175.3 |  | 3360.9 | ± 992.8 | 3236.8 |  |
|  | USNDB | 2949.0 | ± 989.2 | 2853.4 | -342.4 | 3037.7 | ± 893.0 | 2936.8 | -323.2 |
| Magnesium, Mg (mg/day) | ENDB | 313.6 | ± 101.4 | 302.4 |  | 323.4 | ± 92.9 | 312.1 |  |
|  | USNDB | 306.3 | ± 126.5 | 282.6 | -7.4 | 316.6 | ± 97.6 | 304.0 | -6.8 |
| Phosphorus, P (mg/day) | ENDB | 1351.4 | ± 483.3 | 1292.6 |  | 1374.5 | ± 443.8 | 1327.6 |  |
|  | USNDB | 1359.1 | ± 530.4 | 1279.1 | 7.7 | 1403.3 | ± 458.7 | 1350.7 | 28.8 |
| Vitamin D (µg/day) | ENDB | 7.2 | ± 5.6 | 5.7 |  | 6.5 | ± 3.1 | 5.9 |  |
|  | USNDB | 4.6 | ± 4.0 | 3.7 | -2.6 | 5.2 | ± 3.2 | 4.5 | -1.3 |
| Vitamin E (alpha-tocopherol) (mg/day) | ENDB | 8.5 | ± 4.1 | 7.7 |  | 8.6 | ± 3.8 | 7.9 |  |
|  | USNDB | 9.2 | ± 5.3 | 8.1 | 0.7 | 11.7 | ± 7.3 | 9.6 | 3.1 |
| Retinol (µg/day) | ENDB | 1427.7 | ± 1825.0 | 840.8 |  | 1172.3 | ± 1001.4 | 921.7 |  |
|  | USNDB | 916.0 | ± 897.8 | 738.1 | -511.7 | 933.2 | ± 593.6 | 809.3 | -239.1 |
| Beta-carotene (µg/day) | ENDB | 2006.1 | ± 2614.7 | 929.5 |  | 2473.3 | ± 2380.9 | 1779.5 |  |
|  | USNDB | 2387.0 | ± 3486.3 | 1012.3 | 380.9 | 3121.8 | ± 3116.5 | 2217.6 | 648.5 |
| Thiamin, B1 (mg/day) | ENDB | 1.4 | ± 0.6 | 1.2 |  | 1.3 | ± 0.4 | 1.2 |  |
|  | USNDB | 1.7 | ± 1.5 | 1.5 | 0.4 | 1.6 | ± 0.6 | 1.5 | 0.3 |
| Riboflavin, B2 (mg/day) | ENDB | 1.7 | ± 0.8 | 1.6 |  | 1.7 | ± 0.6 | 1.6 |  |
|  | USNDB | 2.4 | ± 0.9 | 2.2 | 0.6 | 2.1 | ± 0.7 | 2.0 | 0.4 |
| Cobalamin, B12 (µg/day) | ENDB | 7.1 | ± 9.1 | 4.8 |  | 6.2 | ± 4.8 | 5.2 |  |
|  | USNDB | 6.8 | ± 6.3 | 5.4 | -0.3 | 6.6 | ± 3.7 | 5.9 | 0.4 |
| Vitamin B6 (mg/day) | ENDB | 1.8 | ± 0.7 | 1.7 |  | 1.9 | ± 0.6 | 1.8 |  |
|  | USNDB | 1.9 | ± 0.9 | 1.8 | 0.1 | 1.9 | ± 0.6 | 1.9 | 0.1 |
| Vitamin C (mg/day) | ENDB | 91.5 | ± 71.2 | 73.1 |  | 104.2 | ± 59.6 | 92.4 |  |
|  | USNDB | 81.9 | ± 71.1 | 59.7 | -9.7 | 93.1 | ± 54.3 | 82.0 | -11.1 |
| Folate, food (µg/day) | ENDB | 223.0 | ± 94.2 | 211.0 |  | 244.4 | ± 80.5 | 234.2 |  |
|  | USNDB | 222.5 | ± 90.0 | 209.0 | -0.5 | 216.8 | ± 72.4 | 207.9 | -27.6 |
| * Absolute differences in mean nutrient intake between the USNDB and ENDB were statistically significant (paired samples t-test: p < 0.001) for all nutrients | | | | | | | | | |

| **Table 1Sh:** Mean, standard deviation and median of dietary intakes of 28 nutrients of the U.S. nutrient database (USNDB) and the EPIC nutrient database (ENDB) and their absolute mean difference in nutrient intake, reported for the 24-hour dietary recall data (24-HDR) and the dietary questionnaire data (DQ) for Denmark | | | | | | | | | |
| --- | --- | --- | --- | --- | --- | --- | --- | --- | --- |
| **Food component** | **Database** | **24-HDR (N = 3,918)** | | | | **DQ (N = 55,860)** | | | |
|  |  | **Mean** | **Standard deviation** | **Median** | **Mean difference*** | **Mean** | **Standard deviation** | **Median** | **Mean difference*** |
| Energy (kcal/day) | ENDB | 2245.5 | ± 817.6 | 2149.8 |  | 2202.6 | ± 596.2 | 2137.3 |  |
|  | USNDB | 2259.3 | ± 803.9 | 2157.7 | 13.7 | 2242.5 | ± 605.9 | 2180.2 | 40.0 |
| Water (g/day) | ENDB | 3155.4 | ± 974.8 | 3074.5 |  | 3206.0 | ± 829.9 | 3132.3 |  |
|  | USNDB | 3131.3 | ± 969.0 | 3046.7 | -24.0 | 3198.7 | ± 824.2 | 3126.6 | -7.2 |
| Total fats (g/day) | ENDB | 90.7 | ± 47.1 | 82.3 |  | 82.7 | ± 27.8 | 79.1 |  |
|  | USNDB | 86.3 | ± 43.3 | 78.9 | -4.4 | 79.3 | ± 26.7 | 76.0 | -3.4 |
| Fatty acids, total saturated (g/day) | ENDB | 37.7 | ± 21.6 | 33.5 |  | 33.3 | ± 12.0 | 31.8 |  |
|  | USNDB | 29.5 | ± 16.9 | 26.4 | -8.2 | 26.7 | ± 9.9 | 25.4 | -6.6 |
| Fatty acids, total monounsaturated (g/day) | ENDB | 31.3 | ± 17.5 | 28.3 |  | 28.6 | ± 10.3 | 27.1 |  |
|  | USNDB | 32.0 | ± 16.5 | 29.3 | 0.7 | 29.1 | ± 10.0 | 27.8 | 0.5 |
| Fatty acids, total polyunsaturated (g/day) | ENDB | 13.0 | ± 8.0 | 11.1 |  | 12.6 | ± 4.8 | 11.8 |  |
|  | USNDB | 16.9 | ± 10.9 | 14.4 | 3.9 | 15.7 | ± 6.3 | 14.7 | 3.1 |
| Cholesterol (mg/day) | ENDB | 381.8 | ± 247.0 | 323.0 |  | 413.4 | ± 168.2 | 385.4 |  |
|  | USNDB | 290.4 | ± 191.8 | 246.6 | -91.4 | 323.0 | ± 129.1 | 302.5 | -90.4 |
| Total proteins (g/day) | ENDB | 83.0 | ± 31.2 | 78.3 |  | 94.6 | ± 26.9 | 91.5 |  |
|  | USNDB | 74.4 | ± 27.6 | 70.5 | -8.6 | 85.2 | ± 24.3 | 82.5 | -9.4 |
| Carbohydrates (g/day) | ENDB | 231.6 | ± 87.9 | 220.7 |  | 234.2 | ± 70.3 | 226.0 |  |
|  | USNDB | 255.9 | ± 101.3 | 242.2 | 24.3 | 267.1 | ± 83.3 | 256.1 | 32.9 |
| Sugar, total (g/day) | ENDB | 102.3 | ± 55.6 | 92.5 |  | 103.4 | ± 45.5 | 95.5 |  |
|  | USNDB | 103.5 | ± 67.7 | 89.9 | 1.3 | 112.2 | ± 57.9 | 100.4 | 8.7 |
| Starch (g/day) | ENDB | 115.7 | ± 49.9 | 107.8 |  | 117.4 | ± 38.3 | 113.5 |  |
|  | USNDB | 21.6 | ± 26.2 | 12.4 | -94.1 | 23.1 | ± 14.1 | 19.9 | -94.3 |
| Dietary fiber, total (g/day) | ENDB | 24.6 | ± 10.6 | 23.1 |  | 25.0 | ± 8.2 | 24.2 |  |
|  | USNDB | 21.2 | ± 9.5 | 19.7 | -3.4 | 22.9 | ± 7.6 | 22.0 | -2.1 |
| Alcohol (g/day) | ENDB | 24.4 | ± 30.5 | 13.7 |  | 20.4 | ± 21.5 | 12.9 |  |
|  | USNDB | 27.0 | ± 34.0 | 14.2 | 2.5 | 22.5 | ± 23.3 | 14.4 | 2.0 |
| Calcium, Ca (mg/day) | ENDB | 1009.5 | ± 504.1 | 925.9 |  | 1081.7 | ± 447.6 | 1010.5 |  |
|  | USNDB | 886.2 | ± 458.1 | 802.3 | -123.3 | 971.2 | ± 419.5 | 895.8 | -110.5 |
| Iron, Fe (mg/day) | ENDB | 12.4 | ± 5.0 | 11.7 |  | 13.2 | ± 3.6 | 12.8 |  |
|  | USNDB | 12.7 | ± 5.7 | 11.6 | 0.2 | 12.6 | ± 3.8 | 12.1 | -0.6 |
| Potassium, K (mg/day) | ENDB | 3617.7 | ± 1230.3 | 3477.2 |  | 4121.2 | ± 1038.1 | 4024.8 |  |
|  | USNDB | 3109.5 | ± 1059.3 | 2999.4 | -508.2 | 3515.9 | ± 905.5 | 3426.6 | -605.3 |
| Magnesium, Mg (mg/day) | ENDB | 371.3 | ± 121.1 | 355.7 |  | 392.2 | ± 95.9 | 384.4 |  |
|  | USNDB | 323.2 | ± 110.6 | 308.6 | -48.1 | 348.0 | ± 90.1 | 339.1 | -44.2 |
| Phosphorus, P (mg/day) | ENDB | 1581.7 | ± 580.8 | 1503.0 |  | 1758.2 | ± 515.6 | 1701.6 |  |
|  | USNDB | 1303.3 | ± 494.4 | 1239.1 | -278.3 | 1452.4 | ± 444.6 | 1399.1 | -305.8 |
| Vitamin D (µg/day) | ENDB | 4.7 | ± 8.9 | 2.2 |  | 4.6 | ± 2.2 | 4.2 |  |
|  | USNDB | 3.5 | ± 5.1 | 2.0 | -1.2 | 3.9 | ± 2.2 | 3.4 | -0.6 |
| Vitamin E (alpha-tocopherol) (mg/day) | ENDB | 9.5 | ± 5.8 | 8.3 |  | 9.4 | ± 3.4 | 8.9 |  |
|  | USNDB | 6.9 | ± 3.9 | 6.0 | -2.6 | 7.1 | ± 2.7 | 6.7 | -2.3 |
| Retinol (µg/day) | ENDB | 1028.0 | ± 1854.2 | 538.1 |  | 1150.0 | ± 785.0 | 976.5 |  |
|  | USNDB | 767.9 | ± 1285.7 | 553.3 | -260.0 | 779.4 | ± 400.5 | 709.2 | -370.6 |
| Beta-carotene (µg/day) | ENDB | 3325.9 | ± 5537.3 | 1176.7 |  | 4217.6 | ± 4701.6 | 2641.6 |  |
|  | USNDB | 2966.7 | ± 4923.7 | 1011.8 | -359.2 | 3599.4 | ± 4122.4 | 2230.9 | -618.2 |
| Thiamin, B1 (mg/day) | ENDB | 1.2 | ± 0.5 | 1.1 |  | 1.3 | ± 0.4 | 1.3 |  |
|  | USNDB | 1.8 | ± 0.7 | 1.7 | 0.6 | 2.0 | ± 0.5 | 1.9 | 0.7 |
| Riboflavin, B2 (mg/day) | ENDB | 1.8 | ± 0.8 | 1.7 |  | 2.1 | ± 0.7 | 2.0 |  |
|  | USNDB | 2.7 | ± 1.0 | 2.6 | 0.9 | 2.9 | ± 0.9 | 2.8 | 0.8 |
| Cobalamin, B12 (µg/day) | ENDB | 6.9 | ± 8.1 | 5.1 |  | 8.3 | ± 3.7 | 7.6 |  |
|  | USNDB | 6.2 | ± 7.6 | 4.5 | -0.7 | 7.5 | ± 3.3 | 6.9 | -0.8 |
| Vitamin B6 (mg/day) | ENDB | 1.7 | ± 0.7 | 1.6 |  | 2.0 | ± 0.6 | 1.9 |  |
|  | USNDB | 1.8 | ± 0.8 | 1.7 | 0.1 | 2.1 | ± 0.6 | 2.1 | 0.2 |
| Vitamin C (mg/day) | ENDB | 103.7 | ± 95.5 | 76.0 |  | 105.0 | ± 52.8 | 94.5 |  |
|  | USNDB | 84.3 | ± 84.2 | 58.0 | -19.4 | 86.2 | ± 47.5 | 75.7 | -18.8 |
| Folate, food (µg/day) | ENDB | 284.9 | ± 113.0 | 267.6 |  | 307.5 | ± 87.3 | 298.9 |  |
|  | USNDB | 284.6 | ± 108.8 | 269.2 | -0.3 | 299.9 | ± 80.9 | 292.8 | -7.7 |
| * Absolute differences in mean nutrient intake between the USNDB and ENDB were statistically significant (paired samples t-test: p < 0.001) for all nutrients | | | | | | | | | |

| **Table 1Si:** Mean, standard deviation and median of dietary intakes of 28 nutrients of the U.S. nutrient database (USNDB) and the EPIC nutrient database (ENDB) and their absolute mean difference in nutrient intake, reported for the 24-hour dietary recall data (24-HDR) and the dietary questionnaire data (DQ) for Norway | | | | | | | | | |
| --- | --- | --- | --- | --- | --- | --- | --- | --- | --- |
| **Food component** | **Database** | **24-HDR (N = 1,798)** | | | | **DQ (N = 36,448)** | | | |
|  |  | **Mean** | **Standard deviation** | **Median** | **Mean difference*** | **Mean** | **Standard deviation** | **Median** | **Mean difference*** |
| Energy (kcal/day) | ENDB | 1864.2 | ± 624.0 | 1797.9 |  | 1648.2 | ± 405.5 | 1623.2 |  |
|  | USNDB | 1903.2 | ± 649.2 | 1814.2 | 38.9 | 1673.0 | ± 408.9 | 1651.4 | 24.9 |
| Water (g/day) | ENDB | 2735.1 | ± 885.8 | 2594.8 |  | 1352.0 | ± 389.1 | 1327.6 |  |
|  | USNDB | 2722.7 | ± 882.4 | 2580.6 | -12.4 | 1358.3 | ± 389.5 | 1333.7 | 6.3 |
| Total fats (g/day) | ENDB | 73.3 | ± 34.2 | 67.9 |  | 61.2 | ± 19.3 | 59.0 |  |
|  | USNDB | 72.8 | ± 35.0 | 67.0 | -0.5 | 60.1 | ± 18.9 | 58.1 | -1.1 |
| Fatty acids, total saturated (g/day) | ENDB | 30.7 | ± 15.5 | 28.0 |  | 23.9 | ± 8.3 | 22.9 |  |
|  | USNDB | 27.3 | ± 14.3 | 24.6 | -3.4 | 21.0 | ± 7.4 | 20.1 | -2.9 |
| Fatty acids, total monounsaturated (g/day) | ENDB | 23.0 | ± 12.7 | 20.8 |  | 20.0 | ± 6.3 | 19.3 |  |
|  | USNDB | 26.1 | ± 14.4 | 23.8 | 3.1 | 22.2 | ± 7.3 | 21.3 | 2.2 |
| Fatty acids, total polyunsaturated (g/day) | ENDB | 12.5 | ± 8.0 | 10.7 |  | 11.0 | ± 4.2 | 10.2 |  |
|  | USNDB | 13.5 | ± 8.3 | 11.7 | 0.9 | 11.7 | ± 4.0 | 11.2 | 0.7 |
| Cholesterol (mg/day) | ENDB | 278.8 | ± 170.8 | 237.2 |  | 268.8 | ± 83.5 | 261.6 |  |
|  | USNDB | 269.9 | ± 170.5 | 225.9 | -8.9 | 244.0 | ± 76.9 | 237.3 | -24.8 |
| Total proteins (g/day) | ENDB | 77.4 | ± 27.8 | 72.8 |  | 77.1 | ± 20.3 | 75.6 |  |
|  | USNDB | 75.8 | ± 27.6 | 71.8 | -1.5 | 75.4 | ± 19.6 | 74.1 | -1.7 |
| Carbohydrates (g/day) | ENDB | 210.6 | ± 75.3 | 203.3 |  | 192.0 | ± 51.2 | 189.4 |  |
|  | USNDB | 227.5 | ± 84.0 | 218.7 | 16.9 | 206.3 | ± 55.2 | 203.6 | 14.3 |
| Sugar, total (g/day) | ENDB | 97.9 | ± 48.8 | 90.9 |  | 76.4 | ± 27.9 | 73.6 |  |
|  | USNDB | 94.0 | ± 50.2 | 85.3 | -3.9 | 70.0 | ± 26.2 | 67.5 | -6.4 |
| Starch (g/day) | ENDB | 102.1 | ± 43.0 | 97.0 |  | 107.8 | ± 30.7 | 107.4 |  |
|  | USNDB | 44.8 | ± 31.3 | 39.0 | -57.3 | 44.4 | ± 16.1 | 42.4 | -63.4 |
| Dietary fiber, total (g/day) | ENDB | 19.2 | ± 8.3 | 18.0 |  | 20.6 | ± 6.0 | 20.3 |  |
|  | USNDB | 19.0 | ± 8.3 | 18.0 | -0.2 | 19.9 | ± 5.8 | 19.6 | -0.7 |
| Alcohol (g/day) | ENDB | 7.6 | ± 18.0 | 0.0 |  | 3.0 | ± 3.5 | 1.6 |  |
|  | USNDB | 8.7 | ± 20.5 | 0.0 | 1.1 | 3.5 | ± 4.1 | 1.9 | 0.5 |
| Calcium, Ca (mg/day) | ENDB | 808.2 | ± 401.7 | 747.7 |  | 676.6 | ± 257.6 | 643.6 |  |
|  | USNDB | 986.7 | ± 476.8 | 905.8 | 178.5 | 877.6 | ± 310.3 | 841.4 | 201.0 |
| Iron, Fe (mg/day) | ENDB | 9.4 | ± 4.3 | 8.7 |  | 9.4 | ± 2.5 | 9.2 |  |
|  | USNDB | 10.9 | ± 5.5 | 9.9 | 1.5 | 10.4 | ± 3.7 | 9.8 | 1.0 |
| Potassium, K (mg/day) | ENDB | 3377.5 | ± 1161.8 | 3236.6 |  | 3237.6 | ± 827.3 | 3179.1 |  |
|  | USNDB | 3070.5 | ± 1132.6 | 2938.2 | -307.1 | 2828.9 | ± 722.1 | 2777.3 | -408.7 |
| Magnesium, Mg (mg/day) | ENDB | 339.0 | ± 114.7 | 323.5 |  | 312.9 | ± 74.0 | 309.1 |  |
|  | USNDB | 331.3 | ± 125.9 | 311.9 | -7.7 | 307.1 | ± 76.5 | 303.9 | -5.8 |
| Phosphorus, P (mg/day) | ENDB | 1371.0 | ± 478.2 | 1319.5 |  | 1345.3 | ± 349.5 | 1319.3 |  |
|  | USNDB | 1345.8 | ± 499.5 | 1285.1 | -25.2 | 1287.4 | ± 342.3 | 1260.5 | -57.9 |
| Vitamin D (µg/day) | ENDB | 4.2 | ± 5.8 | 2.6 |  | 9.0 | ± 7.7 | 6.1 |  |
|  | USNDB | 2.8 | ± 4.0 | 1.6 | -1.4 | 7.4 | ± 8.3 | 3.5 | -1.7 |
| Vitamin E (alpha-tocopherol) (mg/day) | ENDB | 7.7 | ± 4.2 | 6.9 |  | 9.2 | ± 4.4 | 7.9 |  |
|  | USNDB | 8.6 | ± 4.7 | 7.7 | 0.8 | 8.7 | ± 2.8 | 8.5 | -0.4 |
| Retinol (µg/day) | ENDB | 760.5 | ± 888.6 | 530.2 |  | 919.5 | ± 728.3 | 647.6 |  |
|  | USNDB | 758.2 | ± 786.9 | 499.7 | -2.3 | 1068.8 | ± 988.1 | 664.2 | 149.3 |
| Beta-carotene (µg/day) | ENDB | 2443.9 | ± 3812.2 | 956.4 |  | 3144.8 | ± 1943.9 | 2749.6 |  |
|  | USNDB | 3291.5 | ± 5127.7 | 1371.0 | 847.5 | 4283.8 | ± 2581.0 | 3788.0 | 1139.0 |
| Thiamin, B1 (mg/day) | ENDB | 1.1 | ± 0.5 | 1.1 |  | 1.1 | ± 0.3 | 1.1 |  |
|  | USNDB | 1.6 | ± 1.6 | 1.4 | 0.4 | 1.5 | ± 0.4 | 1.4 | 0.4 |
| Riboflavin, B2 (mg/day) | ENDB | 1.4 | ± 0.6 | 1.3 |  | 1.2 | ± 0.4 | 1.2 |  |
|  | USNDB | 2.2 | ± 0.9 | 2.1 | 0.8 | 1.8 | ± 0.5 | 1.8 | 0.6 |
| Cobalamin, B12 (µg/day) | ENDB | 5.7 | ± 5.5 | 4.2 |  | 5.8 | ± 2.6 | 5.3 |  |
|  | USNDB | 7.4 | ± 6.2 | 5.6 | 1.8 | 7.2 | ± 3.0 | 6.8 | 1.5 |
| Vitamin B6 (mg/day) | ENDB | 1.2 | ± 0.5 | 1.1 |  | 1.3 | ± 0.4 | 1.3 |  |
|  | USNDB | 1.7 | ± 0.7 | 1.7 | 0.5 | 1.9 | ± 0.5 | 1.9 | 0.6 |
| Vitamin C (mg/day) | ENDB | 98.2 | ± 75.3 | 80.1 |  | 84.1 | ± 42.1 | 76.3 |  |
|  | USNDB | 90.2 | ± 75.0 | 69.9 | -7.9 | 85.9 | ± 45.8 | 77.0 | 1.8 |
| Folate, food (µg/day) | ENDB | 223.6 | ± 92.4 | 209.5 |  | 216.6 | ± 61.1 | 211.4 |  |
|  | USNDB | 236.6 | ± 102.3 | 219.3 | 13.0 | 218.6 | ± 63.5 | 212.6 | 2.0 |
| * Absolute differences in mean nutrient intake between the USNDB and ENDB were statistically significant (paired samples t-test: p < 0.001) for all nutrients | | | | | | | | | |
