# Supplementary material for: Comparing Calculated Nutrient Intakes Using Different Food Composition Databases: Results from the European Prospective Investigation into Cancer and Nutrition (EPIC) Cohort
Source: Nutrients. 2020 Sep 23;12(10):2906. doi: 10.3390/nu12102906 (PMC7650652; doi:10.3390/nu12102906)
Supplement: Supplementary file 1 [file nutrients-12-02906-s001.zip › Revision2_Nutrients_TableS2.docx]

**Comparing calculated nutrient intakes using different food composition databases: Results from the European prospective investigation into cancer and nutrition (EPIC) cohort**

Heleen Van Puyvelde, Aurora Perez-Cornago, Corinne Casagrande, Genevieve Nicolas, Vickà Versele, Guri Skeie, Matthias B. Schulze, Ingegerd Johansson, José María Huerta, Oliverio Andreina, Fulvio Ricceri, Jytte Halkjær, Pilar Amiano Etxezarreta, Koen Van Herck, Elisabete Weiderpass, Marc J. Gunter, Inge Huybrechts, on behalf of the EPIC Consortium

**Table 2S**: Pearson correlation coefficients and weighted kappas (κ) for dietary intakes of 28 nutrients of the U.S. nutrient database (USNDB) and the EPIC nutrient database (ENDB), reported for the 24-hour dietary recall data (24-HDR) and the dietary questionnaire data (DQ) by country.

| **Table 2Sa:** Pearson correlation coefficients and weighted kappas (κ) for dietary intakes of 28 nutrients of the U.S. nutrient database (USNDB) and the EPIC nutrient database (ENDB), reported for the 24-hour dietary recall data (24-HDR) and the dietary questionnaire data (DQ) for France. | | | | |
| --- | --- | --- | --- | --- |
|  | **24-HDR (N = 4,735)** | | **DQ (N = 73,035)** | |
|  | **Pearson correlation coefficient*** | **Weighted κ** | **Pearson correlation coefficient*** | **Weighted κ** |
| Energy (kcal/day) | 0.94 | 0.77 | 0.97 | 0.86 |
| Water (g) | 1.00 | 0.95 | 1.00 | 0.97 |
| Total fats (g) | 0.90 | 0.74 | 0.94 | 0.79 |
| Fatty acids, total saturated (g) | 0.87 | 0.71 | 0.92 | 0.77 |
| Fatty acids, total monounsaturated (g) | 0.88 | 0.68 | 0.89 | 0.70 |
| Fatty acids, total polyunsaturated (g) | 0.82 | 0.59 | 0.77 | 0.56 |
| Cholesterol (mg) | 0.91 | 0.73 | 0.97 | 0.86 |
| Total proteins (g) | 0.90 | 0.71 | 0.96 | 0.83 |
| Carbohydrates (g) | 0.92 | 0.76 | 0.98 | 0.86 |
| Sugar, total (g) | 0.95 | 0.80 | 0.97 | 0.84 |
| Starch (g) | 0.73 | 0.48 | 0.94 | 0.76 |
| Dietary fiber, total (g) | 0.81 | 0.65 | 0.93 | 0.81 |
| Alcohol (g) | 0.97 | 0.94 | 0.99 | 0.94 |
| Calcium, Ca (mg) | 0.75 | 0.63 | 0.88 | 0.68 |
| Iron, Fe (mg) | 0.75 | 0.59 | 0.86 | 0.68 |
| Potassium, K (mg) | 0.92 | 0.76 | 0.97 | 0.85 |
| Magnesium, Mg (mg) | 0.61 | 0.39 | 0.57 | 0.40 |
| Phosphorus, P (mg) | 0.84 | 0.66 | 0.94 | 0.78 |
| Vitamin D (µg) | 0.27 | 0.40 | 0.88 | 0.69 |
| Vitamin E (alpha-tocopherol) (mg) | 0.72 | 0.54 | 0.74 | 0.53 |
| Retinol (µg) | 0.85 | 0.66 | 0.99 | 0.88 |
| Beta-carotene (µg) | 0.89 | 0.70 | 0.88 | 0.67 |
| Thiamin, B1 (mg) | 0.62 | 0.51 | 0.73 | 0.54 |
| Riboflavin, B2 (mg) | 0.89 | 0.70 | 0.96 | 0.81 |
| Cobalamin, B12 (µg) | 0.91 | 0.62 | 0.97 | 0.80 |
| Vitamin B6 (mg) | 0.81 | 0.67 | 0.96 | 0.84 |
| Vitamin C (mg) | 0.95 | 0.82 | 0.98 | 0.86 |
| Folate, food (µg) | 0.72 | 0.60 | 0.94 | 0.79 |
| *Pearson correlation coefficients for the 28 nutrient intakes measured by the USNDB and the ENDB were significant at the level of p < 0.001 for all nutrients | | | | |

| **Table 2Sb:** Pearson correlation coefficients and weighted kappas (κ) for dietary intakes of 28 nutrients of the U.S. nutrient database (USNDB) and the EPIC nutrient database (ENDB), reported for the 24-hour dietary recall data (24-HDR) and the dietary questionnaire data (DQ) for Italy | | | | |
| --- | --- | --- | --- | --- |
|  | **24-HDR (N = 3,961)** | | **DQ (N = 45,908)** | |
|  | **Pearson correlation coefficient*** | **Weighted κ** | **Pearson correlation coefficient*** | **Weighted κ** |
| Energy (kcal/day) | 0.98 | 0.87 | 0.99 | 0.92 |
| Water (g) | 1.00 | 0.97 | 1.00 | 0.98 |
| Total fats (g) | 0.96 | 0.83 | 0.99 | 0.89 |
| Fatty acids, total saturated (g) | 0.93 | 0.79 | 0.98 | 0.89 |
| Fatty acids, total monounsaturated (g) | 0.97 | 0.83 | 0.98 | 0.89 |
| Fatty acids, total polyunsaturated (g) | 0.87 | 0.69 | 0.93 | 0.76 |
| Cholesterol (mg) | 0.95 | 0.80 | 0.99 | 0.90 |
| Total proteins (g) | 0.95 | 0.80 | 0.98 | 0.88 |
| Carbohydrates (g) | 0.96 | 0.82 | 0.98 | 0.88 |
| Sugar, total (g) | 0.93 | 0.78 | 0.96 | 0.84 |
| Starch (g) | 0.61 | 0.38 | 0.82 | 0.65 |
| Dietary fiber, total (g) | 0.87 | 0.72 | 0.97 | 0.85 |
| Alcohol (g) | 1.00 | 0.96 | 1.00 | 0.97 |
| Calcium, Ca (mg) | 0.91 | 0.74 | 0.96 | 0.82 |
| Iron, Fe (mg) | 0.66 | 0.57 | 0.87 | 0.68 |
| Potassium, K (mg) | 0.93 | 0.78 | 0.98 | 0.88 |
| Magnesium, Mg (mg) | 0.86 | 0.66 | 0.86 | 0.76 |
| Phosphorus, P (mg) | 0.94 | 0.77 | 0.98 | 0.87 |
| Vitamin D (µg) | 0.45 | 0.41 | 0.73 | 0.55 |
| Vitamin E (alpha-tocopherol) (mg) | 0.91 | 0.72 | 0.94 | 0.80 |
| Retinol (µg) | 0.72 | 0.74 | 0.96 | 0.78 |
| Beta-carotene (µg) | 0.89 | 0.65 | 0.98 | 0.85 |
| Thiamin, B1 (mg) | 0.66 | 0.45 | 0.67 | 0.47 |
| Riboflavin, B2 (mg) | 0.85 | 0.61 | 0.91 | 0.72 |
| Cobalamin, B12 (µg) | 0.94 | 0.68 | 0.97 | 0.83 |
| Vitamin B6 (mg) | 0.84 | 0.71 | 0.95 | 0.80 |
| Vitamin C (mg) | 0.94 | 0.78 | 0.99 | 0.91 |
| Folate, food (µg) | 0.82 | 0.67 | 0.95 | 0.81 |
| *Pearson correlation coefficients for the 28 nutrient intakes measured by the USNDB and the ENDB were significant at the level of p < 0.001 for all nutrients | | | | |

| **Table 2Sc:** Pearson correlation coefficients and weighted kappas (κ) for dietary intakes of 28 nutrients of the U.S. nutrient database (USNDB) and the EPIC nutrient database (ENDB), reported for the 24-hour dietary recall data (24-HDR) and the dietary questionnaire data (DQ) for Spain | | | | |  |
| --- | --- | --- | --- | --- | --- |
|  | **24-HDR (N = 3,220)** | | **DQ (N = 40,621)** | |  |
|  | **Pearson correlation coefficient*** | **Weighted κ** | **Pearson correlation coefficient*** | **Weighted κ** | |
| Energy (kcal/day) | 0.97 | 0.86 | 0.99 | 0.91 |  |
| Water (g) | 1.00 | 0.97 | 1.00 | 0.98 |  |
| Total fats (g) | 0.97 | 0.87 | 0.99 | 0.91 |  |
| Fatty acids, total saturated (g) | 0.92 | 0.80 | 0.95 | 0.83 |  |
| Fatty acids, total monounsaturated (g) | 0.97 | 0.86 | 0.99 | 0.90 |  |
| Fatty acids, total polyunsaturated (g) | 0.94 | 0.77 | 0.92 | 0.75 |  |
| Cholesterol (mg) | 0.94 | 0.80 | 0.93 | 0.79 |  |
| Total proteins (g) | 0.96 | 0.81 | 0.99 | 0.89 |  |
| Carbohydrates (g) | 0.93 | 0.79 | 0.97 | 0.85 |  |
| Sugar, total (g) | 0.93 | 0.80 | 0.97 | 0.84 |  |
| Starch (g) | 0.81 | 0.58 | 0.90 | 0.71 |  |
| Dietary fiber, total (g) | 0.89 | 0.75 | 0.96 | 0.85 |  |
| Alcohol (g) | 0.98 | 0.94 | 0.98 | 0.95 |  |
| Calcium, Ca (mg) | 0.89 | 0.75 | 0.96 | 0.81 |  |
| Iron, Fe (mg) | 0.77 | 0.61 | 0.85 | 0.68 |  |
| Potassium, K (mg) | 0.88 | 0.73 | 0.97 | 0.84 |  |
| Magnesium, Mg (mg) | 0.86 | 0.67 | 0.94 | 0.79 |  |
| Phosphorus, P (mg) | 0.93 | 0.78 | 0.96 | 0.81 |  |
| Vitamin D (µg) | 0.34 | 0.35 | 0.69 | 0.50 |  |
| Vitamin E (alpha-tocopherol) (mg) | 0.88 | 0.66 | 0.85 | 0.71 |  |
| Retinol (µg) | 0.86 | 0.70 | 0.74 | 0.78 |  |
| Beta-carotene (µg) | 0.72 | 0.53 | 0.79 | 0.58 |  |
| Thiamin, B1 (mg) | 0.91 | 0.73 | 0.94 | 0.79 |  |
| Riboflavin, B2 (mg) | 0.89 | 0.71 | 0.91 | 0.72 |  |
| Cobalamin, B12 (µg) | 0.90 | 0.67 | 0.86 | 0.74 |  |
| Vitamin B6 (mg) | 0.85 | 0.65 | 0.80 | 0.76 |  |
| Vitamin C (mg) | 0.95 | 0.82 | 0.99 | 0.92 |  |
| Folate, food (µg) | 0.72 | 0.60 | 0.92 | 0.67 |  |
| *Pearson correlation coefficients for the 28 nutrient intakes measured by the USNDB and the ENDB were significant at the level of p < 0.001 for all nutrients | | | | |  |

| **Table 2Sd:** Pearson correlation coefficients and weighted kappas (κ) for dietary intakes of 28 nutrients of the U.S. nutrient database (USNDB) and the EPIC nutrient database (ENDB), reported for the 24-hour dietary recall data (24-HDR) and the dietary questionnaire data (DQ) for the United Kingdom | | | | |  |
| --- | --- | --- | --- | --- | --- |
|  | **24-HDR (N = 1,315)** | | **DQ (N = 81,097)** | |  |
|  | **Pearson correlation coefficient*** | **Weighted κ** | **Pearson correlation coefficient*** | **Weighted κ** | |
| Energy (kcal/day) | 0.96 | 0.85 | 0.98 | 0.89 |  |
| Water (g) | 1.00 | 0.96 | 1.00 | 0.98 |  |
| Total fats (g) | 0.95 | 0.82 | 0.98 | 0.89 |  |
| Fatty acids, total saturated (g) | 0.94 | 0.76 | 0.98 | 0.87 |  |
| Fatty acids, total monounsaturated (g) | 0.92 | 0.77 | 0.95 | 0.81 |  |
| Fatty acids, total polyunsaturated (g) | 0.82 | 0.68 | 0.94 | 0.77 |  |
| Cholesterol (mg) | 0.88 | 0.72 | 0.94 | 0.81 |  |
| Total proteins (g) | 0.92 | 0.76 | 0.97 | 0.84 |  |
| Carbohydrates (g) | 0.93 | 0.79 | 0.96 | 0.85 |  |
| Sugar, total (g) | 0.89 | 0.76 | 0.92 | 0.82 |  |
| Starch (g) | 0.68 | 0.43 | 0.82 | 0.63 |  |
| Dietary fiber, total (g) | 0.95 | 0.81 | 0.98 | 0.88 |  |
| Alcohol (g) | 0.90 | 0.96 | 1.00 | 0.98 |  |
| Calcium, Ca (mg) | 0.91 | 0.74 | 0.98 | 0.88 |  |
| Iron, Fe (mg) | 0.71 | 0.59 | 0.87 | 0.68 |  |
| Potassium, K (mg) | 0.94 | 0.78 | 0.97 | 0.83 |  |
| Magnesium, Mg (mg) | 0.91 | 0.73 | 0.95 | 0.78 |  |
| Phosphorus, P (mg) | 0.91 | 0.75 | 0.96 | 0.83 |  |
| Vitamin D (µg) | 0.54 | 0.29 | 0.66 | 0.46 |  |
| Vitamin E (alpha-tocopherol) (mg) | 0.64 | 0.50 | 0.61 | 0.45 |  |
| Retinol (µg) | 0.86 | 0.61 | 0.87 | 0.68 |  |
| Beta-carotene (µg) | 0.96 | 0.71 | 0.98 | 0.84 |  |
| Thiamin, B1 (mg) | 0.73 | 0.56 | 0.73 | 0.62 |  |
| Riboflavin, B2 (mg) | 0.83 | 0.62 | 0.90 | 0.70 |  |
| Cobalamin, B12 (µg) | 0.90 | 0.56 | 0.86 | 0.69 |  |
| Vitamin B6 (mg) | 0.63 | 0.59 | 0.78 | 0.73 |  |
| Vitamin C (mg) | 0.92 | 0.77 | 0.97 | 0.84 |  |
| Folate, food (µg) | 0.80 | 0.63 | 0.89 | 0.73 |  |
| *Pearson correlation coefficients for the 28 nutrient intakes measured by the USNDB and the ENDB were significant at the level of p < 0.001 for all nutrients | | | | |  |

| **Table 2Se:** Pearson correlation coefficients and weighted kappas (κ) for dietary intakes of 28 nutrients of the U.S. nutrient database (USNDB) and the EPIC nutrient database (ENDB), reported for the 24-hour dietary recall data (24-HDR) and the dietary questionnaire data (DQ) for the Netherlands | | | | |  |
| --- | --- | --- | --- | --- | --- |
|  | **24-HDR (N = 4,567)** | | **DQ (N = 39,036)** | |  |
|  | **Pearson correlation coefficient*** | **Weighted κ** | **Pearson correlation coefficient*** | **Weighted κ** | |
| Energy (kcal/day) | 0.97 | 0.85 | 0.98 | 0.89 |  |
| Water (g) | 1.00 | 0.97 | 1.00 | 0.98 |  |
| Total fats (g) | 0.94 | 0.77 | 0.98 | 0.89 |  |
| Fatty acids, total saturated (g) | 0.91 | 0.72 | 0.98 | 0.87 |  |
| Fatty acids, total monounsaturated (g) | 0.88 | 0.70 | 0.95 | 0.81 |  |
| Fatty acids, total polyunsaturated (g) | 0.87 | 0.68 | 0.94 | 0.77 |  |
| Cholesterol (mg) | 0.92 | 0.72 | 0.94 | 0.81 |  |
| Total proteins (g) | 0.92 | 0.74 | 0.97 | 0.84 |  |
| Carbohydrates (g) | 0.96 | 0.81 | 0.96 | 0.85 |  |
| Sugar, total (g) | 0.93 | 0.77 | 0.92 | 0.82 |  |
| Starch (g) | 0.60 | 0.38 | 0.82 | 0.63 |  |
| Dietary fiber, total (g) | 0.83 | 0.66 | 0.98 | 0.88 |  |
| Alcohol (g) | 1.00 | 0.98 | 1.00 | 0.98 |  |
| Calcium, Ca (mg) | 0.94 | 0.78 | 0.98 | 0.88 |  |
| Iron, Fe (mg) | 0.77 | 0.57 | 0.87 | 0.68 |  |
| Potassium, K (mg) | 0.89 | 0.71 | 0.97 | 0.83 |  |
| Magnesium, Mg (mg) | 0.83 | 0.69 | 0.95 | 0.78 |  |
| Phosphorus, P (mg) | 0.92 | 0.76 | 0.96 | 0.83 |  |
| Vitamin D (µg) | 0.59 | 0.29 | 0.66 | 0.46 |  |
| Vitamin E (alpha-tocopherol) (mg) | 0.72 | 0.52 | 0.61 | 0.45 |  |
| Retinol (µg) | 0.74 | 0.70 | 0.87 | 0.68 |  |
| Beta-carotene (µg) | 0.93 | 0.67 | 0.98 | 0.84 |  |
| Thiamin, B1 (mg) | 0.79 | 0.59 | 0.73 | 0.62 |  |
| Riboflavin, B2 (mg) | 0.85 | 0.64 | 0.90 | 0.70 |  |
| Cobalamin, B12 (µg) | 0.84 | 0.65 | 0.86 | 0.69 |  |
| Vitamin B6 (mg) | 0.77 | 0.65 | 0.78 | 0.73 |  |
| Vitamin C (mg) | 0.94 | 0.78 | 0.97 | 0.84 |  |
| Folate, food (µg) | 0.83 | 0.67 | 0.89 | 0.73 |  |
| *Pearson correlation coefficients for the 28 nutrient intakes measured by the USNDB and the ENDB were significant at the level of p < 0.001 for all nutrients | | | | |  |

| **Table 2Sf:** Pearson correlation coefficients and weighted kappas (κ) for dietary intakes of 28 nutrients of the U.S. nutrient database (USNDB) and the EPIC nutrient database (ENDB), reported for the 24-hour dietary recall data (24-HDR) and the dietary questionnaire data (DQ) for Germany | | | | |  |
| --- | --- | --- | --- | --- | --- |
|  | **24-HDR (N = 4,418)** | | **DQ (N = 52,013)** | |  |
|  | **Pearson correlation coefficient*** | **Weighted κ** | **Pearson correlation coefficient*** | **Weighted κ** | |
| Energy (kcal/day) | 0.94 | 0.81 | 0.99 | 0.90 |  |
| Water (g) | 1.00 | 0.96 | 1.00 | 0.98 |  |
| Total fats (g) | 0.91 | 0.76 | 0.98 | 0.87 |  |
| Fatty acids, total saturated (g) | 0.91 | 0.75 | 0.97 | 0.85 |  |
| Fatty acids, total monounsaturated (g) | 0.86 | 0.69 | 0.97 | 0.86 |  |
| Fatty acids, total polyunsaturated (g) | 0.82 | 0.64 | 0.93 | 0.78 |  |
| Cholesterol (mg) | 0.87 | 0.73 | 0.93 | 0.78 |  |
| Total proteins (g) | 0.90 | 0.73 | 0.96 | 0.83 |  |
| Carbohydrates (g) | 0.91 | 0.79 | 0.98 | 0.89 |  |
| Sugar, total (g) | 0.94 | 0.79 | 0.97 | 0.87 |  |
| Starch (g) | 0.32 | 0.21 | 0.67 | 0.50 |  |
| Dietary fiber, total (g) | 0.87 | 0.70 | 0.96 | 0.82 |  |
| Alcohol (g) | 0.97 | 0.78 | 0.99 | 0.94 |  |
| Calcium, Ca (mg) | 0.82 | 0.63 | 0.91 | 0.72 |  |
| Iron, Fe (mg) | 0.74 | 0.55 | 0.84 | 0.69 |  |
| Potassium, K (mg) | 0.93 | 0.77 | 0.98 | 0.87 |  |
| Magnesium, Mg (mg) | 0.81 | 0.61 | 0.92 | 0.76 |  |
| Phosphorus, P (mg) | 0.90 | 0.73 | 0.96 | 0.83 |  |
| Vitamin D (µg) | 0.44 | 0.38 | 0.76 | 0.52 |  |
| Vitamin E (alpha-tocopherol) (mg) | 0.65 | 0.51 | 0.80 | 0.63 |  |
| Retinol (µg) | 0.70 | 0.72 | 0.85 | 0.71 |  |
| Beta-carotene (µg) | 0.94 | 0.62 | 0.96 | 0.79 |  |
| Thiamin, B1 (mg) | 0.75 | 0.56 | 0.90 | 0.72 |  |
| Riboflavin, B2 (mg) | 0.84 | 0.62 | 0.91 | 0.71 |  |
| Cobalamin, B12 (µg) | 0.92 | 0.61 | 0.92 | 0.75 |  |
| Vitamin B6 (mg) | 0.86 | 0.71 | 0.93 | 0.82 |  |
| Vitamin C (mg) | 0.90 | 0.72 | 0.95 | 0.83 |  |
| Folate, food (µg) | 0.79 | 0.61 | 0.90 | 0.72 |  |
| *Pearson correlation coefficients for the 28 nutrient intakes measured by the USNDB and the ENDB were significant at the level of p < 0.001 for all nutrients | | | | |  |

| **Table 2Sg:** Pearson correlation coefficients and weighted kappas (κ) for dietary intakes of 28 nutrients of the U.S. nutrient database (USNDB) and the EPIC nutrient database (ENDB), reported for the 24-hour dietary recall data (24-HDR) and the dietary questionnaire data (DQ) for Sweden | | | | |
| --- | --- | --- | --- | --- |
|  | **24-HDR (N = 6,132)** | | **DQ (N = 52,750)** | |
|  | **Pearson correlation coefficient*** | **Weighted κ** | **Pearson correlation coefficient*** | **Weighted κ** |
| Energy (kcal/day) | 0.93 | 0.80 | 0.99 | 0.90 |
| Water (g) | 1.00 | 0.95 | 1.00 | 0.98 |
| Total fats (g) | 0.95 | 0.83 | 0.99 | 0.91 |
| Fatty acids, total saturated (g) | 0.91 | 0.74 | 0.96 | 0.84 |
| Fatty acids, total monounsaturated (g) | 0.90 | 0.75 | 0.98 | 0.87 |
| Fatty acids, total polyunsaturated (g) | 0.85 | 0.68 | 0.87 | 0.68 |
| Cholesterol (mg) | 0.91 | 0.74 | 0.97 | 0.87 |
| Total proteins (g) | 0.92 | 0.76 | 0.98 | 0.87 |
| Carbohydrates (g) | 0.88 | 0.74 | 0.96 | 0.84 |
| Sugar, total (g) | 0.92 | 0.74 | 0.97 | 0.84 |
| Starch (g) | 0.33 | 0.23 | 0.61 | 0.42 |
| Dietary fiber, total (g) | 0.77 | 0.67 | 0.92 | 0.79 |
| Alcohol (g) | 0.99 | 0.94 | 0.99 | 0.94 |
| Calcium, Ca (mg) | 0.94 | 0.80 | 0.97 | 0.86 |
| Iron, Fe (mg) | 0.62 | 0.52 | 0.81 | 0.63 |
| Potassium, K (mg) | 0.93 | 0.76 | 0.98 | 0.86 |
| Magnesium, Mg (mg) | 0.74 | 0.65 | 0.93 | 0.79 |
| Phosphorus, P (mg) | 0.88 | 0.74 | 0.96 | 0.84 |
| Vitamin D (µg) | 0.41 | 0.31 | 0.59 | 0.39 |
| Vitamin E (alpha-tocopherol) (mg) | 0.71 | 0.53 | 0.58 | 0.57 |
| Retinol (µg) | 0.71 | 0.63 | 0.63 | 0.63 |
| Beta-carotene (µg) | 0.86 | 0.79 | 0.97 | 0.91 |
| Thiamin, B1 (mg) | 0.41 | 0.58 | 0.90 | 0.73 |
| Riboflavin, B2 (mg) | 0.83 | 0.65 | 0.92 | 0.75 |
| Cobalamin, B12 (µg) | 0.78 | 0.52 | 0.87 | 0.67 |
| Vitamin B6 (mg) | 0.71 | 0.58 | 0.90 | 0.73 |
| Vitamin C (mg) | 0.90 | 0.76 | 0.94 | 0.81 |
| Folate, food (µg) | 0.83 | 0.63 | 0.92 | 0.74 |
| *Pearson correlation coefficients for the 28 nutrient intakes measured by the USNDB and the ENDB were significant at the level of p < 0.001 for all nutrients | | | | |

| **Table 2Sh:** Pearson correlation coefficients and weighted kappas (κ) for dietary intakes of 28 nutrients of the U.S. nutrient database (USNDB) and the EPIC nutrient database (ENDB), reported for the 24-hour dietary recall data (24-HDR) and the dietary questionnaire data (DQ) for Denmark | | | | |
| --- | --- | --- | --- | --- |
|  | **24-HDR (N = 3,918)** | | **DQ (N = 55,860)** | |
|  | **Pearson correlation coefficient*** | **Weighted κ** | **Pearson correlation coefficient*** | **Weighted κ** |
| Energy (kcal/day) | 0.97 | 0.86 | 0.98 | 0.89 |
| Water (g) | 1.00 | 0.97 | 1.00 | 0.99 |
| Total fats (g) | 0.96 | 0.83 | 0.98 | 0.88 |
| Fatty acids, total saturated (g) | 0.91 | 0.72 | 0.93 | 0.77 |
| Fatty acids, total monounsaturated (g) | 0.92 | 0.77 | 0.96 | 0.83 |
| Fatty acids, total polyunsaturated (g) | 0.87 | 0.70 | 0.89 | 0.70 |
| Cholesterol (mg) | 0.91 | 0.78 | 0.98 | 0.85 |
| Total proteins (g) | 0.96 | 0.81 | 0.99 | 0.92 |
| Carbohydrates (g) | 0.93 | 0.82 | 0.95 | 0.87 |
| Sugar, total (g) | 0.83 | 0.72 | 0.86 | 0.77 |
| Starch (g) | 0.45 | 0.22 | 0.48 | 0.31 |
| Dietary fiber, total (g) | 0.94 | 0.78 | 0.94 | 0.81 |
| Alcohol (g) | 0.99 | 0.96 | 0.99 | 0.97 |
| Calcium, Ca (mg) | 0.96 | 0.80 | 0.98 | 0.87 |
| Iron, Fe (mg) | 0.77 | 0.61 | 0.85 | 0.69 |
| Potassium, K (mg) | 0.95 | 0.80 | 0.97 | 0.85 |
| Magnesium, Mg (mg) | 0.89 | 0.72 | 0.96 | 0.80 |
| Phosphorus, P (mg) | 0.95 | 0.80 | 0.98 | 0.87 |
| Vitamin D (µg) | 0.55 | 0.55 | 0.65 | 0.53 |
| Vitamin E (alpha-tocopherol) (mg) | 0.73 | 0.61 | 0.76 | 0.60 |
| Retinol (µg) | 0.87 | 0.68 | 0.94 | 0.74 |
| Beta-carotene (µg) | 0.99 | 0.81 | 1.00 | 0.94 |
| Thiamin, B1 (mg) | 0.80 | 0.60 | 0.90 | 0.72 |
| Riboflavin, B2 (mg) | 0.87 | 0.65 | 0.91 | 0.69 |
| Cobalamin, B12 (µg) | 0.94 | 0.73 | 0.98 | 0.86 |
| Vitamin B6 (mg) | 0.89 | 0.70 | 0.96 | 0.83 |
| Vitamin C (mg) | 0.96 | 0.83 | 0.98 | 0.86 |
| Folate, food (µg) | 0.87 | 0.68 | 0.93 | 0.77 |
| *Pearson correlation coefficients for the 28 nutrient intakes measured by the USNDB and the ENDB were significant at the level of p < 0.001 for all nutrients | | | | |

| **Table 2Si:** Pearson correlation coefficients and weighted kappas (κ) for dietary intakes of 28 nutrients of the U.S. nutrient database (USNDB) and the EPIC nutrient database (ENDB), reported for the 24-hour dietary recall data (24-HDR) and the dietary questionnaire data (DQ) for Norway | | | | |
| --- | --- | --- | --- | --- |
|  | **24-HDR (N = 1,798)** | | **DQ (N = 36,448)** | |
|  | **Pearson correlation coefficient*** | **Weighted κ** | **Pearson correlation coefficient*** | **Weighted κ** |
| Energy (kcal/day) | 0.96 | 0.84 | 1.00 | 0.94 |
| Water (g) | 1.00 | 0.97 | 1.00 | 0.99 |
| Total fats (g) | 0.93 | 0.79 | 0.99 | 0.91 |
| Fatty acids, total saturated (g) | 0.91 | 0.76 | 0.98 | 0.87 |
| Fatty acids, total monounsaturated (g) | 0.89 | 0.72 | 0.95 | 0.82 |
| Fatty acids, total polyunsaturated (g) | 0.85 | 0.66 | 0.93 | 0.78 |
| Cholesterol (mg) | 0.84 | 0.68 | 0.97 | 0.84 |
| Total proteins (g) | 0.88 | 0.75 | 0.99 | 0.90 |
| Carbohydrates (g) | 0.95 | 0.83 | 0.99 | 0.93 |
| Sugar, total (g) | 0.93 | 0.79 | 0.97 | 0.86 |
| Starch (g) | 0.74 | 0.48 | 0.88 | 0.69 |
| Dietary fiber, total (g) | 0.89 | 0.75 | 0.99 | 0.91 |
| Alcohol (g) | 1.00 | 0.97 | 1.00 | 0.98 |
| Calcium, Ca (mg) | 0.93 | 0.77 | 0.97 | 0.83 |
| Iron, Fe (mg) | 0.63 | 0.61 | 0.76 | 0.61 |
| Potassium, K (mg) | 0.88 | 0.78 | 0.99 | 0.91 |
| Magnesium, Mg (mg) | 0.82 | 0.67 | 0.97 | 0.85 |
| Phosphorus, P (mg) | 0.91 | 0.80 | 0.99 | 0.92 |
| Vitamin D (µg) | 0.53 | 0.36 | 0.97 | 0.73 |
| Vitamin E (alpha-tocopherol) (mg) | 0.67 | 0.47 | 0.44 | 0.41 |
| Retinol (µg) | 0.84 | 0.79 | 0.99 | 0.88 |
| Beta-carotene (µg) | 0.94 | 0.82 | 1.00 | 0.93 |
| Thiamin, B1 (mg) | 0.32 | 0.56 | 0.95 | 0.81 |
| Riboflavin, B2 (mg) | 0.74 | 0.56 | 0.91 | 0.71 |
| Cobalamin, B12 (µg) | 0.75 | 0.66 | 0.93 | 0.75 |
| Vitamin B6 (mg) | 0.78 | 0.63 | 0.94 | 0.78 |
| Vitamin C (mg) | 0.95 | 0.82 | 0.98 | 0.86 |
| Folate, food (µg) | 0.88 | 0.71 | 0.96 | 0.82 |
| *Pearson correlation coefficients for the 28 nutrient intakes measured by the USNDB and the ENDB were significant at the level of p < 0.001 for all nutrients | | | | |
