# Supplementary material for: Comparing Calculated Nutrient Intakes Using Different Food Composition Databases: Results from the European Prospective Investigation into Cancer and Nutrition (EPIC) Cohort
Source: Nutrients. 2020 Sep 23;12(10):2906. doi: 10.3390/nu12102906 (PMC7650652; doi:10.3390/nu12102906)
Supplement: Supplementary file 1 [file nutrients-12-02906-s001.zip › Revision2_Nutrients_TableS3.docx]

**Comparing calculated nutrient intakes using different food composition databases: Results from the European prospective investigation into cancer and nutrition (EPIC) cohort**

Heleen Van Puyvelde, Aurora Perez-Cornago, Corinne Casagrande, Genevieve Nicolas, Vickà Versele, Guri Skeie, Matthias B. Schulze, Ingegerd Johansson, José María Huerta, Oliverio Andreina, Fulvio Ricceri, Jytte Halkjær, Pilar Amiano Etxezarreta, Koen Van Herck, Elisabete Weiderpass, Marc J. Gunter, Inge Huybrechts, on behalf of the EPIC Consortium

**Table S3:** Short overview of the U.S. nutrient database (USNDBB) and the EPIC nutrient database (ENDB) reference component-specific definition and standard analytical methods and approaches used

| **Table S3:** Short overview of the U.S. nutrient database (USNDBB) and the EPIC nutrient database (ENDB) reference component-specific definition and standard analytical methods and approaches used | | | |
| --- | --- | --- | --- |
| **Component name USNDBB** | **Component name ENDB** | **Reference component definition and analytical methods used in USNDBB (1)** | **Reference component definition and analytical methods used in ENDB (2)** |
| Energy (kcal) | Energy (kcal) | Analytical method:  Calculation based on the Atwater system (except for multi-ingredient processed foods), however slightly revised using specific calorie conversion factors for specific foods (3).  Fixed calorie factor for alcohol: 6.93 kcal/g  For multi-ingredient processed foods, kilocalories calculated by the manufacturer were prioritized over using calorie factors. | Analytical method:  Calculation based on the Atwater system (4). Sum of the factored contributions from fat, protein, carbohydrates and alcohol as reported in the ENDB: fat 37 kJ/g (9 kcal/g) protein: 17 kJ/g (4 kcal/g) carbohydrates: 17 kJ/g (4 kcal/g) alcohol: 29 kJ/g (7 kcal/g). |
| Water (g) | Water (g) | Analytical method:  Vacuum oven or forced air to determine dried mass | Analytical method: Drying method (all methods across EPIC countries comparable) to get the dry matter.  Water = 100 minus dry matter (and minus alcohol where appropriate)  Missing values: manual imputation |
| Protein (g) | Total proteins (g) | Analytical method:  Calculation based on the amount of total nitrogen in food  Specific nitrogen conversion factors were used for most food items (5).  Fixed nitrogen conversion factors of 6.25 for items that do not have a specific factor.  Analytical methods for nitrogen: AOAC 968.06 (4.2.04), 992.15 (39.1.16), and 990.03 (combustion); 991.20 (Kjeldahl) (6). | Analytical method:  Calculation based on the amount of nitrogen in food All analytical methods across EPIC countries comparable for nitrogen  Protein* = nitrogen multiplied by nitrogen conversion factor (NCF), where NCF = 6.25 for all foods.  Missing values: manual imputation  *Deviation from standardization: Protein values for recipes (Greece, Sweden) and for Germany, where nitrogen and/or the NCF were systematically missing due to missing documentation in the national FCDB. |
| Total lipid (fat) (g) | Total fats (g) | Analytical method:  Analytical methods for total lipid (fat) are determined by gravimetric methods, including acid hydrolysis and extraction methods using a mixed solvent system of chloroform and methanol. Older values may have been obtained by ether extraction. | Analytical method:  Analytical analysis of total lipids, not calculations based on the sum of fatty acids.  The choice of the method depends on the food group (e.g. alkaline extraction is preferred for dairy products, Soxhlet method is unreliable to determine fat in)  Missing values: manual imputation  *Deviation from standardization: a few non-comparable values (improper methods used) in Denmark, France, Norway, Spain, Greece and UK |
| Cholesterol (mg) | Cholesterol (mg) | Analytical method:  Analytical methods for cholesterol obtained by gas liquid chromatographic procedures or by a GC method without derivitization (recent meat data).  Foods containing only plant products, cholesterol is assumed zero. | Analytical method:  All methods across the EPIC countries were considered comparable  Missing values: logical zeros for food of vegetal origin and manual imputation for remaining missing values |
| Fatty acids, total saturated (g) | Total saturated fatty acids (g) | Definition:  Expressed as g fatty acid per 100 g edible food (and do not represent fatty acids as triglycerides)  Analytical method:  No information If data on saturated (SFA), monounsaturated (MUFA), and polyunsaturated fatty acids (PUFA) is available, but data on individual fatty within those fatty acid classes is missing, then individual fatty acids are calculated from the fatty acids of the food item and normalized to the mean fat value of the food item. | Definition: Total of straight-chain saturated fatty acids, down to C4; expressed as g fatty acid per 100 g edible food  Analytical method:  No information  Missing values: imputed by profile calculation of foods with similar fat content, using their relative proportion of fatty acids (Spain, Greece, UK) or edited, copied or averaged based on equivalent foods from the national or a foreign FCDB. |
| Fatty acids, total monounsaturated (g) | Total monounsaturated fatty acids (g) | see total saturated fatty acids | Definition:  Total of monounsaturated fatty acids. Could contain trans-fatty acids |
| Fatty acids, total polyunsaturated (g) | Total polyunsaturated fatty acids (g) | see total saturated fatty acids | Definition:  Total of polyunsaturated fatty acids. Could contain trans-fatty acids |
| Carbohydrate, by difference (g) | Carbohydrates (g) | Definition:  Total carbohydrate, including dietary fiber  Analytical method: Calculation based on the difference between 100 and the sum of the percentages of water, protein, total lipid (fat), ash, and, when present, alcohol (= the difference method) | Definition:  Glycemic carbohydrate, excluding dietary fiber. Definitions in- or excluding the oligosaccharides were used interchangeably.  Analytical method: Calculation based on the sum of analyzed fractions, excluding dietary fiber: *carbohydrate (g) = the sum of starch (g), disaccharides (g) and monosaccharides (g)  *Deviation from standardization: Denmark: glycemic carbohydrate = total carbohydrate (including fiber) minus dietary fiber; Greece: glycemic carbohydrate by difference method; for a few cases in Spain and UK: glycemic carbohydrate = starch plus sugar |
| Fiber, total dietary (g) | Total dietary fiber (g) | Definition:  Total dietary fiber  Analytical method:  enzymatic gravimetric methods (985.29 ‘Total Dietary Fiber in Foods’ or 991.43 ‘Total, Soluble, and Insoluble Dietary Fiber in Foods’ of the AOAC (6). | Definition:  Total dietary fiber, including lignin and resistant starch  Analytical method:  The gravimetric method was used as the standard analytical method for total dietary fiber for ENDB. Comparability with other methods depends on the food group: for fruit and vegetables the Englyst method was considered comparable (includes only the non-starch polysaccharides), Southgate values were considered obsolete and The Hellendorn method by gravimetry was considered non-comparable  Missing values: logical zeros for food of animal origin and manual imputation of foreign values for remaining missing values |
| Sugars, total (g) | Sugar (g) | Definition:  Total sugars, including monosaccharides and disaccharides  Analytical method:  Calculation based on the sum of individual monosaccharides (galactose, glucose, and fructose) and disaccharides (sucrose, lactose, and maltose).  Analytical methods used for individual sugars were: liquid chromatography (AOAC 982.14). Earlier values were determined using high performance liquid chromatography (HPLC) or gas-liquid chromatography (GLC).  Missing values: imputation or borrowed from manufacturers and trade associations. | Definition:  Mono- and disaccharides, excluding trisaccharides and higher  oligosaccharides.  Analytical method:  Calculation based on the sum of galactose, glucose, fructose, sucrose, lactose, and maltose. All methods across the EPIC countries were considered comparable.  *Deviation from standardization: Not all countries included galactose in the national definition. Missing values: imputed by profile calculation of foods with similar carbohydrate content, using their relative proportion of sugars |
| Starch (g) | Starch (g) | Analytic method:  Analytical methods used for starch: enzymatic colorimetric  (AOAC 966.11) or by a polarometric method for sampling and analysis. | Definition: Starches, including dextrins and glycogen. Analytical method: All methods across the EPIC countries were considered comparable Missing values: Sweden and UK: calculated as the difference between carbohydrates and sugars; Greece: profile calculation of foods with similar carbohydrate content, using their relative proportion of starch |
| Alcohol, ethyl (g) | Alcohol (g) | Definition:  Ethyl alcohol (= ethanol)  Analytical method:  No specific information | Definition:  Ethanol Analytical methods: All analytic methods were considered comparable. |
| Calcium, Ca (mg) | Calcium (mg) | Analytical method:  Levels of minerals were determined by inductively coupled plasma emission spectrophotometry (AOAC 984.27) or by atomic absorption (AOAC 985.35). | Analytical method: All methods across the EPIC countries were considered comparable, except dry ashing.  Missing values: manual imputation. |
| Iron, Fe (mg) | Iron (mg) | Analytical method:  Levels of minerals were determined by inductively coupled plasma emission spectrophotometry (AOAC 984.27) or by atomic absorption (AOAC 985.35). | Definition:  Haem and non-haem iron. |
| Magnesium, Mg (mg) | Magnesium (mg) | Analytical method:  Levels of minerals were determined by inductively coupled plasma emission spectrophotometry (AOAC 984.27) or by atomic absorption (AOAC 985.35). | / |
| Phosphorus, P (mg) | Phosphorus (mg) | Analytical method:  Levels of minerals were determined by inductively coupled plasma emission spectrophotometry (AOAC 984.27) or calorimetrically (AOAC 2.019, 2.095 and 7.098) | / |
| Potassium, K (mg) | Potassium (mg) | Analytical method:  Levels of minerals were determined by inductively coupled plasma emission spectrophotometry (AOAC 984.27) or by atomic absorption (AOAC 985.35). | / |
| Vitamin C, total ascorbic acid (mg) | Vitamin C (mg) | Definition:  Total ascorbic acid  Analytical method:  The majority of the levels of vitamin C was determined by microfluorometric method (AOAC 967.22). Older, not yet updated values were determined by the dichloroindophenol method (AOAC 967.21) and are primarily for reduced ascorbic acid. | Definition:  Including L-ascorbic acid and dehydroascorbic acid, however because dehydroascorbic acid is unstable and amounts are small in fresh foods L-ascorbic acid was assumed to be equivalent to vitamin C  Analytical method: Calculation based on the sum of L-ascorbic acid and dehydroascorbic acid.  All analytic methods across the EPIC countries were considered comparable  Missing values: manual imputation |
| Thiamine (mg) | Thiamine (vitamin B1) (mg) | Analytical method:  Chemical determination by the fluorometric method (AOAC 942.23). | Definition:  Vitamin–hydrochloride  Missing values: manual imputation |
| Riboflavin (mg) | Riboflavin (vitamin B2) (mg) | Analytical method:  Chemical determination by the fluorometric method (AOAC 942.23) or by a microbiological method (AOAC 940.33). | Analytical method:  All analytic methods across the EPIC countries were comparable  Missing values: manual imputation |
| Vitamin B6 (mg) | Vitamin B6 (mg) | Analytical method:  Microbiological method (AOAC 961.15). | Definition:  Vitamin–hydrochloride  Missing values: manual imputation |
| Folate, total (µg) | Folate, total (µg) | Definition:  Food folate (other values are reported as well for folic acid, and total folate reported in μg and as dietary folate equivalents)  Analytical method:  Folate naturally occurring in foods: trienzyme microbiological procedure.  Enriched foods: a microbiological procedure without enzymes to estimate the amount of added folic acid. Food folate is then calculated as the difference of total folate and added folic acid. | Definition:  Naturally occurring food folates (non-fortified foods)  Analytical method:  Microbiological assay (MA) is the reference method.  Missing values: 100% completed. When no country specific MA values could be found, any other available European or US folate data for that food or similar food was borrowed. As a last choice, even other values than MA values were accepted. |
| Vitamin B12 (µg) | Cobalamin (vitamin B12) (µg) | Analytical method:  Microbiological method (AOAC 952.20).  Foods containing only plant products, vitamin B12 is assumed to be zero. | Missing values: 100% completed except for Italy (imputed) |
| Retinol (µg) | Retinol (performed vitamin A) (µg) | Definition:  Values for retinol (µg) and vitamin A (μg of retinol activity equivalents (RAEs)) are reported. RAE is used as the unit for expressing vitamin A activity.  Analytical method:  For RAE, calculation is based on the content of individual carotenoids (β-carotene, α-carotene, and β-cryptoxanthin) using the appropriate factors.  1µ g RAE = 1 μg of all-trans-retinol, 12 μg of all trans-β-carotene, or 24 μg of other provitamin A carotenoids.  Missing values: μg RAE and μg of retinol are calculated by dividing the international unit (IU) value by a food specific factor. | Analytical method:  Calculation based on the sum of retinoid as all-trans-retinol, with or without application of activity factors: 75% for 13-cis-retinol, 90% for Retinaldehyde.  High-performance liquid chromatography (HPLC) is the reference method (not colorimetric methods).  Missing values: logical zeros for food of vegetal origin (the UK and the Netherlands), manual imputation for remaining missing values. |
| Carotene, beta (µg) | Beta-carotene (µg) | Analytical method:  HPLC (AOAC 941.15 or Chromatographic techniques for carotenoid separation). | Definition:  Beta-carotene is the reference value, however beta-carotene equivalent values are considered comparable except for fruits, vegetables, legumes and derived foods.  Analytical method:  HPLC is the reference method Missing values: manual imputation (100% for France and Italy) or beta-carotene equivalent values were reported; For the UK and the Netherlands missing values for plant foods were calculated as vitamin A multiplied by six |
| Vitamin E (alpha-tocopherol) (mg) | Vitamin E (mg) | Definition:  α-tocopherol  β, γ, and δ-tocopherol are not considered to contribute to vitamin E activity, however, their values as well as values for α-, β, γ, and δ-tocotrienol are included in the database since release 26 (2013).  Analytical method:  Gas-liquid chromatography (GLC) or high-performance liquid chromatography (HPLC) | Definition:  Vitamin E is the reference value, however Vitamin E and a-tocopherol values were considered comparable in animal products, whereas in plant foods other tocopherols and tocotrienols make a substantial contribution.  Analytical method:  Vitamin E (α-tocopherol equivalents) = α-tocopherol, 0.40 x β-tocopherol, 0.10 x γ-tocopherol, 0.01 x δ-tocopherol, 0.30 x α-tocotrienol,0.05 x β-tocotrienol, 0.01 x γ–tocotrienol ^h^.  All analytical methods were considered comparable for α-tocopherol and vitamin E.  Missing values: Sweden and Norway only had values for α-tocopherol. Manual imputation of vitamin E or α-tocopherol values were reported. |
| Vitamin D (µg) | Vitamin D (µg) | Definition:  Vitamin D2 and vitamin D3  Analytical method:  Calculation based on the sum of vitamin D2 and vitamin D3.  No single analytical method is required (due to lack of a validated analytical methodology), however all participating laboratories must quality control analyses that falls within an acceptable range of values. Missing values: logical zeros for food of vegetal origin, except for mushrooms. Data borrowed from scientific literature or from other food composition databases, or estimated by other USNDBB imputation methods | Definition:  Cholecalciferol (vitamin D3) and ergocalciferol (vitamin D2, found in wild mushrooms only). Definitions in- or excluding the 25-OH-Vit D (found in meat only) were used interchangeably. Analytical method:  All analytical methods were considered comparable as comparable, except for bioassays and chemical methods to determines values of vitamin D in fish, eggs and fats, data  Missing values: manual imputation |
| General remarks | General remarks | For recipes, the total nutrient content of the recipe equals the sum of the nutrient content in each single ingredient. Conversion factors were applied at the ingredient level.  For multi-ingredient processed foods, kilocalories and nutrient content calculated by the manufacturer were prioritized over using calorie factors or analytical methods respectively. Missing values: imputed according to scientific principles or calculated using the recipe or formulation modules within the databank system. | Country-specific recipes for multi-ingredient foods were developed. The total nutrient content of the recipe equals the sum of the nutrient content in each single ingredient. Conversion factors were applied at the ingredient and recipe level  Missing values: assumption that it is better to approximate nutrient values than to leave them as missing, and thus completion of missing values as much as possible was pursued. Logical zeros were identified for foods containing none or only traces of a specific nutrient. ‘Real’ missing values were imputed by borrowing values from similar foods in the same national dataset or from the same food in foreign databases. |
| (1) U.S. Department of Agriculture, Agricultural Research Service. 2013. USDA National Nutrient Database for Standard Reference, Release 26. Accessed March 18, 2020. <http://www.ars.usda.gov/ba/bhnrc/ndl>  (2) Slimani N, Deharveng G, Unwin I, et al. The EPIC nutrient database project (ENDB): a first attempt to standardize nutrient databases across the 10 European countries participating in the EPIC study. *Eur J Clin Nutr*. 2007;61(9):1037-1056. doi:10.1038/sj.ejcn.1602679  (3) Merrill A, Watt B. Energy value of foods: Basis and derivation (agriculture handbook no. 74). *Washington: US government printing office*. 1973;  (4) Atwater WO. *Principles of nutrition and nutritive value of food*. US Dept. of Agriculture; 1910.  (5) Jones DB. *Factors for converting percentages of nitrogen in foods and feeds into percentages of proteins*. US Department of Agriculture; 1931.  (6) AOAC International, Horwitz W, Latimer GW. *Official methods of analysis of AOAC International*. AOAC International; 2010. | | | |
